# Immune pathway activation in gastric cancers with LINE-1 retrotransposon overexpression and homologous recombination deficiency

**DOI:** 10.1038/s12276-026-01846-5

**Published:** 2026-09-07

**Authors:** Young Jin Kim, In-Pyo Baek, Bang Wool Eom, Min-Jung Seo, Heeyeon Joo, Yu Ri Choi, Min Kyoung Park, Jihye Oh, Boram Lee, Seung Min Ham, Nak-Hyeon Choi, Seung-Pyo Hong, Jaeyong Choi, Sookyung Kim, Seong-Ho Kong, Hyuk-Joon Lee, Yun-Suhk Suh, Jinhwa Kong, Je-Keun Rhee, Sang-Won Lee, Hong Man Yoon, Hark Kyun Kim, Young-Woo Kim, Jong-Il Kim, Bong-Jo Kim, Han Kwang Yang

**Affiliations:** 1 Division of Genome Science, Department of Precision Medicine, National Institute of Health, Cheongju, Republic of Korea; 2 Department of Bioinformatics & Life Science, Soongsil University, Seoul, Republic of Korea; 3 National Cancer Center, Goyang, Republic of Korea; 4 Department of Biomedical Sciences, Seoul National University College of Medicine, Seoul, Republic of Korea; 5 Genomic Medicine Institute, Medical Research Center, Seoul National University, Seoul, Republic of Korea; 6 Department of Biochemistry and Molecular Biology, Seoul National University College of Medicine, Seoul, Republic of Korea; 7 Department of Surgery, Seoul National University Hospital and Seoul National University College of Medicine, Seoul, Republic of Korea; 8 Cancer Research Institute, Seoul National University College of Medicine, Seoul, Korea; 9 Department of Chemistry and National Research Laboratory for Convergence Degradation Biology, Korea University, Seoul, Republic of Korea; 10 Present Address: Kangwon National University School of Medicine, Chuncheon, Republic of Korea; 11 Present Address: National Cancer Center, Goyang, Republic of Korea

**Keywords:** Cancer genomics, Genome informatics

## Abstract

There are only a few whole genome sequencing studies of human gastric cancer (GC) conducted so far. We performed comprehensive whole genome, bulk RNA, and methylation sequencing analyses of 100 samples of GC and adjacent normal tissue. In a smaller non-EBV/non-MSI subset (*n* = 23), we also performed proteomic profiling by mass spectrometry. We validated the proteomic findings in an independent dataset. Using this unprecedented dataset of human GC samples, we examined the extent of chromothripsis, homologous recombination deficiency, and retrotransposition, and correlated these events with patient outcomes. We found that chromothripsis occurred in 22% of GCs and correlated with poor prognosis. Multichromosomal chromothripsis was associated with a particularly high risk of death. Based on copy number (CN) signature analysis, we identified a distinct non-CN9 subgroup with significantly worse outcomes. Homologous recombination deficiency was present in 4% of GCs and was associated with overexpression of immune signaling pathways. Somatic retrotransposition events were most strongly associated with global hypomethylation. We also identified *BYSL* as a putative oncogenic driver within the 6p21 locus whose amplification is associated with poor prognosis. Collectively, our findings provide novel insights into the dysregulation of DNA stability and repair and their clinical relevance in human GCs.

## Chromothripsis linked to poor prognosis in gastric cancer

Gastric cancer (GC) is a leading cause of cancer mortality, with chromosomal instability playing a crucial role in its development. This study explores the prognostic significance of chromothripsis, a form of genomic instability, in GC using whole genome sequencing and transcriptomic analysis of 100 GC samples. Researchers identified 38 chromothripsis events, with 22 tumors exhibiting at least one event, correlating with poor prognosis. The study also highlights the role of *BYSL*, a gene within the 6p21 amplicon, as a potential oncogene in GC. Chromothripsis was linked to extrachromosomal DNA formation, further impacting prognosis. In addition, homologous recombination deficiency was associated with immune gene overexpression, suggesting therapeutic targets. These findings underscore the importance of chromosomal instability in GC prognosis and suggest future research directions, including the potential for copy number signature analysis and homologous recombination deficiency-targeted therapies. This summary was initially drafted using artificial intelligence, then revised and fact-checked by the author.

## Introduction

Gastric cancer (GC) is the fourth most common cause of cancer death worldwide^1^. Chromosomal instability is a hallmark of cancer and is attributed to several distinct processes including telomere dysfunction and mitotic errors. Several distinct phenotypes resulting from these processes include chromothripsis and homologous recombination deficiency (HRD). Chromosomal instability has an important role in gastric carcinogenesis and is prognostic according to several studies including our own^2–4^. Whole genome sequencing (WGS) technology features specific types of genomic instability in detail, but only a few WGS studies have been performed in GC thus far^2,5,6^. Moreover, the follow-up period of these studies is inadequate for prognostication^2,5,6^.

Chromothripsis is a massive genomic rearrangement localized to isolated chromosomal regions that results from telomere dysfunction^7^. Chromothripsis is predictive of poor prognosis in many types of cancers, but its prognostic impact in GC remains unknown. One study found that chromothripsis was observed at a frequency of 4.8% (8/168) in GC, but its prognostic impact was not evaluated^2^. The Pan-Cancer Analysis of Whole Genomes (PCAWG) consortium study evaluated chromothripsis across multiple tissue types including GC, but it did not fully address the clinical relevance of these genomic events in GC because of the short follow-up duration (approximate median 6 months)^6^.

HRD is a major defect in DNA double-strand break repair and is linked to chromothripsis^8^. The frequency of HRD in GC varies according to the analytic platform^9^. WGS analyses outperform whole exome sequencing in detecting HRD^10^, but no WGS studies of GC have evaluated HRD.

We conducted comprehensive WGS and transcriptomic analyses of 100 frozen resected GC tissues (Supplementary Table 1) to characterize various types of genomic instability, focusing on their clinical relevance. Making the most of the relatively long follow-up duration of our GC tissue samples, we focused on the prognostic relevance and immune gene expression of each form of chromosomal instability.

## Materials and methods

We conducted whole genome, bulk RNA, and methylation sequencing analyses with 100 resected tumors and matched germline samples that were collected from Korean patients with GC. After light microscopic examination of the top slides, tumor-rich areas were macrodissected from the frozen tumor specimens for the isolation of genomic DNA and total RNA. Adjacent normal tissues were collected from the luminal side of the gastrectomy specimen at least 2 cm from the tumor border.

Fresh frozen gastric adenocarcinomas and matched adjacent normal tissue samples were obtained from 60 and 40 Korean patients with GC at Seoul National University Hospital (SNUH) (Seoul, Korea) and National Cancer Center (NCC) Hospital (Goyang, Korea), respectively. This study was approved by the SNUH and NCC Institutional Review Boards (SNUH C-1402-054-555 and NCC2017-0238).

Among 100 GCs, there were 68 male patients, 54 intestinal-type tumors, and 58 distal tumors. Most (88%) patients underwent curative resection, with 5, 25, and 58 being resected at American Joint Committee on Cancer (AJCC) 8th edition stage 1 (IB), 2 (IIA (*n* = 6)/IIB (*n* = 19)), and 3 (IIIA (*n* = 33)/IIIB (*n* = 11)/IIIC (*n* = 14)), respectively (Supplementary Table 1b).

Macrodissected frozen tumor and adjacent normal tissue were cryopulverized in liquid nitrogen and subjected to genomic DNA and RNA isolation. Genomic DNA samples were subjected to WGS (2 × 151 bp) and methylation sequencing (2 × 101 bp) using an Illumina NovaSeq 6000 system (Illumina). For methylation sequencing, bisulfite libraries were prepared using SureSelect XT Methyl-Seq workflow (Agilent). The total RNA (1 μg) of the same tissue was subjected to mRNA sequencing analysis using the same Illumina system following provided protocols for 2 × 101 sequencing. Reads were aligned to the human genome build GRCh37. For methylation sequencing analyses, PureBeta was used to adjust β_tumor_ values according to tumor purity^11^.

Protein lysate was isolated from the same tissue powder that was used to isolate genomic DNA and RNA. Tandem mass tag (TMT)-labeled peptides were pooled and concentrated using vacuum centrifugation. Basic pH reversed-phase liquid chromatography (RPLC) fractionation was then conducted to fractionate pooled TMT-labeled peptide samples into 24 peptide fractions using the 1260 Infinity HPLC system (Agilent). Each of the 24 peptide fractions (approximately 5 µg each) was individually separated by a modified nanoACQUITY UPLC (Waters) coupled online to a Q Exactive Orbitrap mass spectrometer (Thermo Fisher Scientific). TMT reporter ion intensity in each tumor was normalized to the intensity of corresponding universal reference of various tissue origin, followed by missing value imputation using Perseus^12^. See online Supplementary Information.

## Results

### Coding and non-coding drivers in GC

We analyzed the whole genome (mean coverages 29.6 and 47.0 for normal and tumor samples, respectively) and transcriptome (mean coverage 114.3 and 188.7 for normal and tumor samples, respectively) of 100 GCs, with a median follow-up of 103.5 months in surviving patients (range 3.0–230.4). Median tumor purity was estimated as 40%.

**Fig. 1:**
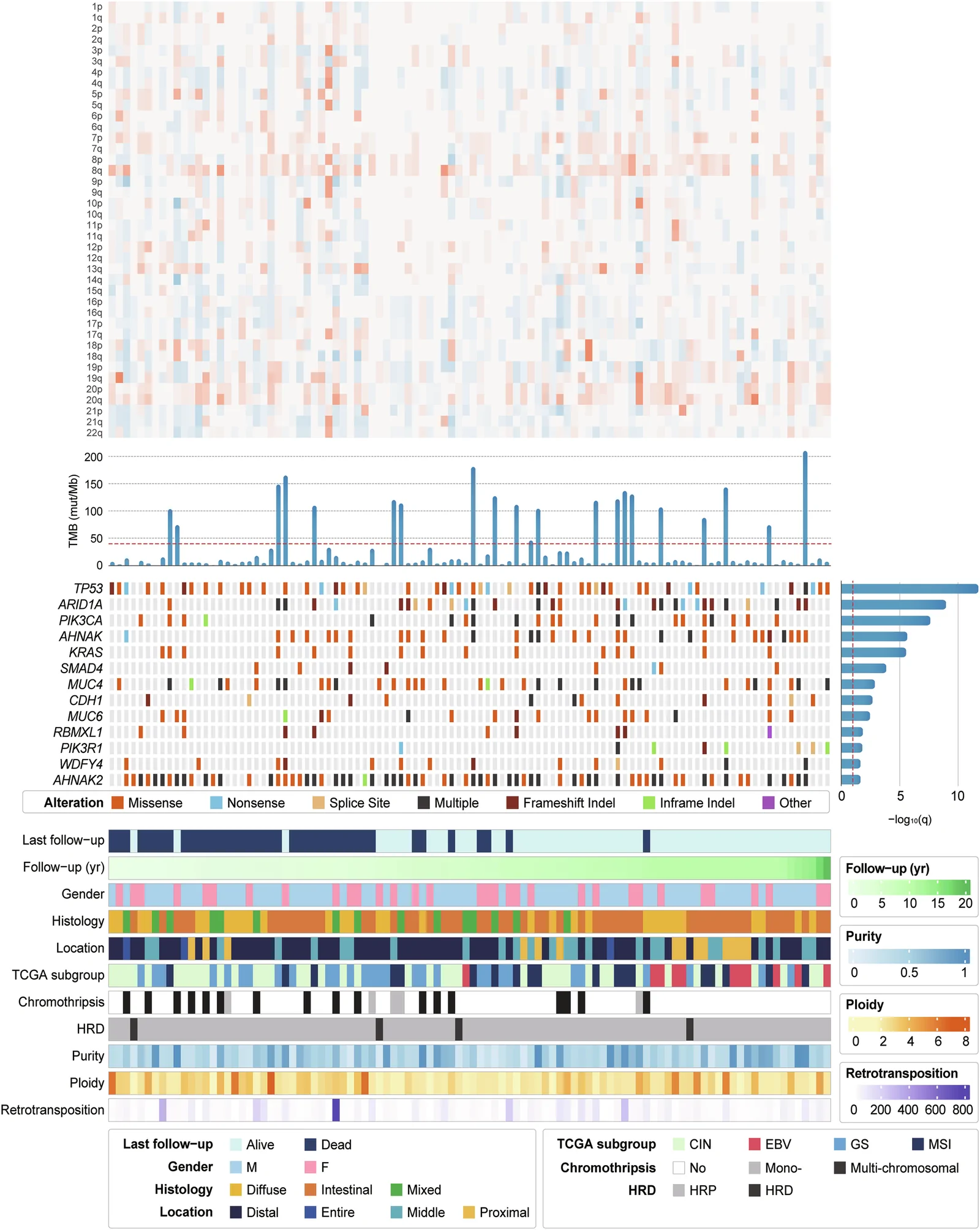
Significant somatic alterations in the genome of 100 gastric cancers (GCs). Top panel, Heatmap for arm-level copy number gains (*red*) and losses (*blue*) of 22 autosomes. Each column represents a GC, and each row represents a chromosome arm. All samples are ordered according to increasing follow-up duration from left to right. Top histogram, Tumor mutation burden (TMB) per sample. Hypermutated GCs (*n* = 21) were defined as TMB > 40 mut/Mb (cutoff shown as a red, dotted, horizontal line). All hypermutated GCs, except for two, exhibited high microsatellite instability (MSI). Middle panel, Heatmap for mutation events. Recurrent (*q* < 0.05) somatic mutations with evidence of protein expression according to global proteomic profiling are listed vertically by *q* value. Right histogram, Significance level of mutations as determined by log_10_ transformation of MutSig2CV *q* value. Red line, *q* = 0.05. CIN chromosomal instability, EBV Epstein–Barr virus, GS genomically stable, HRD homologous recombination deficiency, HRP homologous recombination proficient.

We identified 4,086,218 single nucleotide variations (SNVs) and 5,168,210 indels, and the median tumor mutation burden (TMB) of GC genomes was 7.6 mutations per megabase (mut/Mb) (range 0.4–194.5). When 79 non-hypermutated (TMB ≤ 40 mut/Mb) GCs were subjected to MutSig2CV v3.11^13^, 13 genes were significantly mutated with *q* < 0.05 (Fig. 1 and Supplementary Tables 2 and 3), and the recurrent somatic SNVs were similar to a list of recurrent mutations reported in The Cancer Genome Atlas Stomach Adenocarcinoma (TCGA–STAD) paper^14^. *TP53* mutation was observed in 48 patients (Supplementary Fig. 2). Hyperdiploidy (ploidy ≥ 3; *n* = 28) was poorly prognostic (*P* for log-rank = 0.002).

As our MutSig2CV analysis of non-coding mutations revealed no significantly recurrent mutations, we searched for non-coding mutations previously reported in other types of cancer^5^. Consequently, we found *TERT* promoter mutation (chr5:1,295,459 G > A) in a GC with transcriptional activation of *TERT*^15^ (Supplementary Fig. 4).

**Fig. 2:**
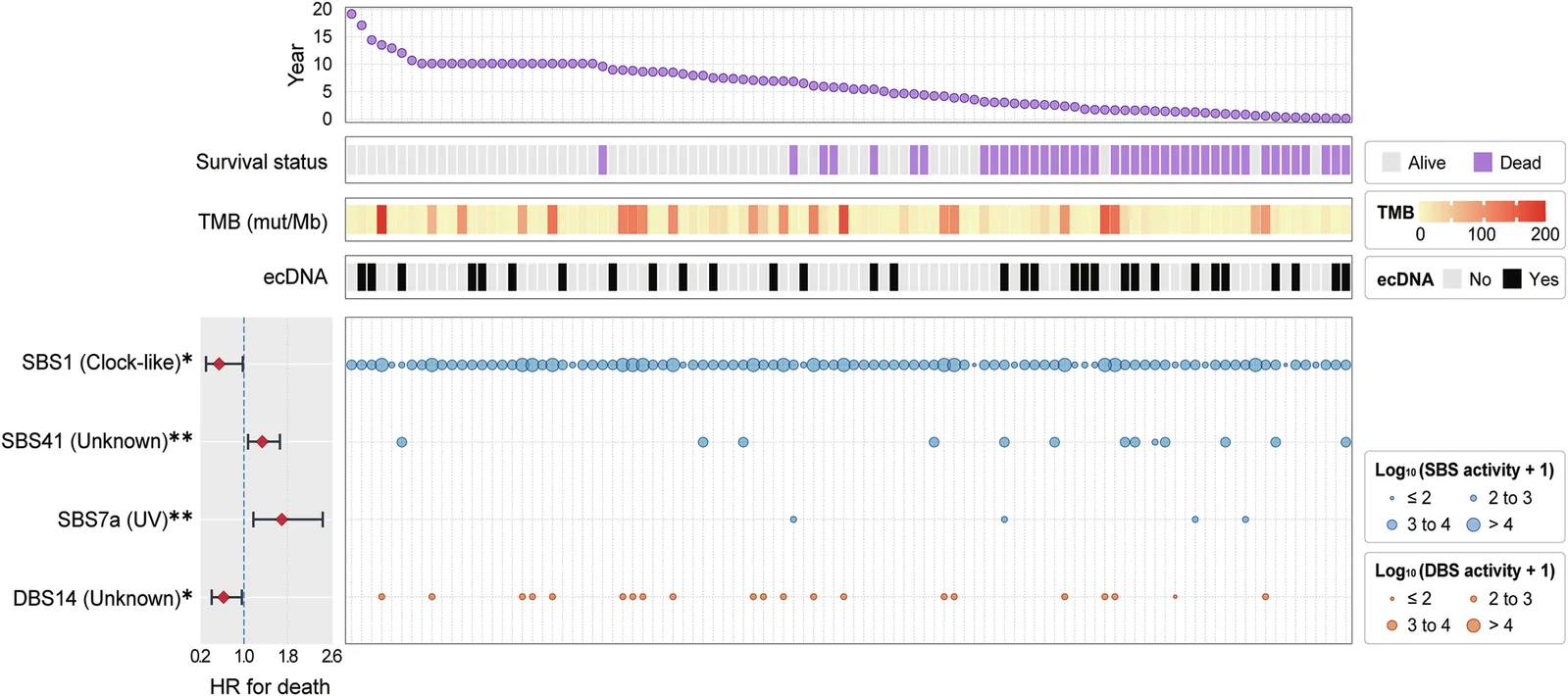
Mutational signatures correlated with overall survival. Gastric cancers are ordered according to decreasing overall survival from left to right. Top panel, follow-up duration of patient, survival status, tumor mutation burden (TMB), and extrachromosomal DNA (ecDNA) status. Bottom panels, log-transformed activity (log_10_ (activity + 1)) of mutational signatures as calculated by SigProfilerAssignment, after excluding mismatch repair and homologous recombination deficiency signatures from microsatellite-stable and homologous recombination proficient samples, respectively. Mutational signatures are sorted according to increasing hazard ratio (HR). Left panel, HR for overall survival. SBS1 (HR = 0.54 (95% CI 0.30–0.98); *P* = 0.041) and DBS14 (HR = 0.62 (95% CI 0.41–0.96); *P* = 0.032) were associated with favorable prognosis, whereas SBS41 (HR = 1.33 (95% CI 1.07–1.66); *P* = 0.009) and SBS7a (HR = 1.69 (95% CI 1.17–2.45); *P* = 0.005) with poor prognosis. *P < 0.05; **P < 0.01. SBS single-base substitution, DBS doublet-base substitution.

In our 100 GCs, we identified a total of 39,316 somatic structural variants (SVs) (15,526 deletions, 12,156 translocations, 6027 duplications, 5319 inversions, and 288 insertions; Fig. 1). The median number of SVs was 231 per tumor (range 2–3,196), and the number of SVs was not significantly associated with prognosis (HR = 1.000 (95% CI 1.000–1.001); *P* = 0.32). Several potentially oncogenic SVs were identified. A GC revealed a translocation between chrX:108,150,467–108,150,468, which is similar to SVs involving the topologically associating domain (chrX:107,720,001–108,600,000) boundary near *IRS4* gene that leads to transcriptional activation in lung squamous cell carcinoma and other tumor types^16^. This SV was associated with *IRS4* mRNA overexpression in the corresponding GC tissue (Supplementary Fig. 5a). Another enhancer hijacking event involving *IGF2* gene (chr11:2,138,686–2,265,093), which was previously reported in colorectal cancer^16^ and GC^17^, was observed in a GC with mRNA overexpression of the cognate gene (Supplementary Fig. 5b). According to mutational signature analyses, SBS1 signature exposure estimated by SigProfilerAssignment^18^, which correlated with TMB (*R* = 0.85; *P* = 2 × 10^-29^), was associated with favorable prognosis (Fig. 2 and Supplementary Table 4b).

**Fig. 3:**
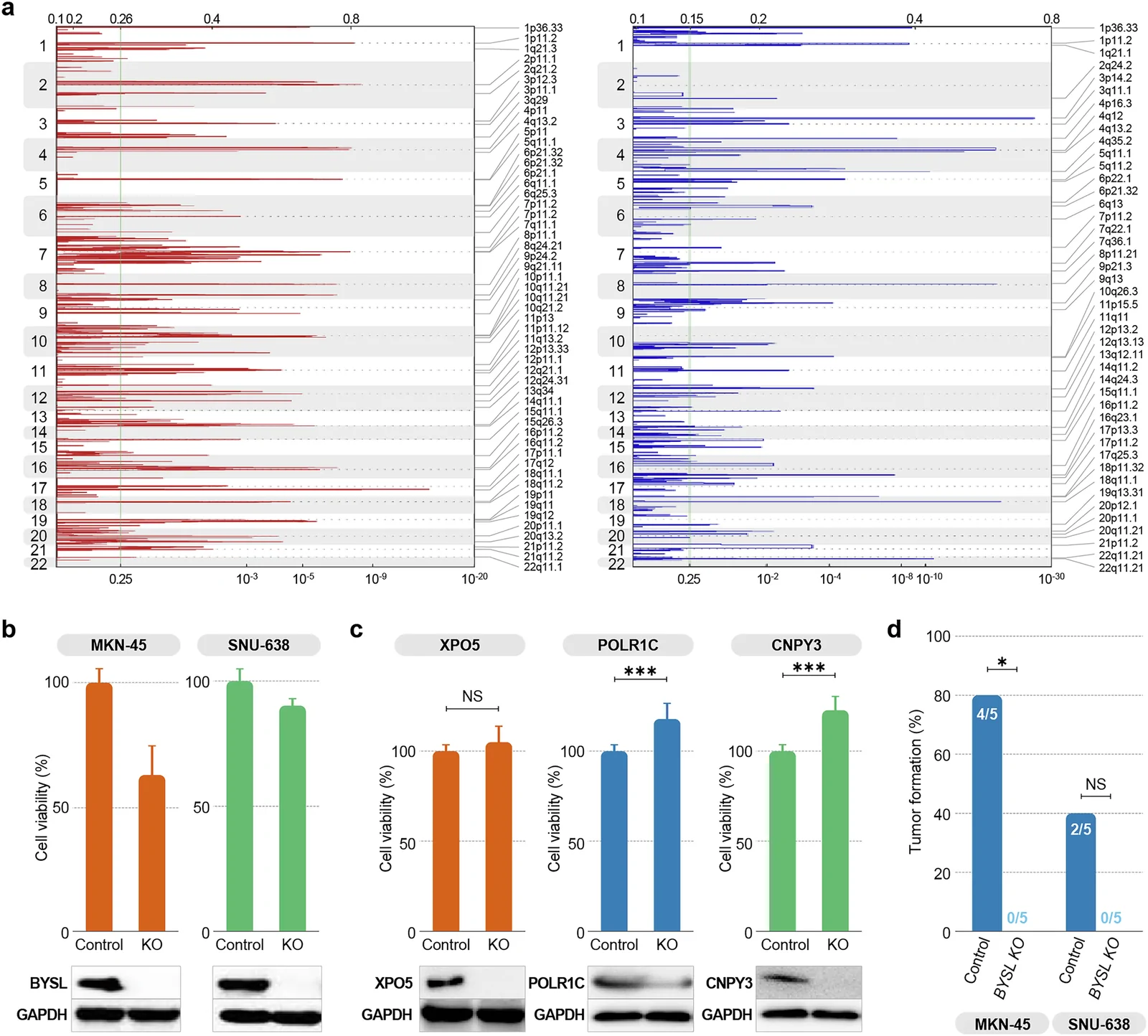
*BYSL* as a novel oncogene candidate in gastric cancer. **a** Amplifications and deletions according to GISTIC analyses. **b** MTT assays after *BYSL* silencing. Significant (*P* < 0.01) suppression of monolayer proliferation of MKN-45 and SNU-638 gastric cancer cells after CRISPR/Cas9 knockout (KO) of *BYSL*. Bottom, Western blot. **c** MTT assays in SNU-638 showing no significant growth inhibition after silencing the other three genes in 6p21 (chr6:41,662,844–43,681,645) locus—*XPO5, POLR1C, and CNPY3—*with significant *cis-* and *trans-*transcriptional activation and protein overexpression in the tumor. **d** In vivo validation. *BYSL*-targeting gRNA-transduced gastric cancer cells showed reduced in vivo tumorigenicity.

According to GISTIC somatic copy number (CN) alteration analyses, there were 20 significantly (*q* < 0.05) recurrent amplified and 21 deleted regions^14^ (Fig. 3a and Supplementary Table 5a and 5b). We then sought to identify novel driver genes located within the 20 recurrent amplicons. By correlating somatic CN alteration data with transcripts per million (TPM) data, we identified 38 genes with significant *cis-* and *trans-*transcriptional activation (*P* < 0.001 and number of significant genes > 500, respectively; Supplementary Table 6a). We next selected four genes—*XPO5*, *BYSL*, *POLR1C*, *and CNPY3—*that were expressed at the protein level in all GCs tested for liquid chromatography–tandem mass spectrometry (LC–MS/MS) analyses and with tumor/normal protein ratio (> 1.2) out of the 29 transcriptionally activated genes in the 6p21 (chr6:41,662,844–43,681,645) locus (Supplementary Table 6b). CRISPR/Cas9 silencing of *BYSL*, which encodes the bystin protein, led to suppression of in vitro GC cell proliferation (Fig. 3b, c). *BYSL* silencing suppressed the in vivo tumorigenicity of these two cell lines when each was injected into the flank subcutaneous tissue of five Balb/c nude mice (*P* for chi-square = 0.01 and 0.11 for MKN-45 and SNU-638, respectively; Fig. 3d). These data are consistent with data in the literature of glioblastoma^19^ and hepatocellular carcinoma^20^. Our 6p21-amplified GCs (*n* = 8) exhibited poorer prognosis than those without (HR = 4.16 (95% CI 1.82–9.50); *P* = 0.0007). Thus, the current study unveiled a previously unrecognized role of 6p21 amplifications in the development and progression of GC and identified *BYSL* as a putative oncogene candidate located in this amplicon for the first time in GC.

### Chromothripsis is a poor prognostic factor in GC

We evaluated different types of chromosomal instability in GC, starting with chromothripsis, a catastrophic event leading to chromosomal instability (Supplementary Table 7). Detection of chromothripsis was primarily based on high-confidence calling per ShatterSeek^5,6^, but we also visually inspected the data to exclude multistep genomic rearrangements. After filtering out three high-confidence ShatterSeek calls by manual curation, we identified a total of 38 chromothripsis events across the 100 GC genomes.

Twenty-two GCs harbored at least one chromothripsis event, and the chromothripsis rate was similar to that of GCs included in the PCAWG consortium study^6^. Age, gender, histology subtype, and primary tumor location were not different between GCs with chromothripsis (*n* = 22) and those without (*n* = 78). Chromothripsis was more frequent in hyperdiploid tumors (39.3%) than in diploid tumors (15.3%; *P* for chi-square = 0.009). The chromothripsis rate was higher in *TP53* mutant tumors (28.2%) than in wild-type tumors (16.7%), but the difference was not significant (*P* for chi-square = 0.16).

**Fig. 4:**
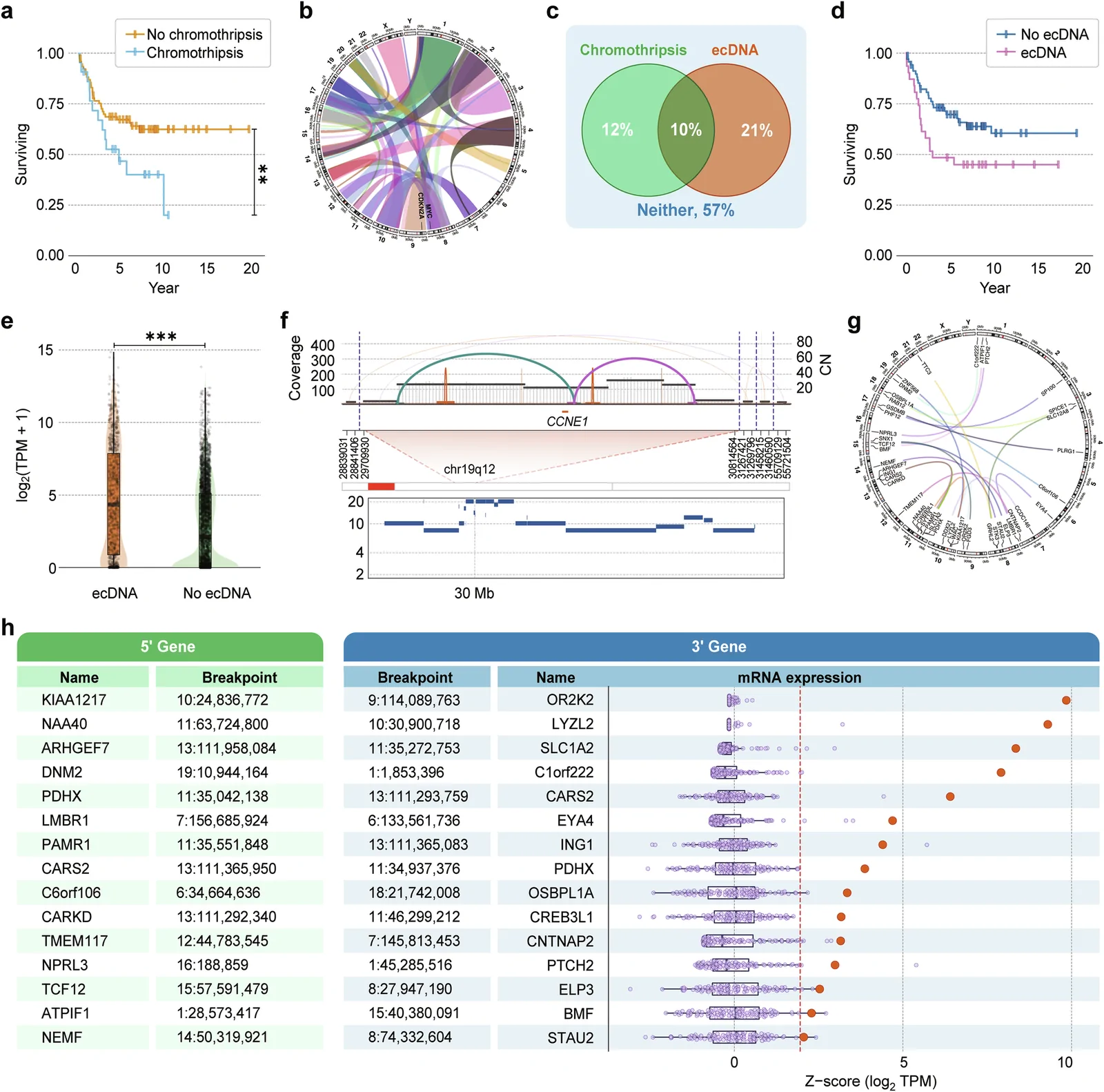
Chromothripsis, extrachromosomal DNA (ecDNA) and in-frame gene fusions. **a** Kaplan–Meier curves for overall survival according to the presence (*n* = 22) or absence (*n* = 78) of chromothripsis (*P* for log-rank test = 0.041). **b** Circos plot of the entire genome for multichromosomal chromothripsis events. **c** Overlap between chromothripsis and circular ecDNA formation among 100 gastric cancers (GCs). **d** Kaplan–Meier curves for overall survival according to the presence (*n* = 31) or absence (*n* = 69) of ecDNA (*P* for log-rank test = 0.050). **e** mRNA expression levels of amplified genes located in ecDNA genomic loci compared with genes not located in ecDNA loci (*P* for *t*-test < 0.001). **f** Example of focal amplification of *CCNE1* localized to a genomic locus that harbored both ecDNA (chr19:29,956,167–30,336,096) and chromothripsis (chr19:28,420,071–56,841,113). **g** Circos plot of in-frame gene fusions (*n* = 25). **h** I*n*-frame gene fusions with overexpression of 3’ partner genes (*n* = 16). Left panel, gene name and chromosomal end of the 5’ partner gene. Right panel, chromosomal start, gene name, and log_2_ (TPM + 1) of the 3’ partner gene of each fusion-positive GC (red circle). Box plot, the remaining fusion-negative GCs (*n* = 99); Vertical red line, *z* = 1.96. CN copy number, TPM transcript per million.

Notably, the number of chromothripsis events in the tumor was associated with poor prognosis (hazard ratio (HR) for death of 1.22 (95% CI 1.00–1.48); *P* = 0.047). Consistent with this finding, 22 GCs that harbored at least one chromothripsis event had worse prognosis than those without chromothripsis (*P* for log-rank = 0.041; Fig. 4a). Median survival times were 4.6 years (95% CI 2.2–not reached) and not reached in those with chromothripsis and those without, respectively.

In 17 patients, 21 of 38 (55.3%) chromothripsis events involved multiple chromosomes. Of the 21 multichromosomal chromothripsis events, 14 (66.7%) involved three chromosomes and 7 (33.3%) involved two chromosomes (Fig. 4b). No predilection of chromothripsis events for a specific chromosome was observed. The risk of death increased by 2.8-fold per multichromosomal chromothripsis event in the tumor (HR = 2.75 (95% CI 1.40–5.42); *P* = 0.003; Supplementary Fig. 18).

We then evaluated if circular extrachromosomal DNA (ecDNA) might be a possible mechanistic link to the poor prognosis associated with chromothripsis, which is a major driver of ecDNA^8,21^. Indeed, GCs with ecDNA tended to exhibit more frequent chromothripsis than GCs without ecDNA (32.3% vs. 17.4%; *P* for chi-square = 0.12; Fig. 4c). Furthermore, ecDNA positivity showed a borderline association with poor prognosis (HR = 1.85 (95% CI 0.99–3.44); *P* for log-rank test = 0.05; Fig. 4d). Focal amplifications localized to the ecDNA loci were transcriptionally activated compared with those not localized to the ecDNA loci (*P* for *t*-test < 0.001; Fig. 4e). Fig. 4f shows an example of focal amplification and mRNA overexpression of *CCNE1* localized to genomic locus involving both chromothripsis and ecDNA. These data suggest that chromothripsis may be poorly prognostic, at least partially, via ecDNA formation.

To evaluate the possible contribution of in-frame fusion to the poor prognosis associated with chromothripsis, we also searched for in-frame fusions in our whole transcriptomic data. A total of 25 in-frame gene fusions that were not previously identified in our own study for diffuse-type GCs^3^ were identified (Fig. 4g and Supplementary Table 12). None of these in-frame gene fusions were recurrent among 100 GCs. Sixteen of 25 in-frame fusions were associated with mRNA overexpression of 3’ partner genes (Fig. 4h). Included among these 16 partner genes were *SLC1A2*, which has been previously reported as a 3’ partner for *CD44–SLC1A2* fusion^22^, and *CREB3L1* that promotes the growth of anaplastic thyroid carcinoma^23^. However, no breakpoints of these 16 fusions were localized to genomic loci harboring chromothripsis.

### Copy number signatures are also associated with prognosis in GC

**Fig. 5:**
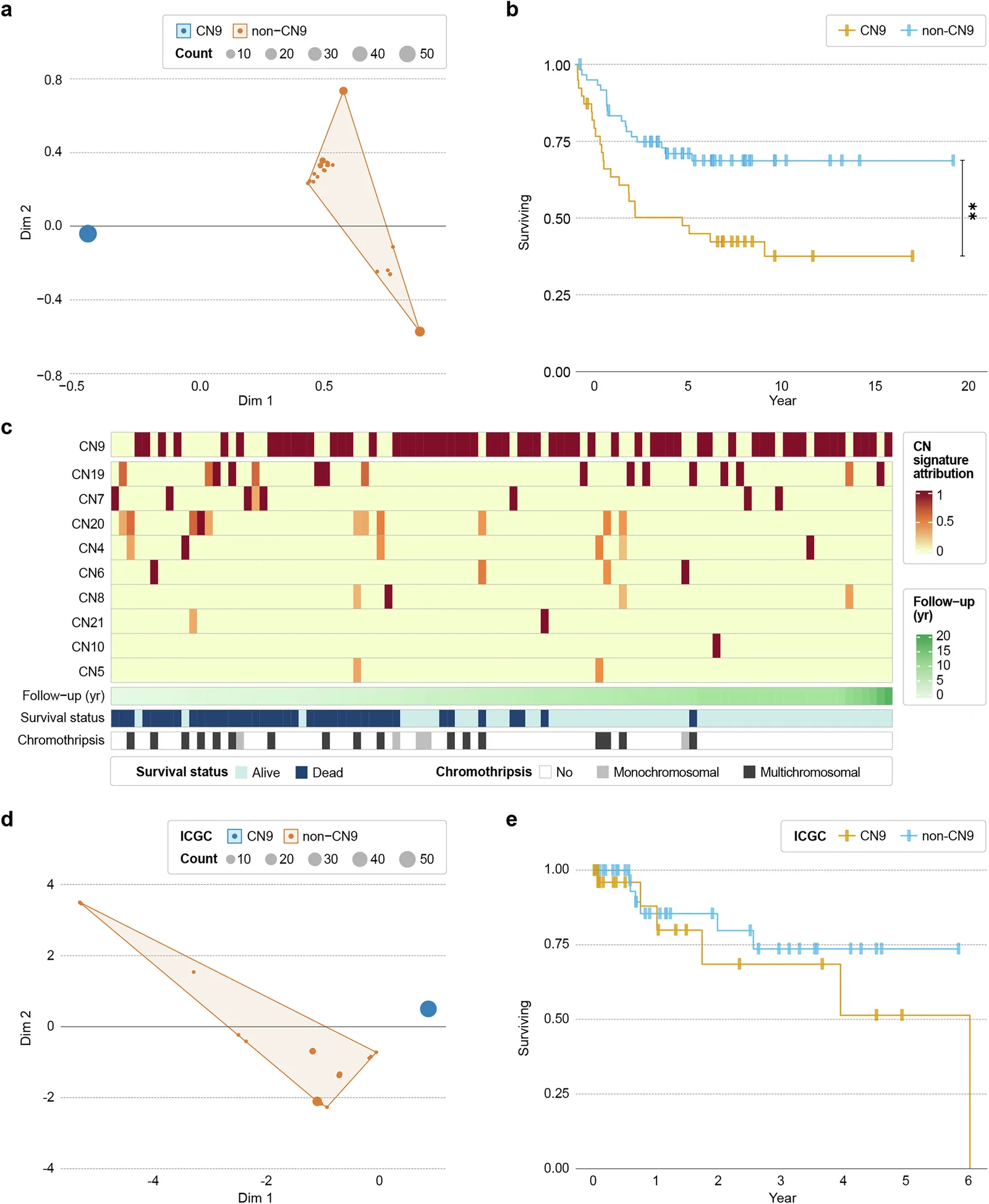
Copy number (CN) signatures. **a** The *k*-means clustering of CN attributions of 100 gastric cancers (GCs). **b** Kaplan–Meier curves for overall survival for CN9 and non-CN9 groups (*P* for log-rank test = 0.003). **c** Top heatmap, Attribution of CN signatures in 100 GCs. Case ordering according to increasing follow-up duration from left to right. **d** The *k*-means clustering of CN attributions of the independent ICGC dataset (*n* = 81). **e** Trend for more favorable overall survival of ICGC dataset (d) associated with CN9 signature (HR = 0.58 (95% CI 0.18–1.90); *P* for log-rank test = 0.36). ICGC International Cancer Genome Consortium.

We then evaluated the performance of simpler methods analyzing chromosomal instability in GC. CN signature, defined by a 48 context CN classification scheme, is based on characteristic patterns of CN changes^24^. No studies have been conducted to test the potential clinical utility of CN signatures in GC. According to the *k*-means clustering of CN signature attributions, 100 GC genomes were divided into two distinct groups (Fig. 5a). One group (*n* = 61) was attributed the CN9 signature, whereas the remaining group (*n* = 39) was attributed CN3, CN4, CN5, CN6, CN7, CN8, CN19, CN20, and CN21 mutational signatures (non-CN9). The two groups differed in the distribution of molecular subtype (*P* for Fisher’s exact test < 0.0001; Supplementary Table 14). CN4, CN5, CN6, and CN20 signatures were more active in chromothripsis-positive tumors than in chromothripsis-negative tumors (Supplementary Table 13).

Notably, the CN9 group (*n* = 61) showed significantly better prognosis than the non-CN9 group (n = 39; *P* for log-rank test = 0.003), with an HR for death of 0.41 (95% CI, 0.22-0.75) (Fig. 5b, c). Median survival time was not reached and 5.5 years (95% CI, 1.8-not reached), in the CN9 and non-CN9 groups, respectively. We also observed a trend for better prognosis with CN9 signature in an independent International Cancer Genome Consortium (ICGC) dataset (*n* = 81; HR = 0.58 (95% CI 0.18–1.90); *P* for log-rank test = 0.36; Fig. 5d, e). These results collectively indicate that CN signature might be used as a simple method to evaluate the prognostic implications of chromosomal instability in GC.

### HRD, present in 4% of GCs, is associated with overexpression of immune-related genes

The exact incidence and genomic causes of HRD have not been fully characterized for GC. Therefore, we evaluated the HRD status of 100 GCs using a random forest-based method—Classifier of HOmologous Recombination Deficiency (CHORD)—which analyzes specific SNV, indel, and SV types identified by WGS^25^, and using HRDetect^26^. When P_HRD_ and HRDetect scores ≥ 0.4 were used to distinguish HRD tumors from homologous recombination proficient (HRP) tumors, 4 of 100 GCs (4%) were defined as HRD.

**Fig. 6:**
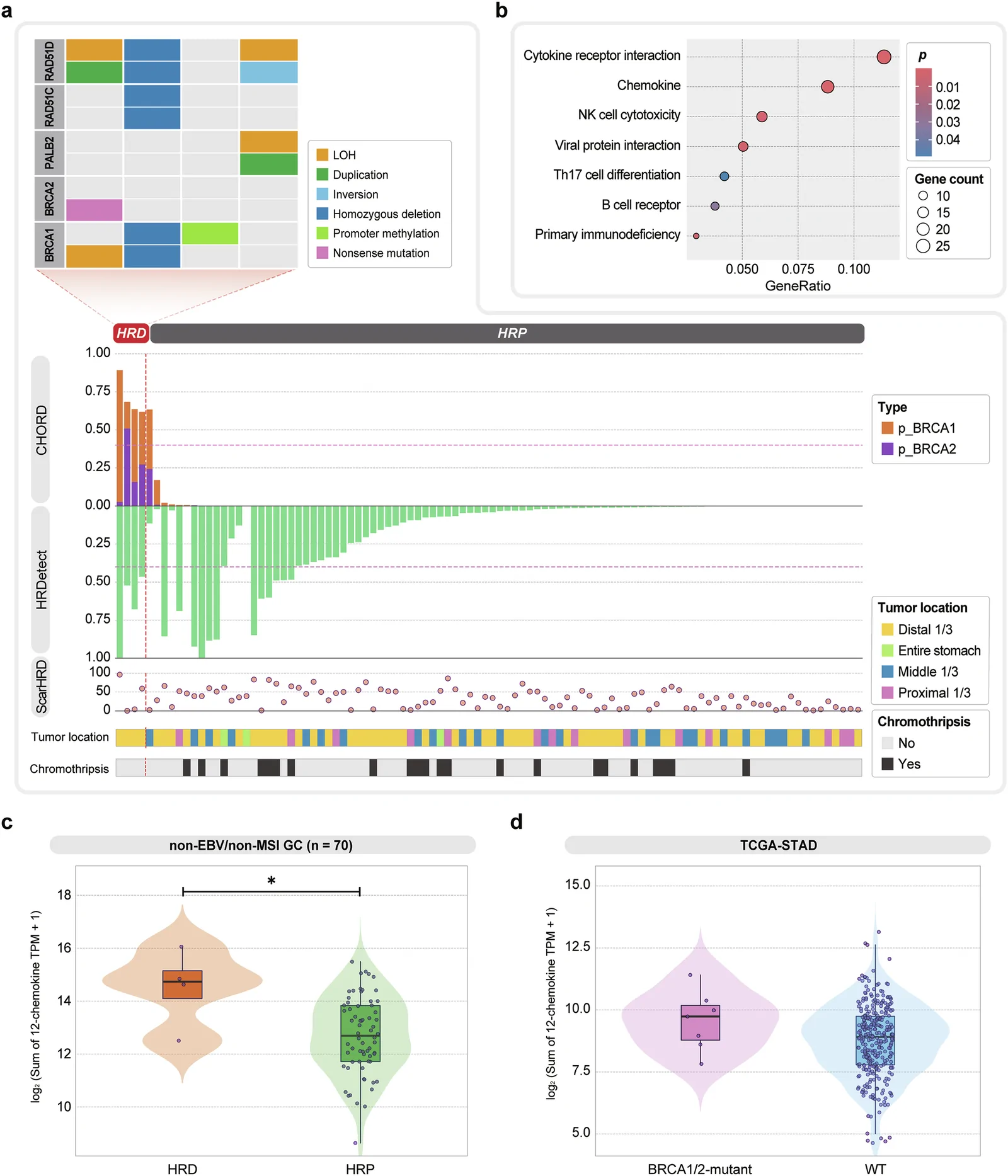
Homologous recombination deficiency (HRD) and 12-chemokine expression. **a** Genomic alterations underlying HRD. Middle panel, CHORD P_HRD_ and HRDetect scores of 100 gastric cancers (GCs). Dotted horizontal red lines indicate cutoffs for defining the HRD tumor (P_HRD_ *and* HRDetect scores ≥ 0.4, upper-left inset). Case ordering according to decreasing P_HRD_ from left to right, for HRD (left of vertical red line) and HRP (right) GCs, respectively. Upper-left inset. HRD GCs (*n* = 4; expanded from the middle panel). The biallelic status of *BRCA1, BRCA2, PALB2, RAD51C*, and *RAD51D* are shown. **b** KEGG pathways significantly enriched in mRNAs that were more highly expressed in HRD GCs (*n* = 4) than in HRP GCs (*n* = 96) (X-axis, FDR in -log_10_ scale). **c** Log_10_-transformed sum of the 12-chemokine expression signature for HRD (*n* = 4) versus non-EBV/non-MSI HRP (*n* = 66) groups in 70 non-EBV/non-MSI GCs (*P* for *t*-test = 0.029). **d** Validation in the TCGA dataset. Log_10_-transformed sum of the 12-chemokine expression signature for *BRCA1/2*-mutant (*n* = 7) versus *BRCA1/2*-wild type (WT), non-EBV/non-MSI TCGA–STAD (*n* = 306) GCs (*P* for *t*-test = 0.15). CHORD Classifier of HOmologous Recombination Deficiency, EBV Epstein–Barr virus, HRP homologous recombination proficient, KEGG Kyoto Encyclopedia of Genes and Genomes, MSI microsatellite instability, TCGA–STAD The Cancer Genome Atlas Stomach Adenocarcinoma.

A tumor with the highest P_HRD_ score (0.89) showed *RAD51D* duplication with LOH, BRCA2 SNV (p.T325Lfs*21), and *BRCA1* LOH. The other three HRD GCs harbored homozygous deletion of *BRCA1/RAD51C/RAD51D*, *BRCA1* promoter methylation, and *PALB2* duplication with LOH combined with *RAD51D* inversion with LOH (Fig. 6a). HRD in the current dataset was attributed entirely by somatic alterations, without any germline mutations in homologous recombination pathways (Supplementary Table 8a).

The anatomic location of all four HRD tumors was the distal third of the stomach (*P* for chi-square = 0.37; Supplementary Table 8a). Age, gender, histology, ploidy, and *TP53* mutation status were not different according to HRD status, and all of the four HRD GCs were negative for Epstein–Barr virus (EBV) and microsatellite instability (MSI). We observed a trend for better prognosis with HRD positivity, but the small number of HRD GCs precluded meaningful survival assessment (Supplementary Fig. 20).

We then compared RNA sequencing data between HRD (*n* = 4) and HRP (*n* = 96) tumors to identify potential therapeutic targets for GC with HRD (Supplementary Table 9). Unexpectedly, the most significantly enriched KEGG pathway in HRD GCs was chemokine signaling (*CCL22/PTK2B/CCL23/CCL11/ADCY8/CCR6/CCL19/TIAM1/ITK/CCL5/GNGT1/CXCR3/CCR7/CX3CR1/DOCK2/CCR2/ADCY5/RASGRP2/PLCG2/WAS/GNG7*), followed by NK (*NCR2/PTK2B/CD247/ARAF/CD48/ITGAL/PTPN6/KLRC4-KLRK1/KLRC3/ZAP70/NCR3/SH2D1A/PLCG2/KLRK1*) and B cell receptor signaling (*CD79A/BANK1/PTPN6/CD79B/BLK/INPP5D/FCGR2B/PLCG2/BTK*) pathways (Fig. 6b)^27^.

When HRD and non-EBV/non-MSI HRP GCs were compared for the sum of 12 chemokines, which reflects intratumoral immune reaction^28^ (Supplementary Fig. 21), the sum was significantly higher in HRD GCs (*n* = 4) than in non-EBV/non-MSI HRP GCs (*n* = 66; *P* for *t*-test = 0.029; Fig. 6c). The sum of 12 chemokines also significantly correlated with the P_HRD_ of 70 non-EBV/non-MSI GCs (*R* = 0.35, *P* = 0.003). Other than P_HRD_, only the coding indel/SNV ratio significantly correlated with the summed RNA expression levels of the 12 chemokines (*R* = 0.44, *P* = 0.0001). To obtain possible mechanistic insights into the chemokine overexpression, we compared TMB and neoantigen load between our HRD (*n* = 4) and HRP, non-EBV/non-MSI GCs (*n* = 66). There was no difference in TMB and neoantigen load between the two groups (Supplementary Table 18). Therefore, overexpression of 12 chemokines in HRD GCs is unlikely to be due to the overproduction of HRD-induced neoantigens. These data collectively propose a hypothesis that unrepaired DNA lesions might have activated innate immunity of HRD GCs without forming neoantigen^29–33^. When our finding of chemokine overexpression in HRD GCs was tested in an independent dataset, the sum of 12 chemokines tended to be higher in BRCA1/2-mutant TCGA–STAD GCs than in wild-type GCs (*P* for *t*-test = 0.15; Fig. 6d).

### Somatic retrotransposition events are most strongly associated with global hypomethylation in the tumor

**Fig. 7:**
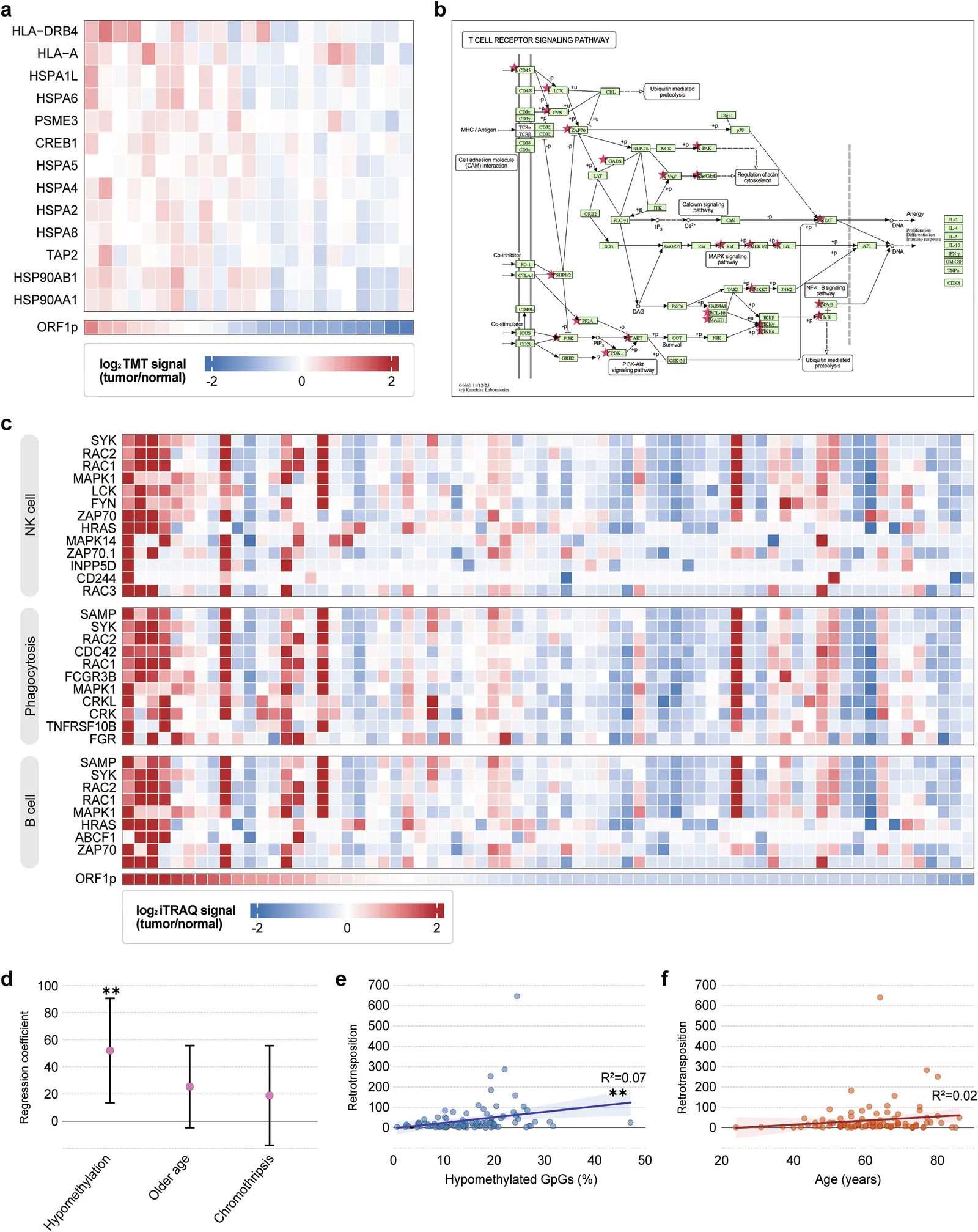
Retrotransposition. **a** Liquid chromatography–tandem mass spectroemetry global proteomic profiling data of a subset of 23 non-EBV/non-MSI gastric cancers (GCs) of the 100 patients in the main study population. Top panel, Heatmap for expression levels of antigen processing and presentation pathway proteins across 23 non-EBV/non-MSI GCs. Red and blue colors representing high and low protein expression levels, respectively. Data after median protein centering. Bottom panel, ORF1p protein expression level in the tumor. Case ordering according to decreasing ORF1p protein expression. **b**, **c** Proteome data in an independent set of 70 early-onset GCs. **b** T cell receptor KEGG pathway enriched in ORF1p-correlated proteins (shown as red stars) among an independent set of 70 early-onset GCs. **c** Heatmap for ORF1p-correlated KEGG pathways among an independent set of 70 early-onset GCs. Upper panel, Natural killer cell-mediated cytotoxicity. Middle panel, Fc gamma R-mediated phagocytosis. Bottom panel, B cell receptor pathways. **d, e** Whole genome sequencing data (*n* = 100). **d** Retrotransposition events were best modeled by hypomethylation (> 20%), older age (> 62 years), and the presence of chromothripsis. Dots, coefficients of linear regression (whiskers, 95% CI) (adjusted *P* values = 0.008, 0.09, and 0.28, respectively). **e** Correlation between the number of coverage-adjusted genomic retrotransposition events and the degree of hypomethylation in the tumor (*R* = 0.27; *P* = 0.008). **f** Correlation with patient age (*R* = 0.15; *P* = 0.14). EBV Epstein–Barr virus, KEGG Kyoto Encyclopedia of Genes and Genomes, MSI microsatellite instability.

Given that immune checkpoint blockade is efficacious only in GC with immune activation^34^, we further explored gene expression correlates of genomic instability beyond HRD. Retrotransposition can generate DNA double-strand breaks that may contribute to chromosome fragmentation^35,36^. We therefore evaluated retrotransposition-associated expression profiles in our GCs using ORF1p protein expression level as a surrogate biomarker for its activity. According to LC–MS/MS global proteomic profiling analyses of a subset of our non-EBV/non-MSI GCs (*n* = 23), protein expression level of ORF1p positively correlated with those of 701 proteins (*R* > 0.413; *P* < 0.05) (Fig. 7a and Supplementary Table 10a). Supplementary Table 27 summarizes KEGG pathways enriched in 701 proteins significantly (FDR < 0.05) correlating with ORF1p in protein expression profiles. In addition to the spliceosome pathway, nucleocytoplasmic transport, mRNA surveillance, DNA replication, and repair, and antigen processing and presentation pathways were enriched in 701 proteins whose protein expression levels correlated with that of ORF1p. Included in antigen processing and presentation pathway were *HSPA2*, *HSP90AA1*, *HLA-A*, *HSPA1L*, *HSPA4*, *HLA-DRB4*, *HSPA5*, *HSPA8*, *CREB1*, *HSPA6*, *PSME3*, *TAP2*, and *HSP90AB1* (Fig. 7a). cGAS protein expression level also tended to positively correlate with ORF1p expression level (*R* = 0.34; *P* = 0.11). These data could raise a hypothesis that ORF1p-activated immunogenic cytosolic nucleic acids eventually contributed to the overexpression of antigen processing and presentation pathway proteins.

We wished to validate this interesting finding in independent datasets, but there were no publicly available proteomic datasets for GC tissue samples other than our prior study^37^. Therefore, we had to reanalyze our own prior published data for validation. In 70 early-onset GCs that were negative for EBV and MSI, the nucleocytoplasmic transport and DNA replication and repair pathways were again enriched among 3,140 proteins correlated with ORF1p in protein expression ratio between tumor and adjacent normal tissue of the same patient (*R* > 0.2355; *P* < 0.05; Supplementary Table 28). Notably, T cell receptor pathway was also enriched in these ORF1p-correlated proteins (Fig. 7b). When we focused on 476 proteins with the most significant ORF1p correlations (*R* > 0.405; *P* < 0.005) among the same set of 70 non-EBV/non-MSI early-onset GCs, natural killer cell-mediated cytotoxicity, Fc gamma R-mediated phagocytosis, Fc epsilon RI signaling, and B cell receptor pathways were included in 15 significant KEGG pathways (Fig. 7c).

### Global hypomethylation is associated with genomic retrotransposition events

Across our 100 paired genomes, a total of 3,965 somatic retrotransposition events occurred (median 16 per tumor; range 0–773), with LINE-1 representing the majority of somatic retrotranspositions (87%), according to TraFiC-mem analyses^38^. The number of somatic retrotransposition events was not associated with the overall survival of our cohort of 100 patients with GC. Genomic retrotransposition events were more frequent in GCs with global hypomethylation (*n* = 21), which was defined by ≥ 20% CpGs being hypomethylated in the tumor compared with normal (Δβ < −0.2), than in GCs without hypomethylation (*n* = 79; Fig. 7d,e). According to the Elastic Net algorithm^39^, the number of coverage-normalized retrotransposition events was best modeled as follows:

$${\mathrm{Retrotransposition}}\,=\,61.63\,\left({\mathrm{global}}\,{\mathrm{hypomethylation}}\right)\,+\,32.18\,\left({\mathrm{age}}\, > \,62\right)\,+\,20.37\,\left({\mathrm{chromothripsis}}\right)\,+\,3.71$$

where each variable was coded as 1 if present and 0 otherwise.

Although patient age and global hypomethylation in the tumor are associated with retrotransposition^40,41^, to our knowledge, the current paper is the first to present a comprehensive regression model for retrotransposition in GC (Fig. 7d). Moreover, these results provide additional evidence for a possible contribution of retrotransposition to chromothripsis in solid tumors.

**Fig. 8:**
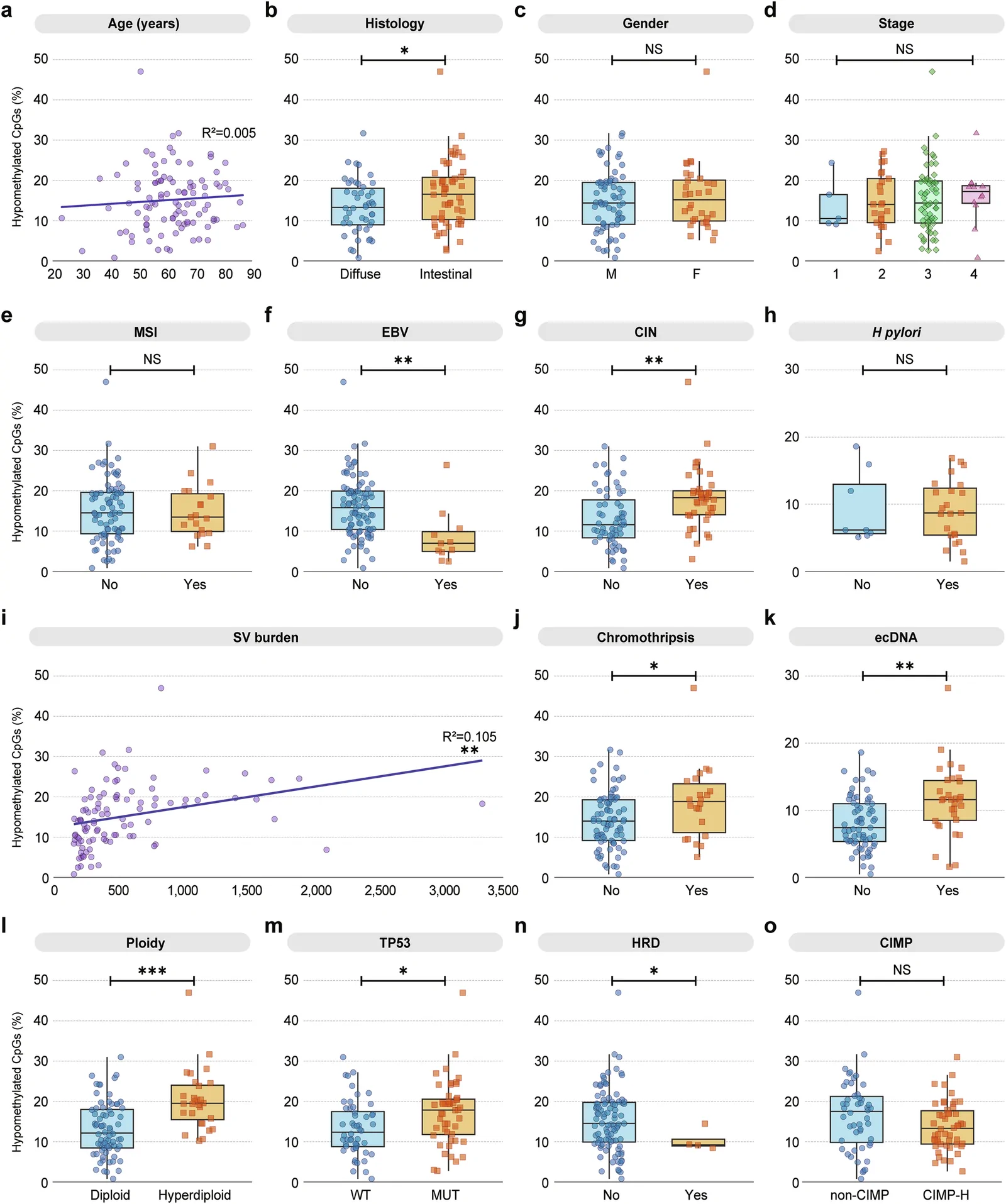
Global hypomethylation. Percentage of hypomethylated (β_tumor_ − β_normal_ (Δβ) < −0.2) CpGs in the tumor according to: **a** Age (*P* for Pearson = 0.48); **b** Histology (*P* for *t*-test = 0.03); **c** Gender (*P* = 0.51); **d** TNM stage (*P* = 0.94); **e** Microsatellite instability (MSI; *P* = 0.86); **f** Epstein–Barr virus (EBV; *P* = 0.005); **g** Chromosomal instability (CIN; *P* = 0.001); **h** *Helicobacter pylori* (*H. pylori;* *P* = 0.78); **i** Structural variant (SV) count (*P* for Pearson = 0.008); **j** Chromothripsis (*P* = 0.04); **k** Extrachromosomal DNA (ecDNA; *P* = 0.003); **l** Ploidy (*P* < 0.001); **m** *TP53* mutation (*P* = 0.01); **n** Homologous recombination deficiency (HRD; *P* = 0.02); **o** CpG island methylator phenotype (CIMP; *P* = 0.08). Box, interquartile range (IQR). Bar, median; *P < 0.05; **P < 0.01; ***P < 0.001. NS not significant.

Finally, these results led us to explore clinicopathological and genomic correlates of global hypomethylation in our GCs (Fig. 8). Unlike retrotransposition, global hypomethylation itself showed no tendency to be associated with patient age (Figs. 7f and 8a). As previously reported^42^, hypomethylation was more prominent in intestinal-type GC. *Helicobacter pylori* status was not associated with the degree of hypomethylation. Notably, global hypomethylation was more prominent in GCs with higher burden of SVs, chromothripsis, ecDNA, hyperdiploidy, and *TP53* mutation. Thus, our study is the first comprehensive, quantitative analysis of global hypomethylation in relation to specific types of chromosomal instability in GC.

## Discussion

To our knowledge, our study is the first to identify *BYSL* as a novel potential driver oncogene within the recurrent 6p21 amplicon in GC. Target oncogenes within 6p21 amplification are largely unexplored, especially in GC. For B cell malignancies, where the 6p21 locus is frequently involved in amplification and translocation, *CCND3* and *BYSL* were proposed as the most likely driver oncogenes at this locus^43^. Oncogenic roles of *BYSL* have been previously documented in other solid tumors^19,20^, but not in GC. Bystin, the *BYSL* gene product, plays a role in nucleologenesis during cell proliferation^20^ and is a potential cancer immunotherapy target given restricted expression in normal tissue^44^. Of note, the 6p21 amplicon (chr6:41662844–43681645) harboring *BYSL* was associated with poor prognosis among our 100 GCs. Thus, the current study is the first to unveil the prognostic and carcinogenic role of *BYSL* in GC.

WGS analyses can evaluate mutational signatures more accurately than whole exome sequencing. Thus far, only a few WGS studies have been conducted for GC and are limited by short follow-up duration (approximate median 39 months in a study by Xing, et al.^2^). Therefore, specific events possibly leading to chromosomal instability, such as retrotransposition, have not been adequately evaluated in GC. Moreover, combined WGS and proteomic profiling studies have not been conducted in GC.

Chromothripsis is reportedly associated with poor prognosis in many types of cancer including myeloma, medulloblastoma, triple-negative breast cancer, and colorectal cancer, but not in GC. Chromothripsis drives ecDNA amplification into circular double minutes^29,45,46^. Extrachromosomal *MYC* amplification in double minutes has been described in acute meyloid leukemia and small cell lung cancer^10,45^. Our study is the first to demonstrate that the number of chromothripsis events per tumor is significantly associated with increased risk of death, with the risks associated with multichromosomal chromothripsis being even higher. These data, if validated in larger cohorts, are clinically relevant, as WGS data will be more readily available for postoperative prognostication.

CN signatures have just started to be investigated^24^, and poor prognostic CN signatures have been reported in ovarian and prostate cancers^47,48^. Of great clinical interest, the current study is the first to suggest that CN signature analysis may be used for prognostication given the prominent poor prognostic impact of non-CN9 signatures in GC. Of the non-CN9 signatures, CN4, CN5, and CN6 signatures have been linked to chromothripsis^48^, and our data is consistent with these reports (Supplementary Table 13). Given that CN signature analysis can be amenable to a simpler and less expensive analytic platform, further studies are warranted to validate its possible clinical utility.

HRD in GC has not yet been fully studied, especially using WGS analysis. CHORD, a WGS-based algorithm used in the current study, calls HRD, based on SNVs, indels, and SVs identified in tumors with biallelic inactivation of *BRCA1* (*n* = 35) and *BRCA2* (*n* = 89)^25^, and has higher specificity than any other current algorithms to our knowledge. However, a relatively small number of GCs were evaluated in a prior CHORD study^25^. Our HRD analysis demonstrated a clear bimodal distribution between HRD (4%) and HRP (96%), and provides the first comprehensive WGS-based estimate of HRD frequency in GC. In addition to EBV and MSI, CIN-hot GCs have been described as relatively rich in T cells^49^. Our data extend the literature by highlighting HRD as a potential contributor to immune activation, which can be validated by future WGS-based clinical trials of PARP inhibitors in combination with immune checkpoint inhibitors. This hypothesis is of clinical interest given the relative paucity of molecularly targeted agents approved in GC.

Our work is the first to present a model of genomic retrotransposition in GC, highlighting the association with global hypomethylation. We hypothesize that global hypomethylation activates LINE-1 transcription, whose endonuclease activity could contribute to chromosome shattering^35,50^. Moreover, our study is the first comprehensive, quantitative analysis of global hypomethylation in relation to specific forms of chromosomal instability in GC.

Taken together, our findings provide novel insights into the dysregulation of DNA stability and repair and their clinical relevance in human GCs.

## Supplementary information

Supplementary Information

Supplementary fTable 1

Supplementary Table 2

Supplementary Table 3

Supplementary Table 4

Supplementary Table 5

Supplementary Table 6

Supplementary Table 7

Supplementary Table 8

Supplementary Table 9

Supplementary Table 10

Supplementary Table 11

## Data Availability

Sequencing data are deposited in the CODA (Clinical & Omics Data Archive) (https://coda.nih.go.kr/usab/pheweb/intro.do) with the accession (Accession number: CODA_D26053).
