# Supplementary material for: Immune pathway activation in gastric cancers with LINE-1 retrotransposon overexpression and homologous recombination deficiency
Source: Exp Mol Med. 2026 Sep 7;58(9):3061–73. doi: 10.1038/s12276-026-01846-5 (PMC13633253; doi:10.1038/s12276-026-01846-5)
Supplement: Supplementary file 1 [file 12276_2026_1846_MOESM1_ESM.pdf]

Immune pathway activation in gastric cancers with LINE-1 retrotransposon overexpression and homologous recombination deficiency

Supplementary Information

Supplementary Tables (Excel Files)

Supplementary Table 1a. Overview of the datasets used in this study and their corresponding figures and tables
Supplementary Table 1b. Clinicopathological characteristics of main study population (*n* = 100)
Supplementary Table 1c. Summary of sequencing metrics for whole genome, RNA, DNA methylation
Supplementary Table 2. Mutect2 results
Supplementary Table 3. MutSig2CV result for recurrent SNVs
Supplementary Table 4a. Prognostic association of SBS, DBS, ID and SV
Supplementary Table 4b. Prognostic association of SBS, DBS, and ID after refitting
Supplementary Table 5a. GISTIC2: significant amplification regions
Supplementary Table 5b. GISTIC2: significant deletion regions
Supplementary Table 6a. Genes with *cis-* and *trans*-transcriptional activation
Supplementary Table 6b. Genes with *cis*- and *trans*-transcriptional activation that are expressed in protein level across all GCs (Genes for functional study are shown in yellow)
Supplementary Table 7. Chromothripsis events
Supplementary Table 8a. Genetic causes of HRD tumors
Supplementary Table 8b. HRD profiles of 100 GCs
Supplementary Table 9. Summary of RNAseq data for genes overexpressed in 4 HRD compared with 96 HRP GCs
Supplementary Table 10a. Summary of global proteomic profiling data for ORF1p-correlated proteins in a subset of 23 non-EBV/non-MSI GCs
Supplementary Table 10b. iTRAQ signal intensity ratio between tumor and normal for ORF1p-correlated proteins in an independent dataset from 70 non-EBV/non-MSI early-onset GCs
Supplementary Table 11a. Somatic retrotransposition events
Supplementary Table 11b. Hypomethylation and associated genomic features

Table of Contents

Patients 5
Whole-genome sequencing analyses 6
– Processing 6
– The distribution of whole-genome sequencing coverage 6
– Somatic nucleotide variation (SNV) and indel 7
– Prognostic association of *TP53* mutation 7
– Somatic copy number alteration 8
– Hyperdiploidy and survival outcomes 9
– Structural variation (SV) 9
RNA-seq analyses 10
– Processing 10
– Genes with *cis*- and *trans*-activation 10
– *TERT* promoter mutation with transcriptional activation 11
– Genomic rearrangements with transcriptional activation 11
– Epstein-Barr virus (EBV) status 12
– Gene fusion 12
– 3' gene overexpression and fusion breakpoints 14
Methylation sequencing analyses 15
– Processing 15
– Global methylation in the tumor 15
– CpG island methylator phenotype (CIMP) 15
COSMIC mutational signature 17
– SBS, DBS, ID, and SV mutational signatures 17
– Mutational signature refitting and survival analysis 17
– MSI mutational signature 17
– CN mutational signature 18
– Microenvironment analysis by CN9 status 19
Tumor mutation burden (TMB) 22
Microsatellite instability (MSI) 24

TCGA subgroup *26*

H. pylori status *27*

Chromothripsis *28*

Extrachromosomal circular DNA (ecDNA) *31*

Homologous recombination deficiency (HRD) *32*

– HRD Status 32

– CHORD 32

– HRDetect 32

– Germline mutation contributed to HRD 33

– HR-related gene alterations and methylation status 33

– Neoantigen load (NAL) and TMB in HRD vs HRP 34

– RNA-seq expression profiles of HRD 35

– 12-chemokine gene expression 35

Genomic retrotransposition events *38*

CRISPR-Cas9 knockout and MTT assay *41*

In vivo tumorigenicity *42*

Co-occurrence and mutual exclusivity of BYSL amplification *43*

Global proteomic profiling analyses of 23 non-EBV/non-MSI GCs *44*

– Methods 44

– Results 47

Global proteomic profiling analyses of an independent dataset of 70 non-EBV/non-MSI early-onset GCs[48] *50*

Supplementary references *55*

Patients

We conducted whole genome, bulk RNA, and methylation sequencing analyses with 100 resected tumors and matched germline samples that were collected from Korean patients with GC. After light microscopic examination of the top slides, tumor-rich areas were macrodissected from the frozen tumor specimens for the isolation of genomic DNA and total RNA. Adjacent normal tissues were collected from the luminal side of the gastrectomy specimen at least 2 cm from the tumor border. Protein lysate was isolated from the same tissue powder that was used to isolate genomic DNA and RNA. Tumor tissue DNA was extracted from macrodissected tissue samples using the QuickGene DNA tissue kit S with QG-Mini80 instrument (Kurabo, Osaka, Japan) following the manufacturer's instructions. Total RNA was isolated from the same piece of the tumor that was used for DNA isolation and from adjacent normal tissue samples using a mirVana kit (Thermo Fisher Scientific, Waltham, MA).

Fresh-frozen gastric adenocarcinomas and matched adjacent normal tissue samples were obtained from 60 and 40 Korean patients with gastric cancer (GC) at Seoul National University Hospital (SNUH) (Seoul, Korea) and National Cancer Center (NCC) Hospital (Goyang, Korea), respectively. This study was approved by the SNUH and NCC Institutional Review Boards (SNUH C-1402-054-555 and NCC2017-0238).

Among 100 GCs, there were 68 males, 54 intestinal types, and 58 distal tumors. Most (88%) patients underwent curative resection with 5, 25, and 58 being resected at American Joint Committee on Cancer (AJCC) 8th Edition stage 1 (IB), 2 (IIA (n = 6)/IIB (n = 19)), and 3 (IIIA (n = 33)/IIIB (n = 11)/IIIC (n = 14)), respectively (Supplementary Table 1b).

Survival analyses for overall survival and relapse-free survival were conducted using proc lifetest and proc phreg of SAS v9.4 (SAS Institute, Cary, NC, Statistical Analysis System).

Whole-genome sequencing analyses

DNA library was prepared using the TruSeq DNA PCR-Free Library prep kit (Illumina, San Diego, CA) following the manufacturer's instructions. For sample library preparation, 2 μg of insert 550 bp size of high molecular weight genomic DNA was randomly sheared to yield DNA fragments using the Covaris S2 system. The fragments were blunt-ended and phosphorylated, and a single 'A' nucleotide was added to the 3' ends of the fragments in preparation for ligation to an adapter that has a single-base 'T' overhang. Adapter ligation at both ends of the genomic DNA fragment conferred different sequences at the 5' and 3' ends of each strand in the genomic fragment. The quality of the libraries was verified by capillary electrophoresis (Bioanalyzer, Agilent Technologies, Palo Alto, CA). For sequencing, library concentration was assessed using a Qubit 2.0 Fluorometer and dsDNA HS Assay Kit (Thermo Fisher Scientific, MA) according to the manufacturer's protocol. After quantification using QX100 Droplet Digital PCR System (Bio-Rad, Hercules, CA), we combined index-tagged libraries in equimolar amounts in the pool. Sequencing was performed using an Illumina NovaSeq 6000 system (Illumina, San Diego, CA) following provided protocols for 2 x 151 bp sequencing. Library preparation and sequencing were performed by DNA Link (DNA Link Inc., Seoul, Korea).

– Processing

We used BWA v0.7.15-r1140[1] to align the 151-bp paired-end whole-genome sequencing reads to human reference genome G1Kv37 (hg19). Picard v1.128 (https://github.com/broadinstitute/picard) was used for duplicate marking, and SAMtools v1.7[2] was also used for basic processing and management of the sequencing data.

– The distribution of whole-genome sequencing coverage

Mean coverages were 29.6 and 47.0 for normal and tumor samples, respectively (Supplementary Fig. 1).

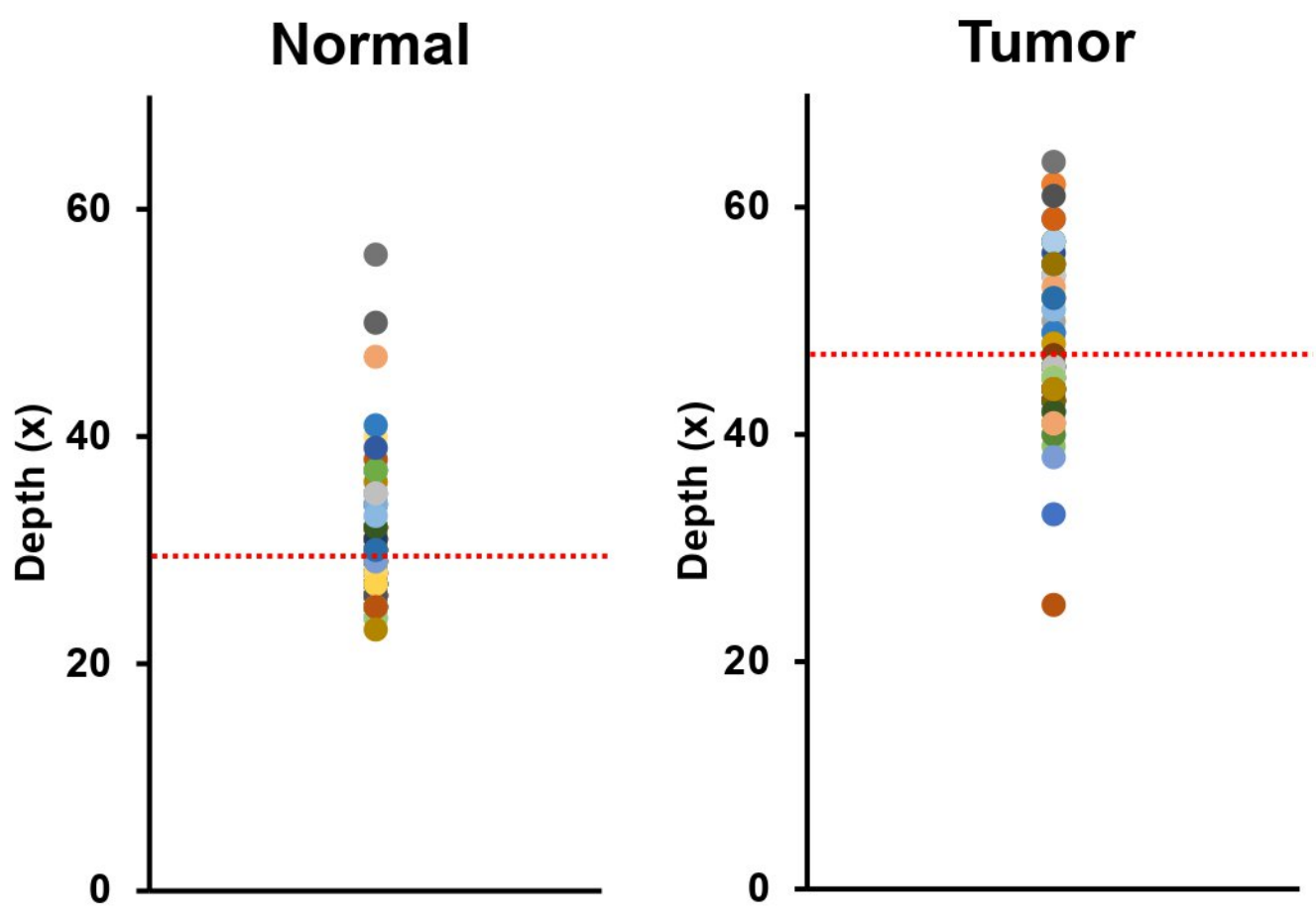

Supplementary Fig. 1. Whole-genome sequencing depth of 100 gastric cancers

- **Somatic nucleotide variation (SNV) and indel**

To identify recurrent somatic nucleotide variations (SNVs) and indels, GATK Mutect2 [3] and MutSig2CV v3.11[4] were used at default settings. LearnReadOrientationModel of GATK was used to assess read orientation bias. GetPileupSummaries and CalculateContamination were used to filter somatic mutations using FilterMutectCalls. The somatic variants that passed all filters were annotated using VEP v102[5] and using vcf2maf v1.6.19 (https://zenodo.org/record/1185418) to convert the VCF file to Mutation Annotation Format (MAF) file (Supplementary Table 2 and 3).

- **Prognostic association of *TP53* mutation**

TP53 mutation was observed in 48 patients and tended to be associated with poor prognosis (*P* for log-rank = 0.24; HR = 1.45 (95% CI, 0.78-2.69); *P* = 0.24). Median survival times were 9.62 years (95% CI, 3.04–not reached) and not reached (95% CI, 4.58–not reached), in patients with mutant and wild-type *TP53* tumors, respectively (Supplementary Fig. 2)

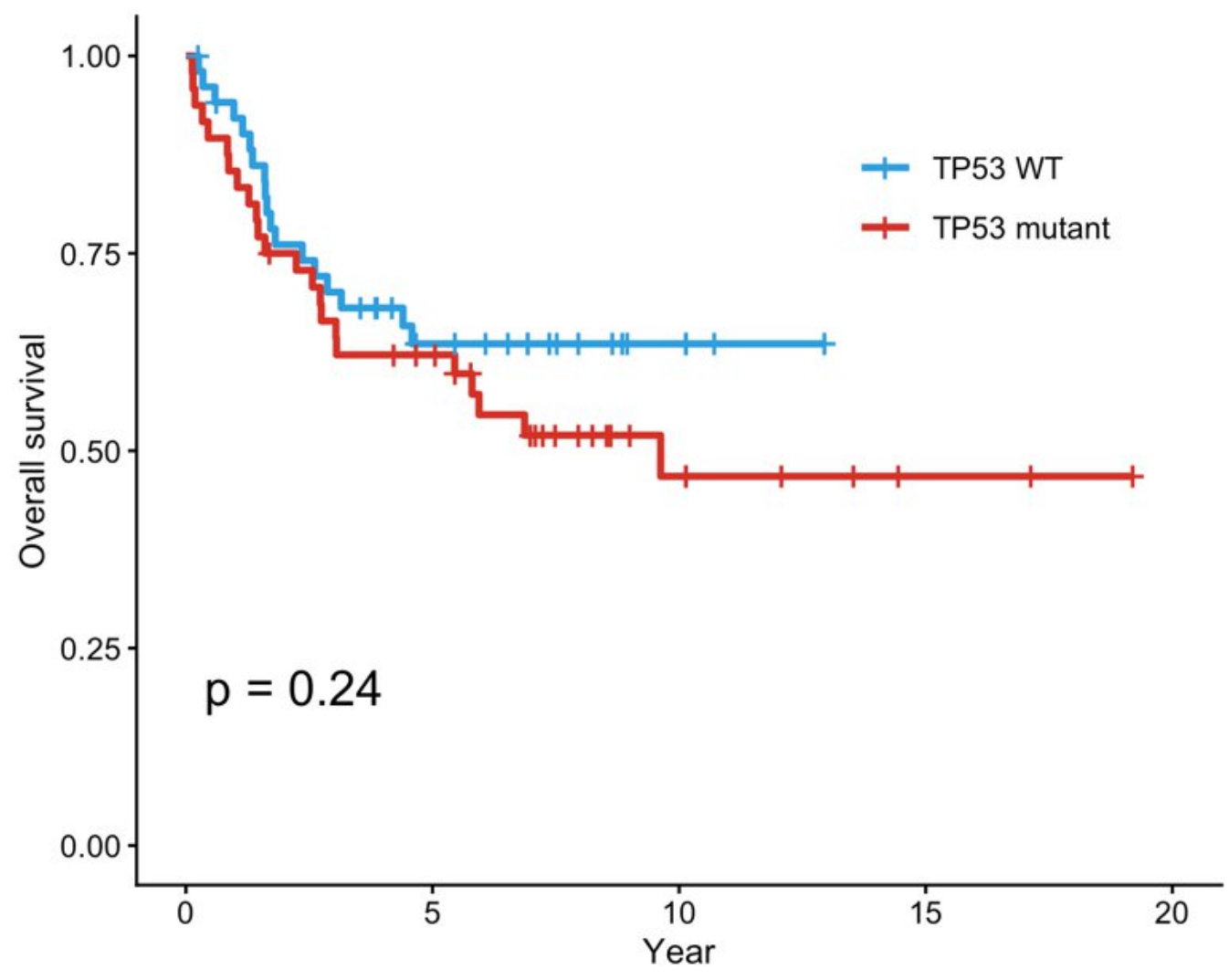

Supplementary Fig. 2. Kaplan-Meier curves for overall survival according to the TP53 mutation

– Somatic copy number alteration

We used the Sequenza v3.0.0,[6] and R package with whole-genome sequencing, to estimate tumor purity, ploidy, and allele-specific copy number variations (CNVs). GC content within sliding windows was calculated using sequenza-utils and the hg19 (G1Kv37) human reference genome. Data from normal and tumor BAM files were processed, identifying genomic positions with sufficient sequencing depth (by default, >20) and determining variant alleles and allelic frequencies. The Sequenza R package was used for GC normalization, allele-specific segmentation, and Bayesian inference to deduce purity and ploidy parameters, generating copy number profiles. Loss of heterozygosity (LOH) was called when the B allele frequency was zero. GISTIC2 v2.0.23[7] was used to detect genomic regions exhibiting a statistically significant increase in copy number aberrations compared to the baseline (Supplementary Table 5a and 5b).

Twenty significant (q < 0.05) amplification regions (Supplementary Table 5a) of our cohort were classified as 4 categories; (1) chromosomal instability with amplification, (2) chromosomal instability without amplification, (3) amplification without chromosomal instability, and (4) no amplification or chromosomal instability. Amplification threshold of the GISTIC2 result was T > 1.3. Similarly, 21 significant (q < 0.05) deletion regions (Supplementary Table 5b) of our cohort were classified as 4 categories; 1. chromosomal instability with deletion (value ≤ -1 per GISTIC2), 2. chromosomal instability without deletion, 3. deletion without chromosomal instability, and 4. no deletion or chromosomal instability. Deletion threshold of the GISTIC2 result was T < -0.1.

Fisher's exact test was then conducted to determine the significance of overlap between specific chromosomal instability type and significant GISTIC2 region.

– Hyperdiploidy and survival outcomes

Hyperdiploidy (ploidy ≥ 3; *n* = 28) was associated with poor prognosis (*P* for log-rank = 0.002). Median survival was 2.73 years (95% CI, 1.43–not reached) in patients with hyperdiploidy and was not reached in those without hyperdiploidy (Supplementary Fig. 3).

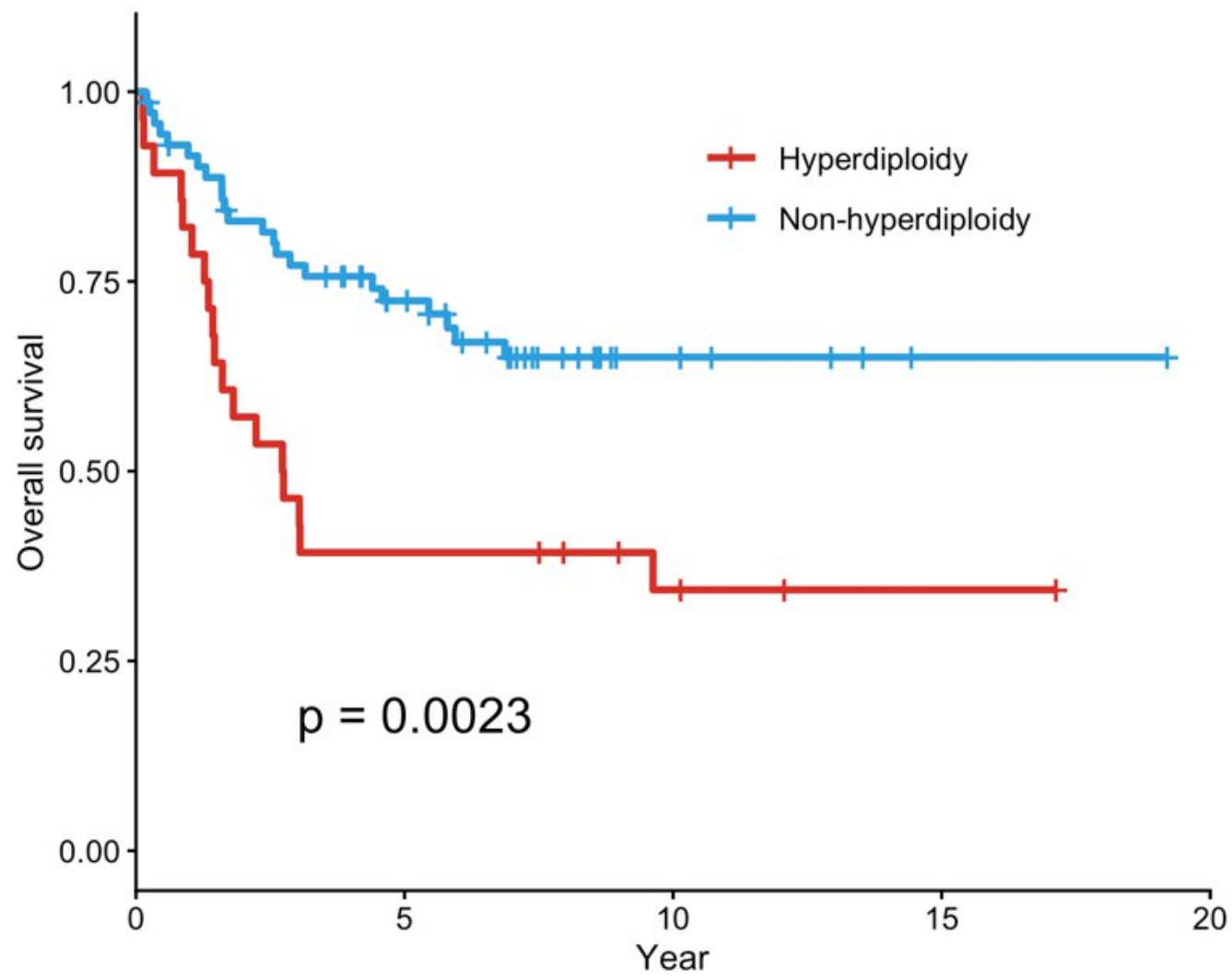

Supplementary Fig. 3. Kaplan-Meier curves for overall survival according to the ploidy

– Structural variation (SV)

Structural variations (SVs) were called when they were identified by at least 2 of 3 algorithms tested (Delly v0.7.2,[8] SvABA v1.2.0,[9] and Manta v1.6.0[10]). Throughout this study, only SVs that were identified by at least 2 algorithms were used for subsequent analyses such as chromothripsis and gene fusion analyses. The number of SVs was not associated with prognosis in our 100 patients (HR = 1.000 (95% CI, 1.000-1.001); *P* = 0.324).

RNA-seq analyses

The total RNA samples were subjected to RNA-seq analysis at DNA Link (DNA Link Inc., Seoul, Korea). The purity of RNA was determined by assaying 1 μL of total RNA extract on a NanoDrop 1000 spectrophotometer (Thermo Fisher Scientific, Waltham, MA). The integrity of RNA was checked using an Agilent 2100 Bioanalyzer (Agilent Technologies, Santa Clara, CA), expressed as an RNA Integrity Number (RIN) value. RNA-seq libraries were prepared according to the manufacturer's instructions for the TruSeq Stranded mRNA Library Prep kit (Illumina, San Diego, CA). mRNA sequencing was performed by DNA Link (DNA Link Inc., Seoul, Korea). mRNA was purified and fragmented from total RNA (1 μg) using poly-T oligo-attached magnetic beads using two rounds of purification. Cleaved RNA fragments primed with random hexamers were reverse transcribed into first strand cDNA using reverse transcriptase, random primers, dUTP in place of dTTP. These cDNA fragments then had the addition of a single 'A' base and subsequent ligation of the adapter. The products were purified and enriched with PCR to create the final strand-specific cDNA library. The quality of the amplified libraries was verified by automated electrophoresis (TapeStation, Agilent). After QPCR using KAPA SYBR FAST qPCR Master Mix (Kapa Biosystems, Wilmington, MA), we combined libraries that index tagged in equimolar amounts in the pool. Sequencing was performed using an Illumina NovaSeq 6000 system following provided protocols for 2 × 101 sequencing.

– Processing

RNA-sequenced reads were trimmed using Trimmomatic v0.39[11] to remove adapter and low-quality sequences, using default settings. Subsequently, the trimmed reads were aligned to the human genome build GRCh37 using STAR v2.7.3a.[12] To estimate gene expression levels, we used the rsem-calculate-expression script provided by RSEM v1.3.3.[13] STAR and RSEM reference libraries were created from genome build GRCh37 together with the Ensembl[14] gene model file GENCODE19.gtf. The average sequencing depths for normal and tumor samples in RNA-seq were 114.33X and 188.71X, respectively.

– Genes with *cis-* and *trans*-activation

We identified genes with *cis*-transcriptional activation by correlating SCNA with cognate TPM data. The 38 genes located at the 20 recurrent amplicons exhibited significant *cis*- and *trans*-transcriptional activation (P < 0.001 and *No.* significant genes > 500; Supplementary Table 6a). To explore which of these genes may underlie gastric carcinogenesis, we knocked out selected genes located at 6p21 (chr6:41662844 – 43681645) and evaluated the effect of gene silencing on *in vitro* proliferation of human gastric cancer cell lines (Supplementary Table 6b).
The most significant deletion was at chr3:59821440–61289532, containing *FHIT*, and the most significant amplification region was chr17:36901540–38587492, harboring *ERBB2*. The other significant amplification regions included chr8:125976787–130368582 (*MYC*), chr19:27733473–31299292 (*CCNE1*), chr7:53928203–56560299 (*EGFR*), and chr11:34929584–35578648 (*CD44*). All

significant amplification regions in this study were amplification regions that were recurrent in the prior WES-based STAD TCGA study.

– *TERT* promoter mutation with transcriptional activation

TERT promoter mutation (chr5:1295459G>A) was found in a GC with transcriptional activation of *TERT* (Supplementary Fig. 4).

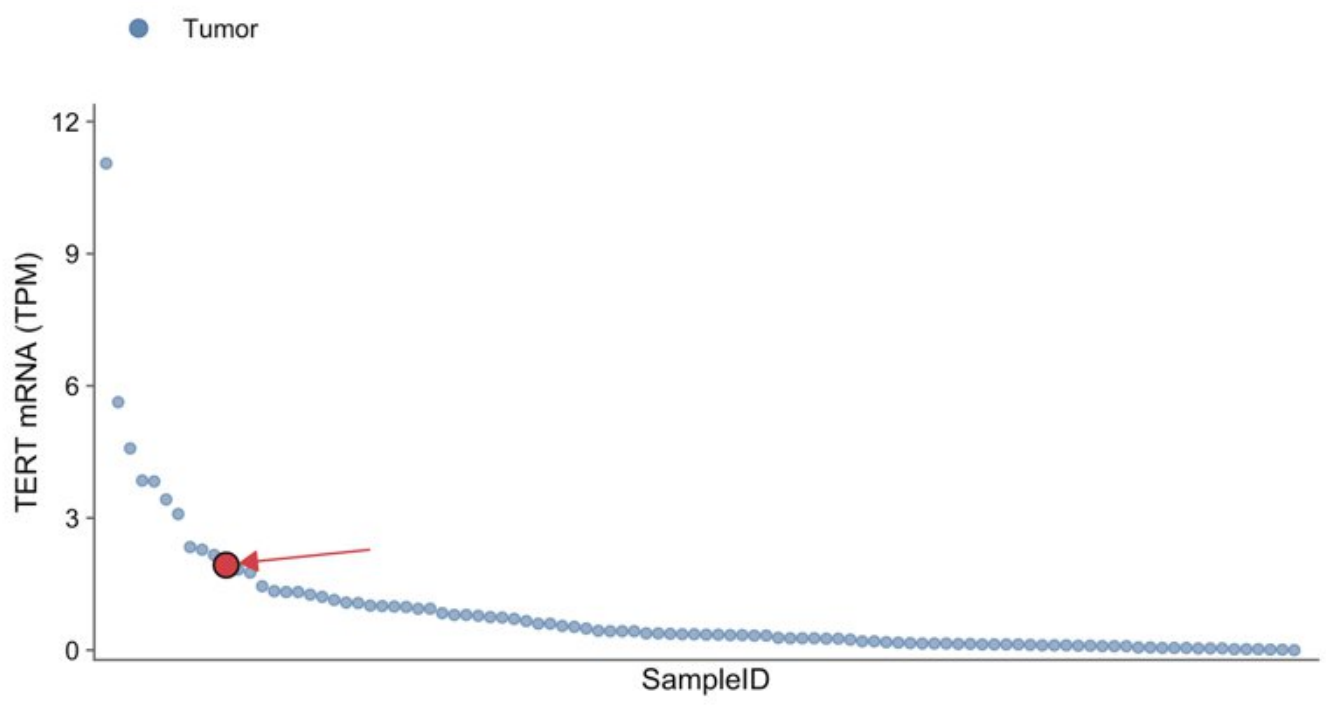

Supplementary Fig. 4. TERT mRNA expression levels (TPM). Red arrow, TERT promoter mutation (chr5:1295459G>A) with transcriptional activation

– Genomic rearrangements with transcriptional activation

This SV was associated with *IRS4* mRNA overexpression in the corresponding GC tissue. Another enhancer hijacking event involving *IGF2* gene (chr11:2138686–2265093), which was previously reported in colorectal and gastric cancers [15], was observed in a GC with mRNA overexpression of the cognate gene (Supplementary Fig. 5).

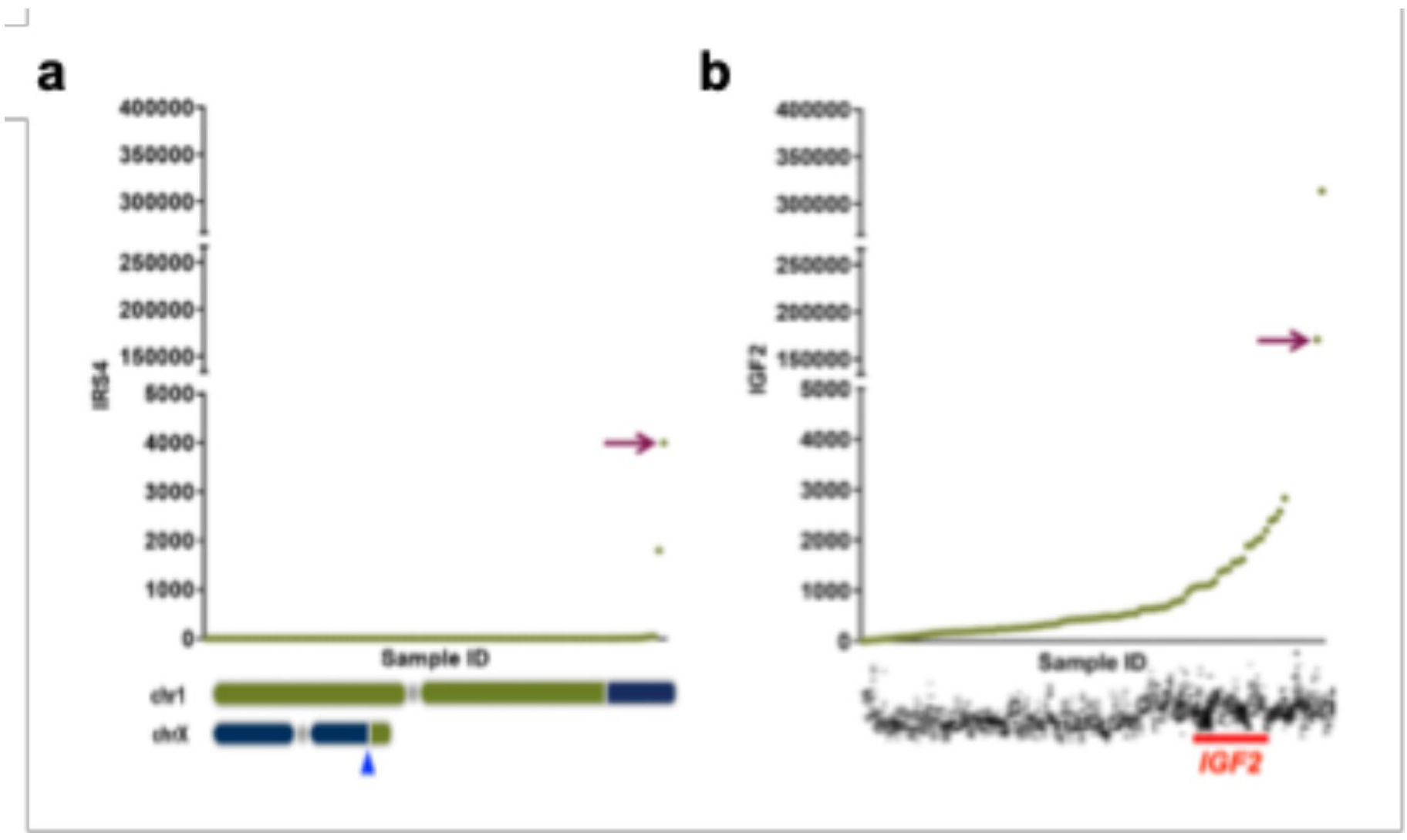

Supplementary Fig. 5. Genomic rearrangements near IRS4 and IGF2 with transcriptional activation. a SV involving a topologically associating domain (chrX: 107720001–108600000) boundary near *IRS4* gene. Bottom chart, Translocation between chrX:108150467–108150468 and chr1:234758915. Blue arrow indicates *CTCF* binding sites; Upper chart, *IRS4* mRNA expression of the GC harboring the SV (shown in red arrow) across 100 GCs. **b** Enhancer hijacking of *IGF2* with transcriptional activation. Bottom chart, copy number plot for the duplication of *IGF2* (shown as a continuous red line); Upper chart, *IGF2* mRNA expression level of the GC harboring the SV (shown in red arrow) across 100 GCs. GC, gastric cancer; SV, structural variation

– Epstein-Barr virus (EBV) status

EBV status was determined by tumor/normal TPM ratio of EBV (MK973062.1) transcript. Cutoff for tumor/normal TPM ratio defining EBV positivity was set at 100. Eleven tumors (11%) were positive for EBV. Supplementary Fig. 6 displays the distribution of the tumor/normal TPM ratio of EBV (MK973062.1) transcript across 100 GCs (Supplementary Fig. 6).

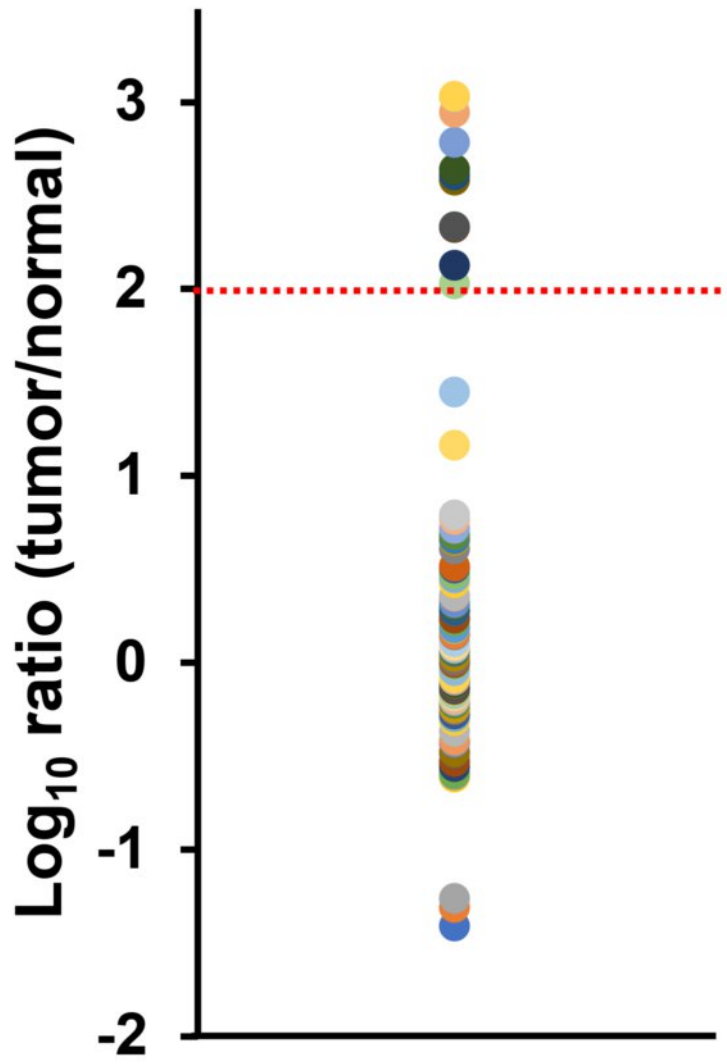

Supplementary Fig. 6. EBV transcript ratio across 100 GCs. A continuous red line indicates a cutoff (tumor/normal TPM ratio 100). EBV, Epstein-Barr virus; GC, gastric cancer.

– Gene fusion

Gene fusion calling was conducted using defuse v0.8.0.[16] All gene fusion candidates were examined for genomic evidence and gene fusions that did not overlap with SVs were excluded.

None of these in-frame gene fusions were recurrent among 100 GCs, but *ARHGEF7* was the 5' partner gene of the two in-frame fusions, *SLC1A2* and *SYT13* in a diffuse type gastric cancer. Eleven tumors expressing at least one in-frame gene fusion did not show different prognosis from those without. These results collectively suggest that gene fusion makes a limited contribution, if any, to gastric carcinogenesis in general (Supplementary Table 12).

Supplementary Table 12. In-frame gene fusions among 100 GCs.

Gene1					Gene2				
Name	Chr	Start	End	Strand	Name	Chr	Start	End	Strand
ATPIF1	1	28,562,620	28,573,417	+	*BMF*	15	40,380,091	40,401,093	-
SLC12A8	3	124,801,480	124,998,021	-	*GRHL2*	8	102,504,660	102,681,954	+
C6orf106	6	34,555,065	34,664,636	-	*OSBPL1A*	18	21,742,008	21,977,844	-
LMBR1	7	156,461,646	156,685,924	-	*EYA4*	6	133,561,736	133,853,258	+
CNTNAP2	7	145,813,453	148,118,090	+	*TMEM117*	12	44,229,770	44,783,545	+
DDX21	10	70,715,884	70,744,829	+	*CCDC146*	7	76,751,751	76,958,850	+
KIAA1217	10	23,983,675	24,836,772	+	*OR2K2*	9	114,089,763	114,090,800	-
WAC	10	28,821,422	28,912,041	+	*ZNF568*	19	37,407,231	37,489,602	+
NAA40	11	63,706,431	63,724,800	+	*LYZL2*	10	30,900,718	30,918,691	-
PDHX	11	34,937,376	35,042,138	+	*CARS2*	13	111,293,759	111,365,950	-
PAMR1	11	35,453,370	35,551,848	-	*ING1*	13	111,365,083	111,373,421	+
TMEM117	12	44,229,770	44,783,545	+	*CNTNAP2*	7	145,813,453	148,118,090	+
ARHGEF7	13	111,766,906	111,958,084	+	*SLC1A2*	11	35,272,753	35,441,610	-
CARS2	13	111,293,759	111,365,950	-	*PDHX*	11	34,937,376	35,042,138	+
CARKD	13	111,267,881	111,292,340	+	*CREB3L1*	11	46,299,212	46,342,972	+

ARHGEF7	13	111,766,906	111,958,084	+	*SYT13*	11	45,261,852	45,307,870	-
NEMF	14	50,249,997	50,319,921	-	*STAU2*	8	74,332,604	74,659,943	-
TCF12	15	57,210,821	57,591,479	+	*ELP3*	8	27,947,190	28,048,673	+
SNX1	15	64,386,322	64,438,289	+	*FGD3*	9	95,709,733	95,798,518	+
NPRL3	16	134,273	188,859	-	*PTCH2*	1	45,285,516	45,308,735	-
PHF12	17	27,232,268	27,278,789	-	*SP100*	2	231,280,657	231,408,805	+
GSDMB	17	38,060,848	38,076,107	-	*PLRG1*	4	155,456,158	155,471,587	-
RAB12	18	8,609,443	8,639,379	+	*SPICE1*	3	113,161,565	113,234,034	-
DNM2	19	10,828,755	10,944,164	+	*C1orf222*	1	1,853,396	1,935,276	-
TTC3	21	38,445,526	38,575,413	+	*STK3*	8	99,413,631	99,955,055	-

In-frame gene fusion-positive tumors (*n* = 11) were not significantly associated with overall survival compared with non-fusion tumors (*P* for log-rank = 0.74; HR = 1.17 (95% CI, 0.46-2.99); *P* = 0.74). Median survival times were not reached (95% CI, 0.14-not reached) and not reached (95% CI, 5.8-not reached), in patients with fusion-positive and non-fusion tumors, respectively (Supplementary Fig. 7).

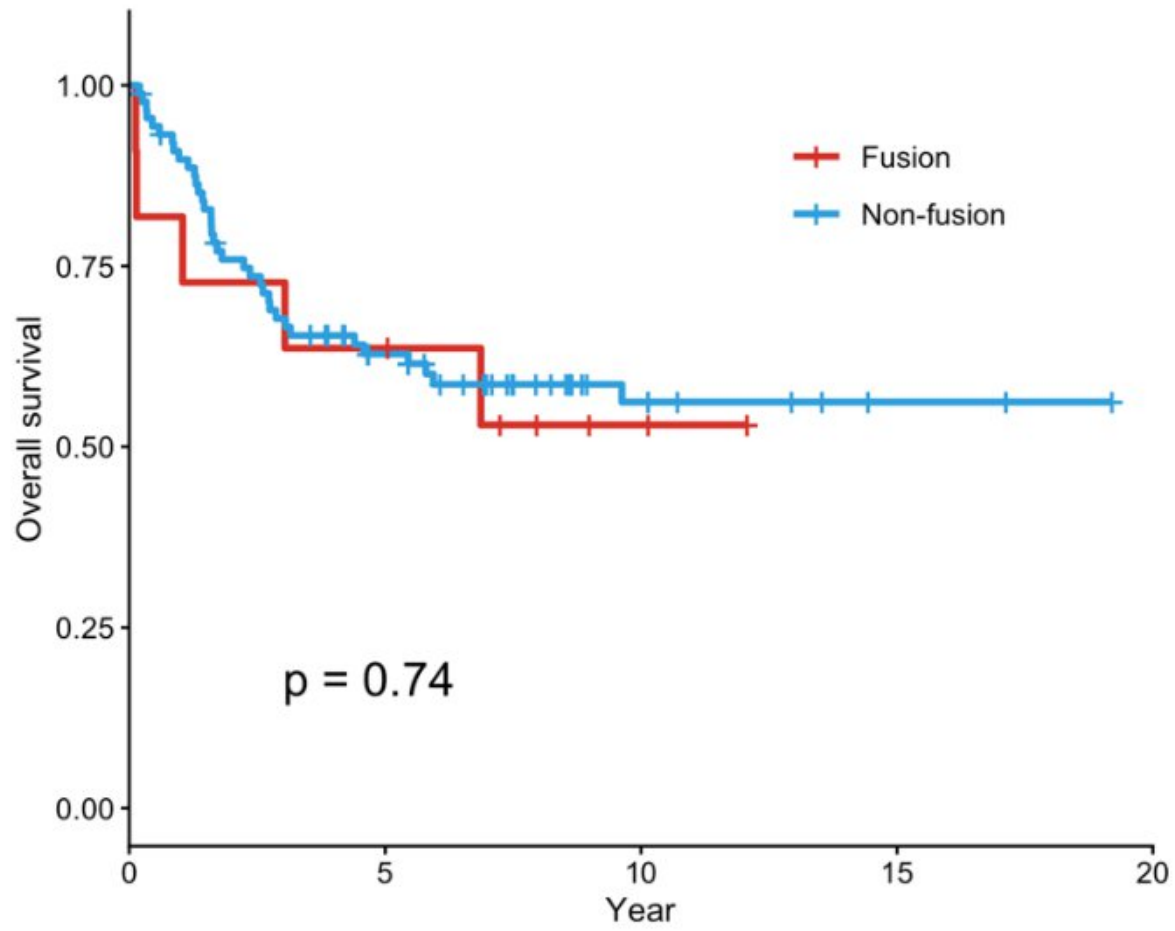

Supplementary Fig. 7. Kaplan-Meier curves for overall survival according to In-frame gene fusion status.

– 3’ gene overexpression and fusion breakpoints

For fusion-associated expression analysis, the expression level of each 3′ partner gene was examined across 100 samples. A Z-score was calculated relative to the cohort-wide distribution, and genes with Z-scores ≥ 1.96 were defined as significantly overexpressed (Fig. 4h).

Methylation sequencing analyses

– Processing

Matched tumor-normal gastric adenocarcinoma samples were profiled with targeted bisulfite sequencing and whole-genome sequencing (WGS). Tumor and paired normal specimens were processed in parallel for paired methylation analysis. Bisulfite libraries were prepared using the Agilent SureSelect XT Methyl-Seq workflow and sequenced on Illumina platforms. Adapter-trimmed reads were aligned to hg19/GRCh37 using Bismark (v0.22)[17] with paired-end defaults. Methylation calls were generated with `bismark_methylation_extractor` (`-p --no_overlap --comprehensive --report --gzip --ignore 3 --multicore 4 --bedGraph --cytosine_report`). Cytosine-level output included chromosome, 1-based coordinate, methylated read count, and total read count. CpG positions were matched to Illumina Infinium MethylationEPIC v2.0 probe coordinates after GRCh38-to-hg19 coordinate conversion. Matched probes were intersected with SureSelect XT Methyl-Seq target intervals (84 Mb), and artefactual interval expansions in pericentromeric regions were removed. Beta values were calculated as:

$$\beta = \frac{\sum methylated\ reads}{\sum total\ reads}$$

At each CpG, samples with total depth < 5x were excluded. CpGs were retained when >=50% of samples met coverage criteria. After on-target and coverage filtering, 440,234 CpGs were retained. Probe annotation used EPIC v2 manifest categories, including CpG island relation and gene context. To account for tumor purity, PureBeta[18] was used to adjust tumor β values according to tumor purity. For each tumor-normal pair, cancer-specific methylation change was calculated as paired Δβ:

$$\Delta\beta = \beta_{tumor} - \beta_{normal}$$

– Global methylation in the tumor

Global methylation features were defined across the same 440,234 on-target CpGs. Probes were classified as hypermethylated when $\Delta\beta > +0.2$ and hypomethylated when $\Delta\beta < -0.2$, and the per-sample fractions of hypermethylated and hypomethylated probes were computed (HR = 1.03 (95% CI, 0.99-1.06); $P = 0.12$).

– CpG island methylator phenotype (CIMP)

CpG island methylator phenotype (CIMP) was assessed with the Weisenberger five-gene panel (*CACNA1G, IGF2, NEUROG1, RUNX3, SOCS1*)[19]. Among on-target probes, CpG-island probes annotated to promoter-proximal regions (TSS200 or TSS1500) were selected for these genes (68 probes total). Per gene, mean Δβ was computed across constituent probes; a locus was considered methylated when mean

Δβ > +0.10. Tumors were categorized into CIMP-H (≥ 3/5 loci methylated) and non-CIMP (0–2/5).

COSMIC mutational signature

– SBS, DBS, ID, and SV mutational signatures

To characterize mutational processes, we analyzed single base substitutions (SBS), doublet base substitutions (DBS), small insertions and deletions (ID), and structural variations (SV).
SBS signatures were extracted using SignatureAnalyzer[20], a non-negative matrix factorization-based approach. For DBS, ID, and SV signatures, we applied SigProfiler (https://github.com/AlexandrovLab) and annotated signatures based on the COSMIC reference database.
For visualization, samples with high signature exposure (top 25%) were selected for each signature category (SBS, DBS, ID, and SV).
Survival analyses were performed for all identified SBS, DBS, ID, and SV signatures using univariate Cox proportional hazards regression, and the results are summarized in Supplementary Table 4a.

– Mutational signature refitting and survival analysis

Mutational signature refitting was performed using SigProfilerAssignment[21] separately for single-base substitution (SBS), doublet-base substitution (DBS), and small insertion and deletion (ID) signatures. Signature contributions were calculated as the percentage of each signature activity relative to the total refitted activity within each sample and mutation class. Associations between signature contributions and overall survival were assessed using univariate Cox proportional hazards regression, with hazard ratios representing the change in hazard per 1 percentage-point increase in signature contribution (Supplementary Table 4b).

– MSI mutational signature

Mutational signature analysis demonstrated enrichment of MSI-associated signatures in MSI gastric cancers (GCs) compared with non-MSI GCs. Specifically, increased contributions of DBS7 and SBS44, along with elevated proportions of SBS15 and SBS6, were observed in MSI GCs (n = 19), whereas these signatures were largely absent or showed low activity in non-MSI GCs (n = 81). Consistent with these findings, MLH1 promoter methylation and MLH1 truncating alterations were predominantly detected in MSI GCs, supporting mismatch repair deficiency as a major contributor to the observed mutational landscape (P < 0.001; Supplementary Fig. 8).

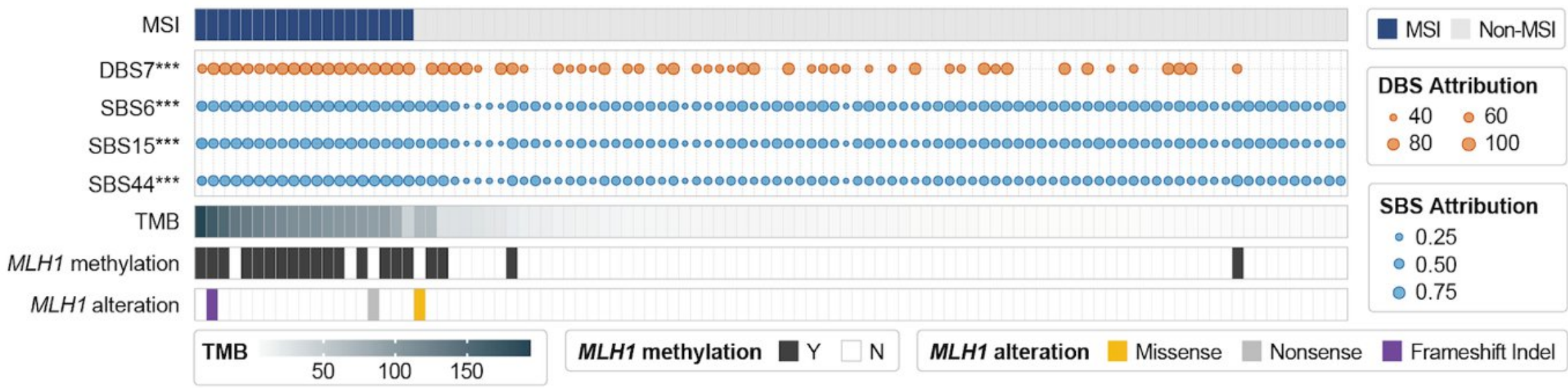

Supplementary Fig. 8. MSI signature. DBS7, SBS6, SBS15, SBS44 signatures were more strongly enriched in MSI GCs (n = 19; left) than in non-MSI GCs (n = 81; right) at $P < 0.001$. ***, $P < 0.001$.

For *MLH1* methylation analyses, promoter methylation levels were quantified using β-values derived from DNA methylation data, where β-values range from 0 to 1. For *MLH1* gene, promoter regions were defined based on annotated transcription start sites, and the average β-value across probes mapped to the promoter region was calculated for both tumor and matched normal samples.

To assess differential methylation, the change in promoter methylation between tumor and normal tissues was calculated as:

$$\Delta\beta = \beta_{tumor} - \beta_{normal}$$

Gene expression levels were quantified using transcripts per million (TPM) from RNA-seq data. Promoter methylation–associated gene silencing was defined as cases exhibiting both increased promoter methylation in tumor tissue ($\Delta\beta > 0.15$) and reduced gene expression in tumor samples ($TPM_{tumor} < 3$).

– CN mutational signature

Copy number (CN) signature analysis was conducted using SigProfiler. *k*-means clustering of CN signature profiles identified two subgroups (k = 2).
To gain insight into the etiologies of CN19 and CN20, which remain undefined, we assessed their associations with other genomic instability measures. Among these, CN20 attribution was significantly correlated with the number of chromothripsis events in 100 gastric cancers (R = 0.248, P = 0.013; Supplementary Table 13). These results might suggest possible etiologic links of CN19 and CN20 signatures to HRD or chromothripsis.

Supplementary Table 13. Mean attribution of CN signatures according to the chromothripsis. CN, copy number.

	CN4	CN5	CN6	CN7	CN8	CN20	CN9
Chromothripsis^{+}	0.120	0.030	0.122	0	0.024	0.155	0.320
Chromothripsis^{-}	0	0	0.013	0.071	0.032	0.023	0.693
P for *t*-test	0.001	0.017	0.010	0.163	0.817	0.001	< 0.001

Data in the literature suggests that CN4, CN5, CN6, CN7 and CN8 are associated with chromothripsis, while the etiologies of CN19, CN20, and CN21 are unknown.
The attribution of CN19 signature significantly correlated with SBS3 signature (*R* = 0.3686; *P* = 0.0002; Supplementary Fig. 9).

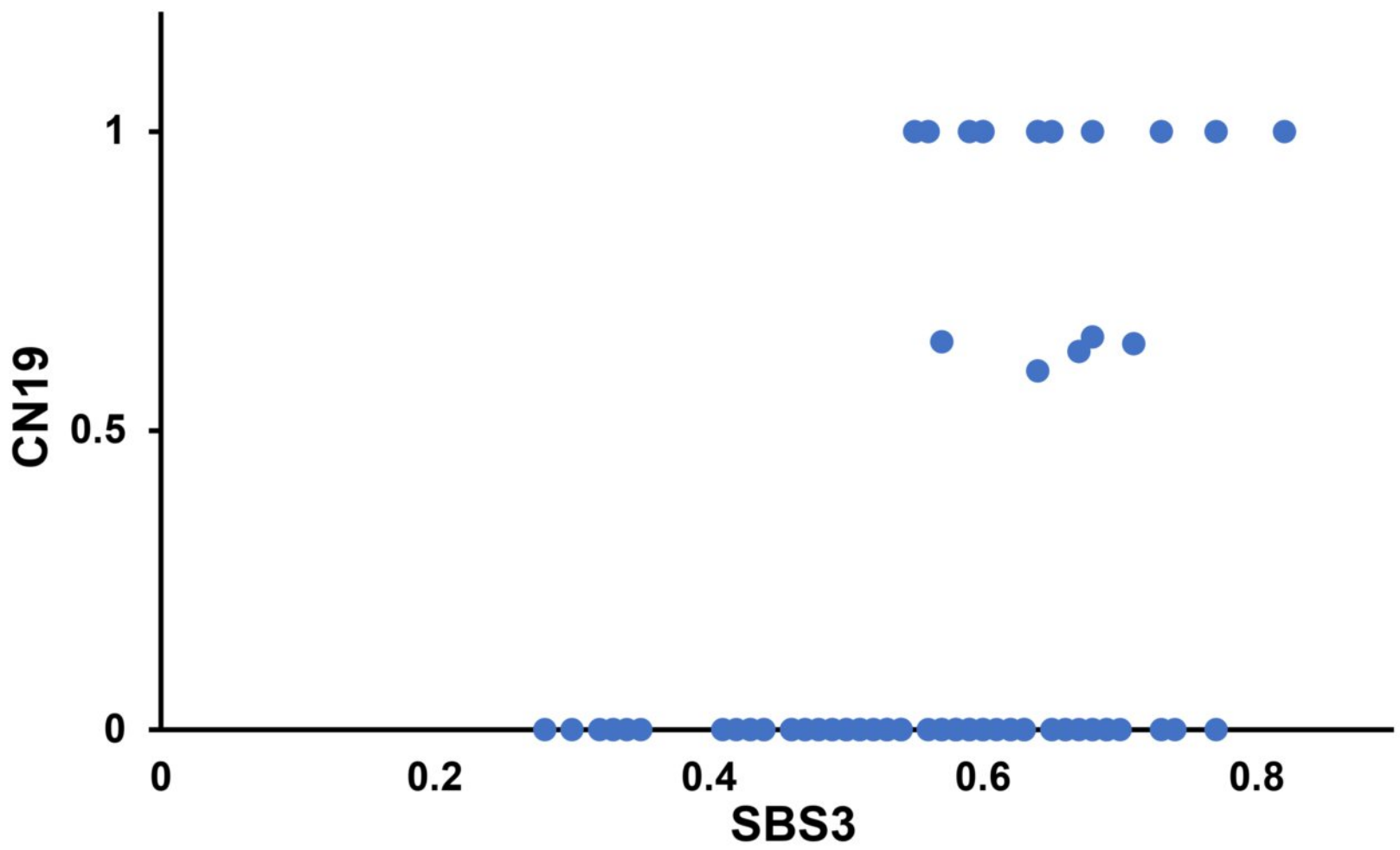

Supplementary Fig. 9. Correlation between CN19 and SBS3 mutational signatures. CN, copy number; SBS, single base substitution.

The distribution of TCGA molecular subtype was different between CN9 and non-CN9 GCs (*P* for Fisher's exact test < 0.0001) (Supplementary Table 14).

Supplementary Table 14. Molecular subtype of GC according to CN signature. GC, gastric cancer; CN, copy number

TCGA molecular subtype	CN9	non-CN9
EBV	10 (16.3%)	1 (2.6%)
MSI	18 (29.5%)	1 (2.6%)
GS	18 (29.5%)	11 (28.2%)
CIN	15 (24.6%)	26 (66.7%)
Total	61	39
P for Fisher's exact test	< 0.0001	

– **Microenvironment analysis by CN9 status**

To investigate the tumor microenvironment associated with copy number (CN) signature subtypes, we compared stromal and immune components between CN9$^+$ and non-CN9 (CN9$^-$) groups.

ESTIMATE[22] analysis revealed that CN9$^+$ tumors exhibited higher stromal and immune scores compared to non-CN9 tumors, indicating increased non-tumor cell infiltration. Overall ESTIMATE scores were lower in the non-CN9 group (Supplementary Fig. 10).

In summary, CN9$^+$ tumors had significantly higher ESTIMATE (P = 0.0145) and immune scores (P = 0.0112) than non-CN9 tumors, while the difference in stromal scores did not reach statistical significance (P = 0.0594).

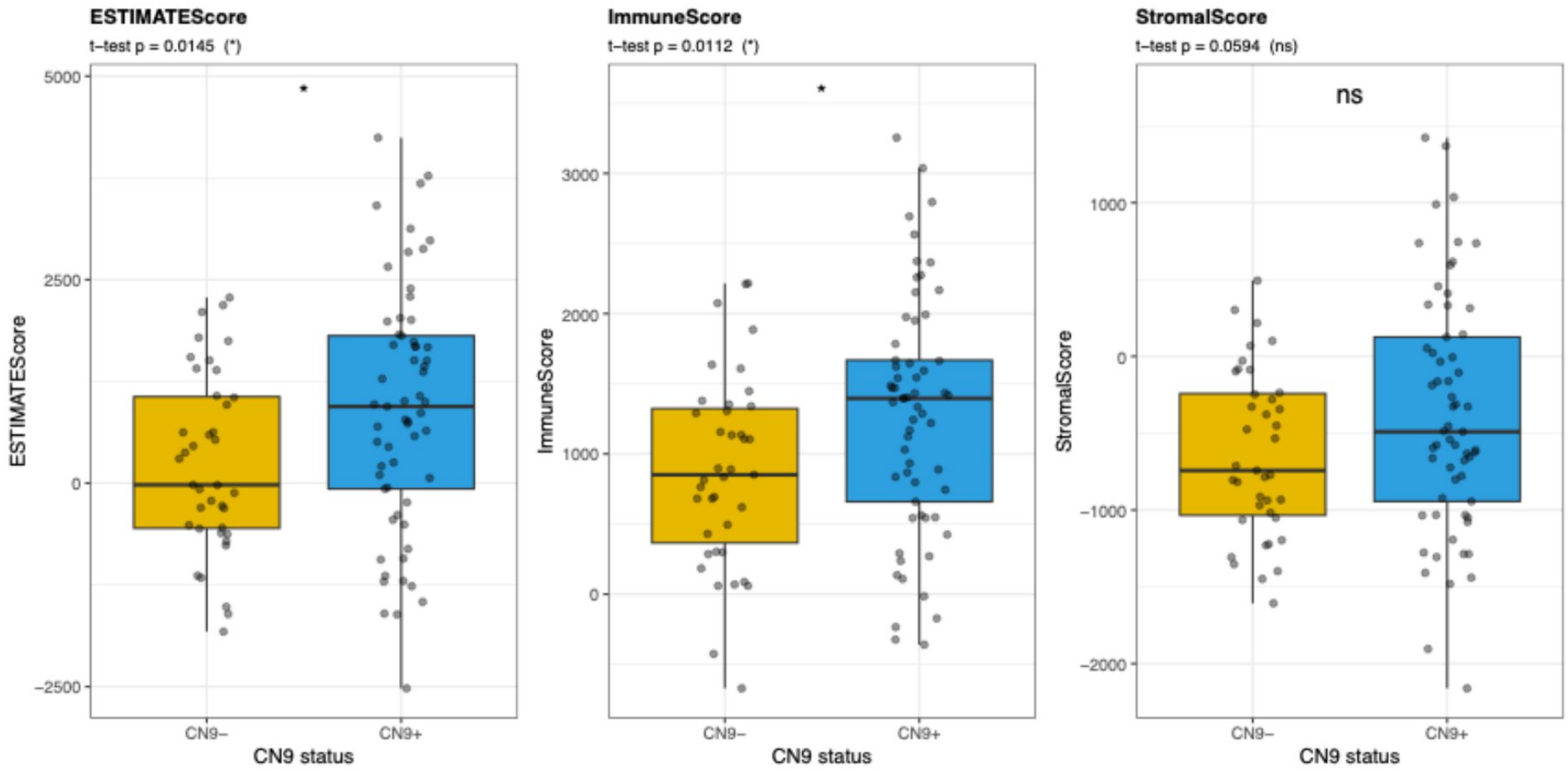

Supplementary Fig. 10. Stromal and immune infiltration across CN9 subgroups. ESTIMATE analysis revealed increased immune and overall microenvironment scores in CN9$^+$ tumors compared to non-CN9 tumors. Statistical significance was assessed using a two-sided t-test.

To further characterize immune cell composition, we performed CIBERSORT[23] analysis across CN9 subtypes. The relative proportions of individual immune cell types showed heterogeneous patterns, with no consistent enrichment of cytotoxic immune populations (Supplementary Fig. 11).

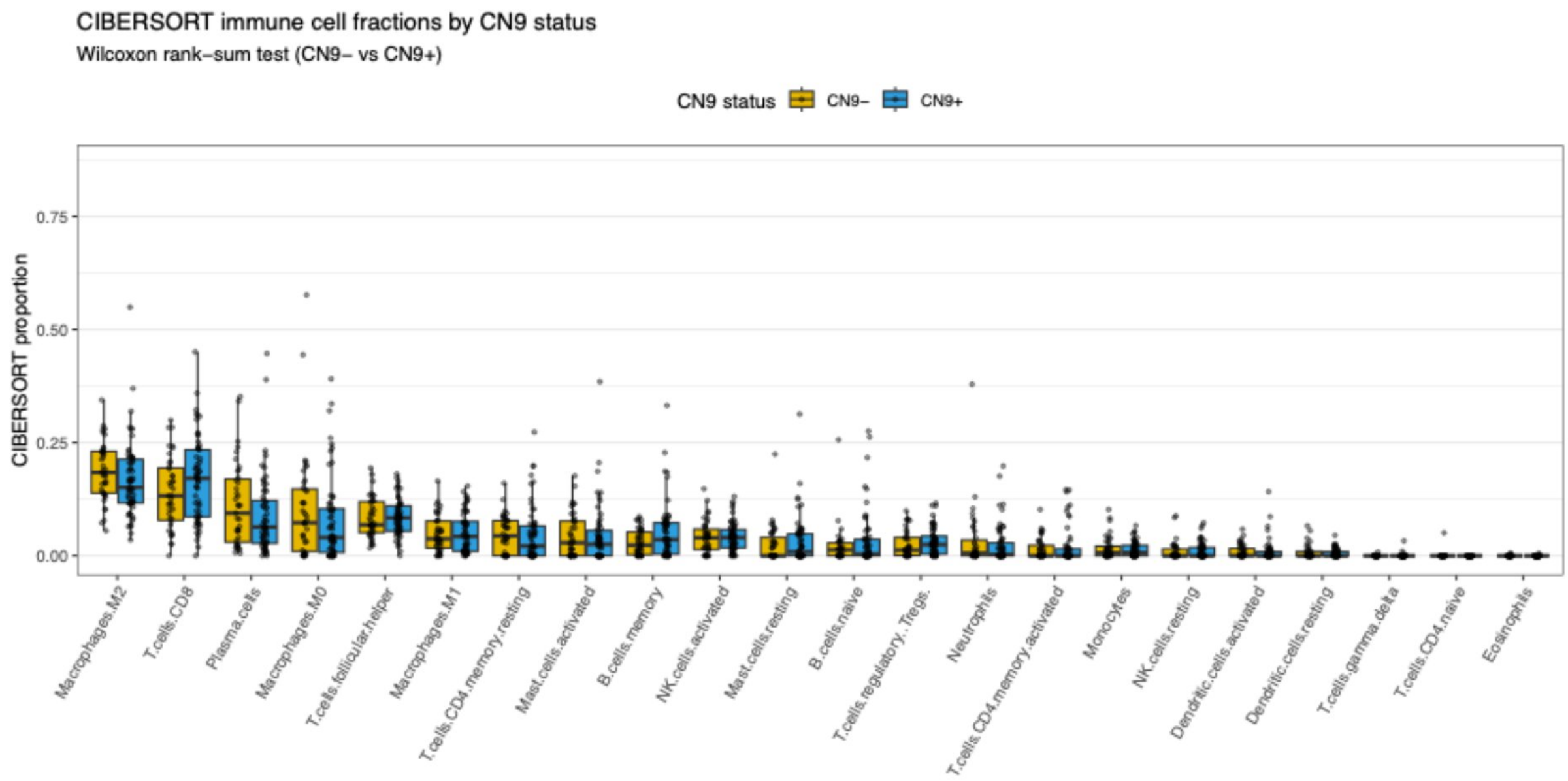

Supplementary Fig. 11. CIBERSORT immune fractions by CN9 status.

Tumor mutation burden (TMB)

Tumor mutation burden (TMB) was estimated by dividing all genome-wide somatic SNVs and indels by 3,096 Mb (Supplementary Fig. 12). Hypermutated samples (*n* = 21; TMB > 40/Mb) were removed from MutSig2CV v3.11[4] analysis. As a result of MutSig2CV analysis, we identified 13 somatic mutations with the evidence for protein expression according to global proteomic profiling analysis of the same samples (Supplementary Table 3).

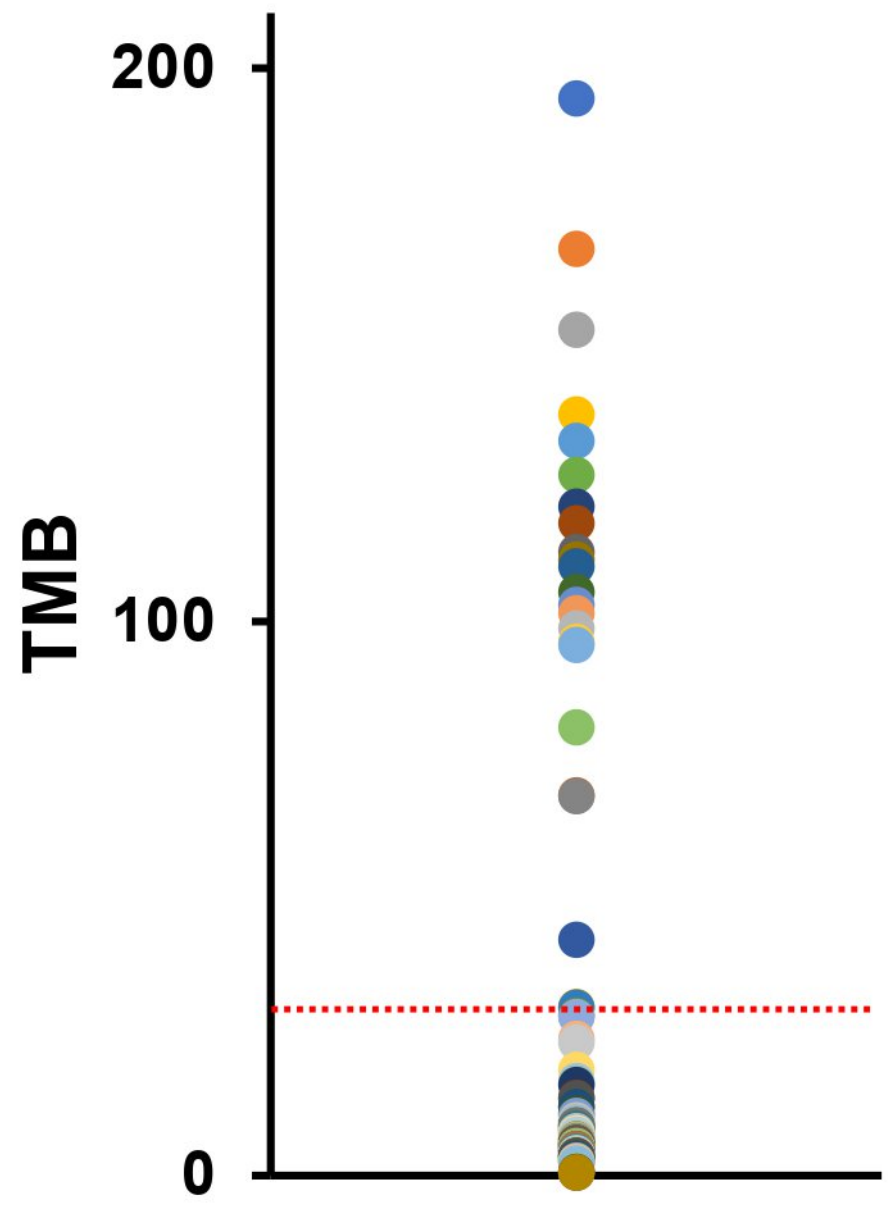

Supplementary Fig. 12. TMB in log scale across 100 GCs. A dotted red line representing median TMB (7.6/Mb). TMB, tumor mutation burden.

In this study, a stringent TMB criterion was set for defining hypermutated tumors. Hypermutated tumors (TMB > 40/Mb; *n* = 21) showed stronger MMR-related COSMIC mutational signatures (SBS6 and SBS15; Supplementary Fig. 13), and more favorable prognosis than non-hypermutated tumors (*P* for log-rank = 0.016; HR = 0.26 (95% CI, 0.08–0.85); *P* = 0.034; Supplementary Fig. 14).

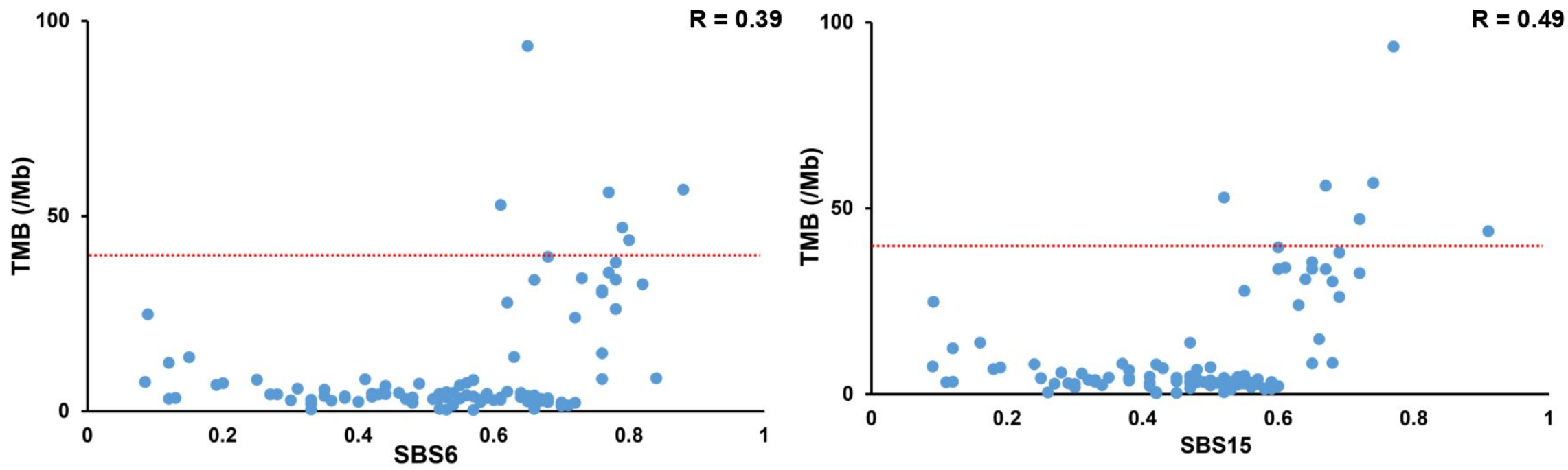

Supplementary Fig. 13. Mismatch repair defect-related COSMIC mutational signature activities according to TMB. SBS6 and SBS15 signatures were more active in hypermutated tumors (TMB > 40/Mb; *n* = 21) than in non-hypermutated tumors. A dotted red line representing the cutoff for defining hypermutation (40/Mb). TMB, tumor mutation burden.

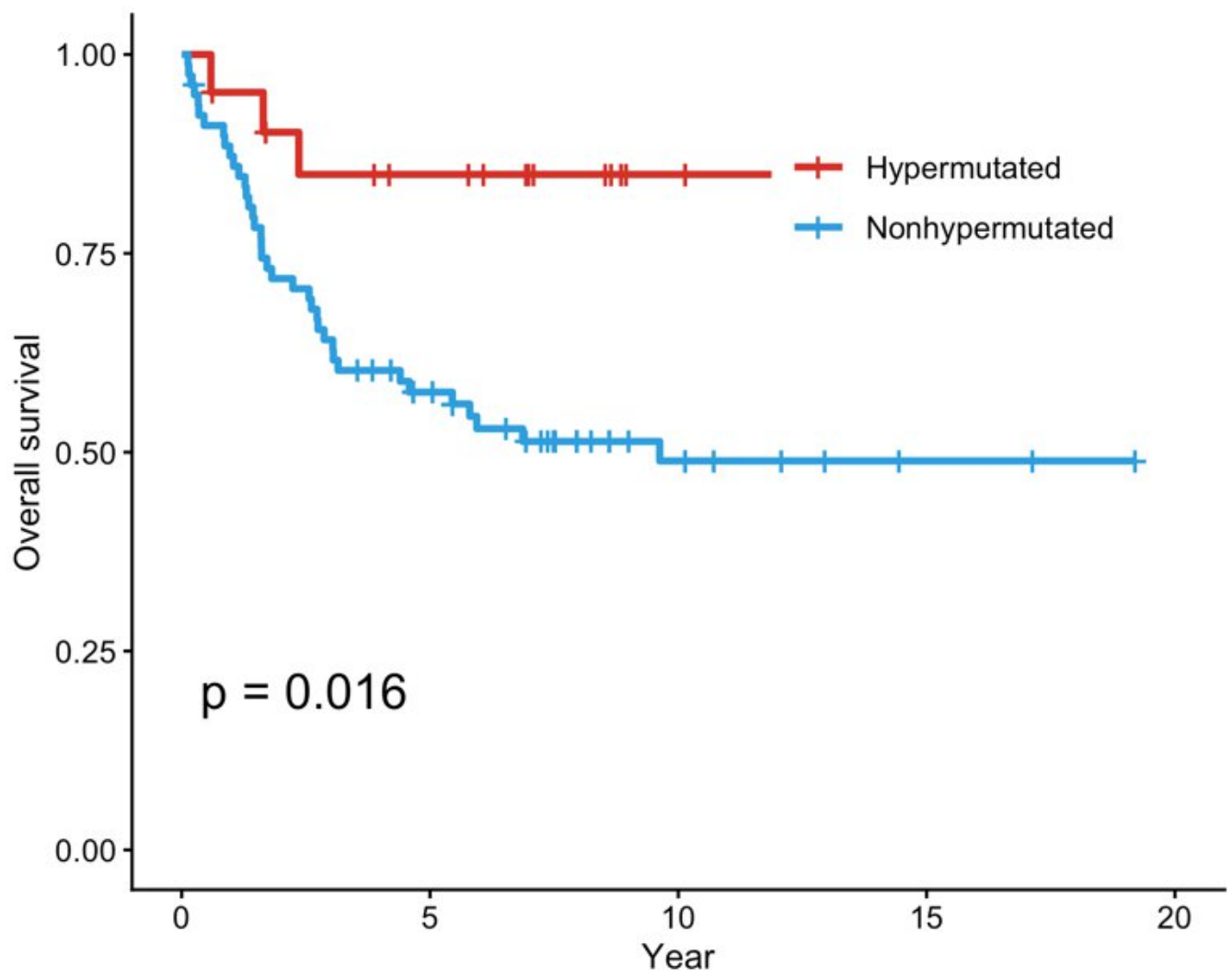

Supplementary Fig. 14. Kaplan-Meier curves for overall survival according to the TMB. Red and blue lines representing hypermutated and non-hypermutated tumors, respectively. TMB, tumor mutation burden.

Microsatellite instability (MSI)

Microsatellite instability (MSI) was evaluated by an in-house analysis method for next generation sequencing data (Patent KR10-2529641). Briefly, we created a matrix for the following mononucleotide repeat loci[24] using the pileup2acgt function from the Sequenza v3.0.0 R package[6] and mpileup output of SAMtools v1.7[2] (Supplementary Table 15).

Supplementary Table 15. Thirteen genomic loci used for calculating MSI score. MSI, microsatellite instability

No.	Genomic loci	Mapped base count
1	CHR1	201,754,411–201,754,414 (TTTT)
2	CHR2	108,479,623–108,479,628 (TTTTTT)
3	CHR4	55,598,212–55,598,218 (TTTTTTT)
4	CHR5	172,421,761–172,421,765 (TTTTT)
5	CHR6	142,691,951–142,691,955 (TTTTT)
6	CHR7	1,787,520–1,787,524 (AAAA)
7	CHR7	74,608,741–74,608,743 (TTT)
8	CHR11	106,695,515–106,695,517 (TTT)
9	CHR13	31,722,621–31,722,626 (AAAAAA)
10	CHR14	23,652,347–23,652,352 (AAAAAA)
11	CHR15	45,897,772–45,897,774 (TTT)
12	CHR16	18,882,660–18,882,663 (AAA)
13	CHR17	19,314,918–19,314,922 (TTTTT)

We then compared the distribution of base count mapped to these loci between tumor and normal samples of the same patients and determined the number of genomic loci (out of 13 loci) that demonstrated significantly ($P < 0.05$) more deletion in the tumor compared with normal genome (MSI score). Tumor was classified as high-level MSI (MSI-H), if MSI score was ≥ 7 (Supplementary Fig. 15). This method demonstrated 100% specificity and 90.5% sensitivity for detecting tumors with TMB > 40/Mb among our 100 GCs (Supplementary Table 16).

Supplementary Table 16. TMB according to MSI score. MSI, microsatellite instability; MSS, microsatellite stable; TMB, tumor mutation burden.

No.	MSI score	Call	TMB (/Mb)
1	9	MSI	194.5
2	11	MSI	167.4
3	11	MSI	152.8
4	9	MSI	137.5
5	7	MSI	132.7
6	8	MSI	126.6
7	10	MSI	120.8
8	9	MSI	117.8
9	8	MSI	112.6
10	9	MSI	111.2
11	7	MSI	110.1

12	11	MSI	105.5
13	9	MSI	103.1
14	9	MSI	101.6
15	10	MSI	98.8
16	8	MSI	96.4
17	9	MSI	95.9
18	12	MSI	81.0
19	2	MSS	68.7
20	4	MSS	68.5
21	7	MSI	42.5

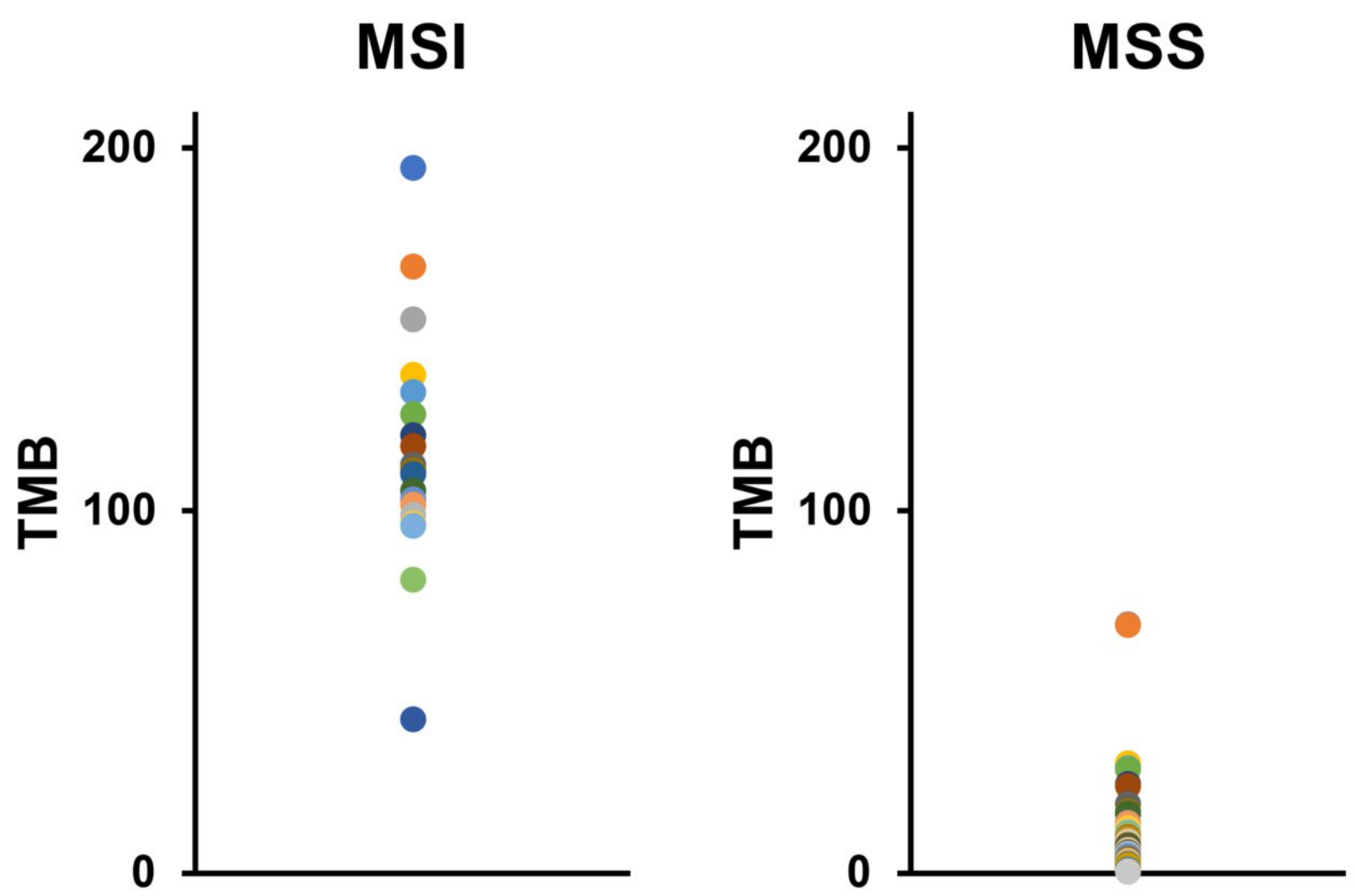

Supplementary Fig. 15. TMB (per Mb) between MSI (MSI score ≥ 7) and MSS (MSI score < 7) groups. MSI, microsatellite instability; MSS, microsatellite stable; TMB, tumor mutation burden.

TCGA subgroup

GISTIC2 v2.0.23[7] data were subjected to hierarchical clustering along with TCGA tumors whose CIN statuses were previously defined[25]. *K*-means cluster analysis (k = 2) was performed for thresholded copy number at alteration peaks from GISTIC2 analysis. The CIN status of 100 GCs in our cohort was determined based on the cluster membership.

All tumors were categorized into one of four subtypes EBV-positive (EBV), MSI-high (MSI-H), genomically stable (GS) and chromosomal instability (CIN)[25]. CIN-positive tumors without EBV or MSI were assigned to the CIN subgroup, and CIN-negative tumors without EBV or MSI were assigned to the GS subgroup. If a tumor was both EBV and CIN, that sample was assigned to the EBV group. If a tumor was both MSI-H and CIN, that sample was assigned to the MSI-H group.

H. pylori status

Presence of *H. pylori* (*Helicobacter pylori*) was identified either by CLO-positivity or by light microscopic evaluation of pathology slide.

When hypomethylation was compared in a subset of known *H. pylori* status, *H. pylori*-positive patients (n = 23) and *H. pylori*-negative patients (n = 8) were not different in the number of hypomethylated CpGs in the tumor (P for t-test = 0.90; Supplementary Table 11b).

Chromothripsis

For detection of chromothripsis, we used ShatterSeek v1.1 R package[26]. Briefly, high-confidence calls required at least 6 SVs within the cluster, 7 contiguous segments oscillating between 2 copy number states, accompanied by significant (P < 0.001) fragment joins test, and either the chromosomal enrichment or the exponential distribution of breakpoints test. High-confidence calls were visually inspected, as with the PCAWG Consortium study[26]. Throughout this study, we employed previously validated algorithms to evaluate chromosomal instability in the whole genome sequencing data. Efforts were taken to minimize false positive and negative calling of SV and chromothripsis. For example, SVs were called only when they were identified by at least 2 algorithms, and chromothripsis callings were visually confirmed.

Multichromosomal chromothripsis event was called when P value for the fragment joins test considering the inter- and intra-chromosomal SVs mapped to the cluster region was < 0.001, with the same requirements for the number of SV, contiguous segments oscillating between 2 copy number states, and chromosomal enrichment/exponential distribution of breakpoints test (Supplementary Fig. 16).

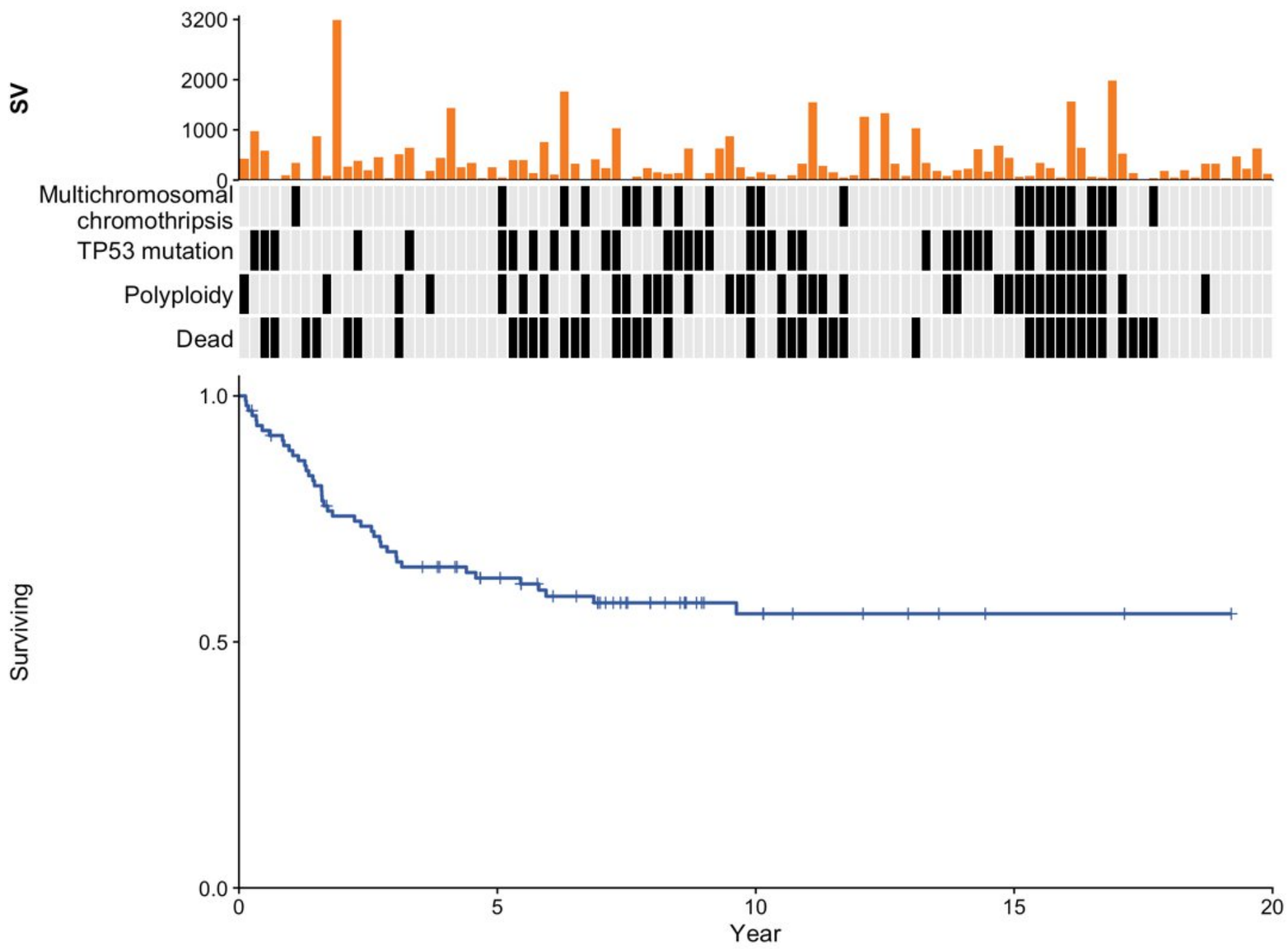

Supplementary Fig. 16. Structural variation burden and overall survival. Top histogram, number of SVs in each tumor. Each column represents a gastric cancer sample. Middle panels, black bars indicate the presence of the corresponding clinicopathologic feature or genomic alteration. Cases are ordered from left to right according to increasing follow-up duration and matched to the Kaplan–Meier curve for overall survival after surgery shown at the bottom.

Significant amplification regions were preferentially localized to chromothripsis regions (P for Fisher's exact test = 0.029). Focal amplification in the chromothripsis region included known oncogenes such as *MYC* (2 tumors) and *CCNE1* (1 tumor), with respective mRNA overexpression (Supplementary Fig. 17). *CDKN2A* deletions in two

tumors were localized to the chromothripsis regions, as reported in chordoma cell lines,[27] but chromothripsis events were not enriched in significant deletion regions (Supplementary Table 17).

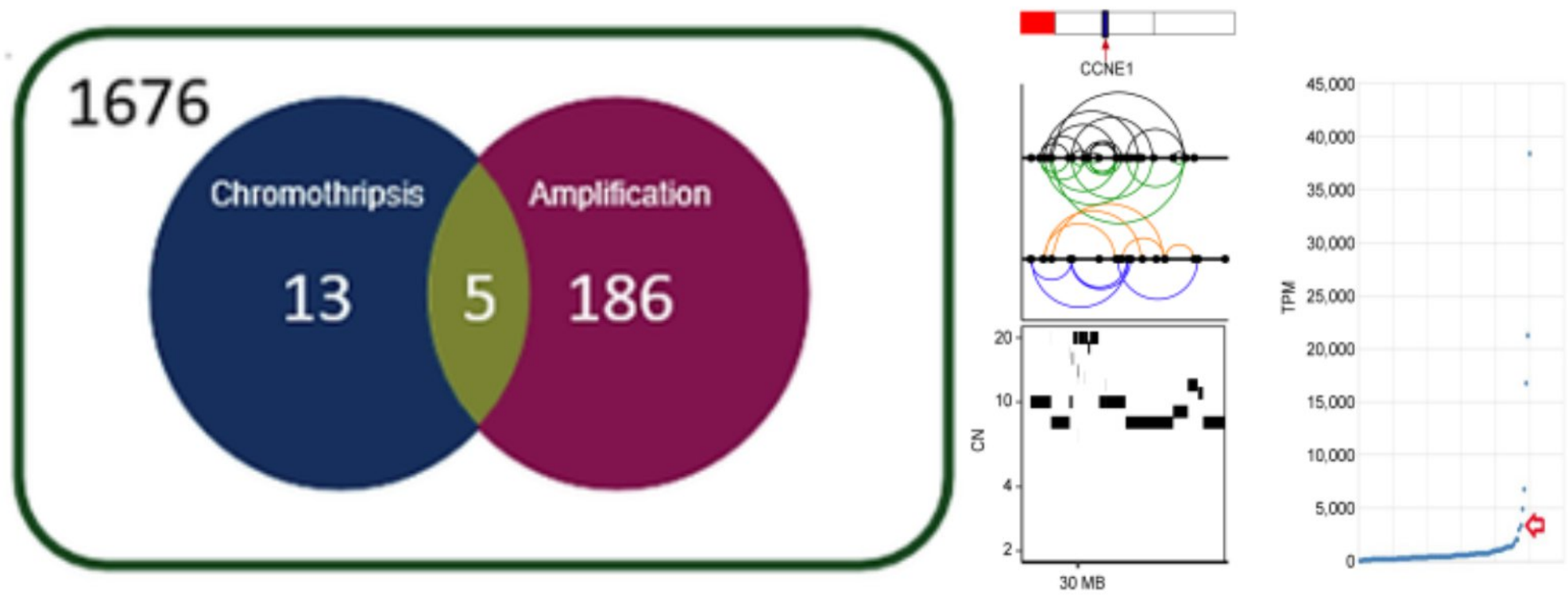

Supplementary Fig. 17. Significant amplification regions were preferentially localized to chromothripsis regions. **a** The number of significant amplification regions that overlap with chromothripsis is shown in the region where two circles overlap (*P* for Fisher's exact test = 0.029). **b** Chromothripsis-mediated amplification of *CCNE1*. Left, Shatterseek-generated display for a representative chromothripsis event with *CCNE1* amplification. Upper chart, Ideogram (19q12). Red rectangle, centromere; Middle chart, SVs depicted as arcs with the breakpoints represented by black points. The breakpoints corresponding to interchromosomal SVs depicted as colored points. Blue, orange, black, and green represent duplication-like SVs, deletion-like SVs, head-to-head and tail-to-tail inversions, respectively.; Bottom chart, Total CN profile. Black rectangle, CN segment. Right, *CCNE1* mRNA expression level of the GC harboring the chromothripsis (shown in red arrow) across 100 GCs.

Overlap between significant deletion regions and chromothripsis was not significant (*P* for Fisher's exact test = 0.79; Supplementary Table 17).

Supplementary Table 17. Overlap between significant ($Q < 0.05$) deletion region and chromothripsis region.

	Deleted	Not deleted
Chromothripsis	5	9
No chromothripsis	811	1,149

Twenty-one of 38 (55.3%) chromothripsis events in 17 patients involved multiple chromosomes. Patients with multichromosomal chromothripsis event (*n* = 17) had poorer overall survival than those without (*P* for log-rank = 0.002; HR = 2.75 (95% CI, 1.40 – 5.42); *P* = 0.003). Median survival times were 3.04 years (95% CI, 1.04–not reached) and not reached, in patients with and without multichromosomal chromothripsis, respectively (Supplementary Fig. 18).

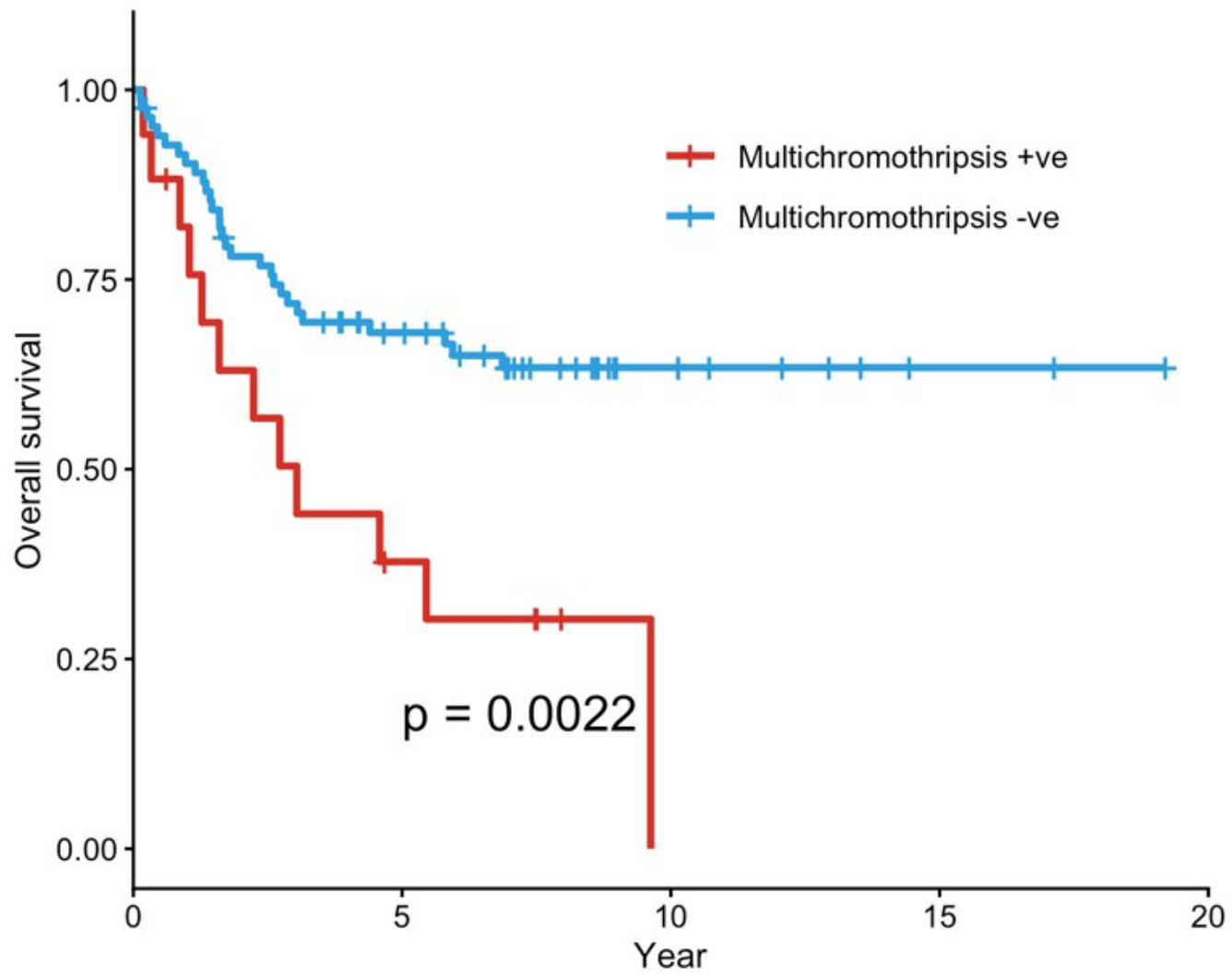

Supplementary Fig. 18. Kaplan-Meier curves for overall survival according to the multichromosomal chromothripsis event.

Throughout this study, we employed previously validated algorithms to evaluate chromosomal instability in the whole-genome sequencing data. Efforts were taken to minimize false-positive and false-negative calls of SV and chromothripsis. For example, SVs were called only when they were identified by at least 2 algorithms, and chromothripsis callings were visually confirmed. The resulting data is the first to highlight that chromothripsis is poorly prognostic.

Extrachromosomal circular DNA (ecDNA)

Extrachromosomal DNA (ecDNA) was identified using the AmpliconSuite pipeline (https://github.com/AmpliconSuite). AmpliconSuite-pipeline v1.4.0 was run with paired tumor–normal BAM files using the GRCh37 reference genome. Copy number profiles were generated using CNVkit[28], followed by amplicon reconstruction with AmpliconArchitect[29] and classification of amplicon structures using AmpliconClassifier[30]. Default parameters were applied unless otherwise specified. ecDNA-positive samples were defined based on AmpliconClassifier annotations indicating circular amplicon structures. Based on this analysis, focal amplifications were classified into circular, breakage–fusion–bridge (BFB), heavily rearranged, linear, and no focal somatic copy-number alteration categories. Among the analyzed samples, circular amplicons were identified in 31 cases (31%), whereas 7 cases (7.0%) were classified as BFB, 5 cases (5%) as heavily rearranged, and 5 cases (5%) as linear. The remaining 52 cases (52%) showed no focal somatic copy-number alterations. In this study, only circular ecDNAs were considered, and 31 GCs (31%) were identified as ecDNA-positive (Supplementary Fig. 19).

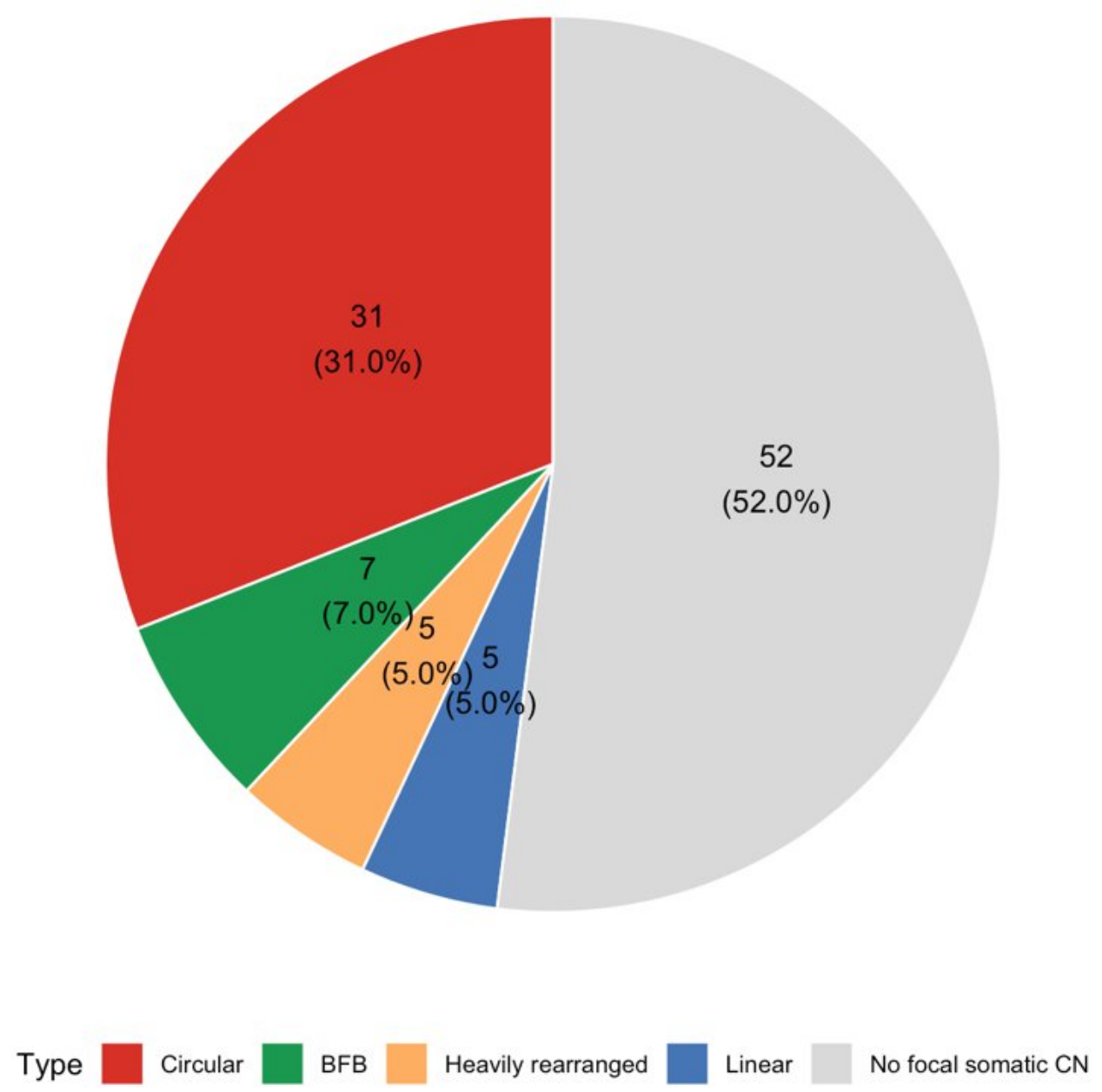

Supplementary Fig. 19. Identification and classification of focal amplifications.

Homologous recombination deficiency (HRD)

– HRD Status

We evaluated the HRD status of 100 GCs using a random forest-based Classifier of HOmologous Recombination Deficiency (CHORD)[31] and Homologous Recombination Deficiency Detect (HRDetect)[32] (Supplementary Table 8a and 8b). In this study, tumors with both the CHORD P_{HRD} score ≥ 0.4 and the HRDetect score ≥ 0.4 were classified as HRD, whereas all others were classified as HRP.

– CHORD

For CHORD-HRD[31] v2.3, we used the VCF files containing somatic SNVs and indels, as well as structural variants (SVs) that were identified.

– HRDetect

For HRDetect[32], we utilized the files that contains segmented copy number variation (CNV) and loss of heterozygosity (LOH) identified by Sequenza, somatic SNVs and indels, and structural variants (SVs).

In this study, tumors with both the CHORD P_{HRD} score ≥ 0.4 and the HRDetect score ≥ 0.4 were classified as HRD. Using these criteria, 4 of 100 GCs (4%) were defined as HRD. The small number of HRD GCs precluded a meaningful survival analysis (*P* for log-rank = 0.21; HR = 3.89 X 10^{-8} (95% CI, 0–infinite); Supplementary Fig. 20).

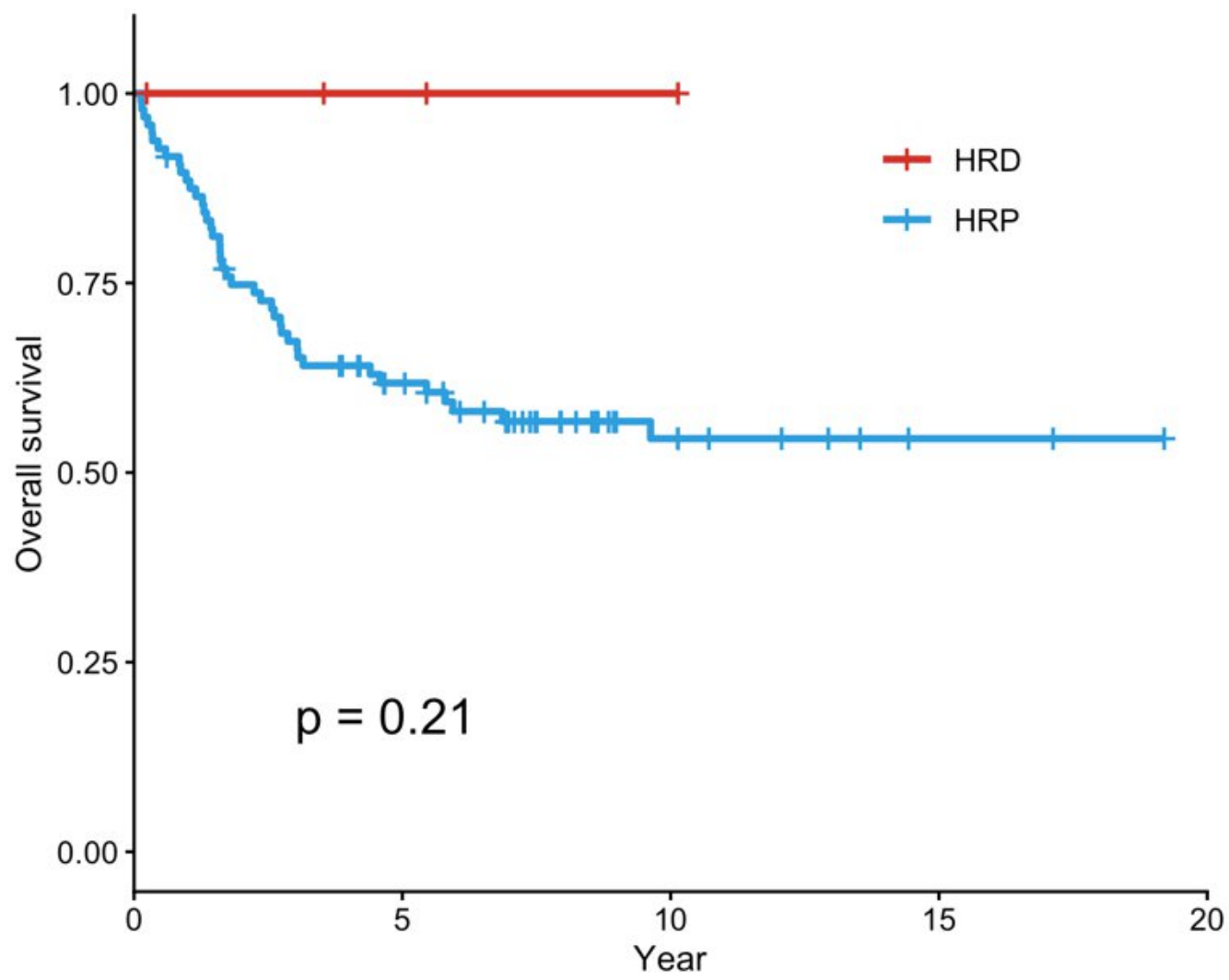

Supplementary Fig. 20. Kaplan-Meier curves for overall survival between HRP (shown in blue) and HRD GCs (shown in red). GC, gastric cancer; HRD, homologous recombination deficiency; HRP, homologous recombination proficiency.

- **Germline mutation contributed to HRD**

When we evaluated if pathogenic germline mutations contributed to HRD, we found no pathogenic germline mutations in HR pathways among our 100 GCs, unlike our prior studies of early-onset GC. In the current dataset of 100 GCs, however, pathogenic germline mutation occurred only in a 63-year-old male patient with diffuse GC harboring MUTYH p.Y125* (c.375T>A) mutation. HRD in the current dataset, therefore, was not attributed to pathogenic germline mutations in HR pathways, but entirely by somatic alterations. Germline variations in the normal samples were identified using GATK v4.1.8.0[3]. HaplotypeCaller and VariantsFiltration modules were used to detect SNPs, with the following filtering criteria (QD < 10.0, MQ < 50.0, FS > 10.0, MQRankSum < -5.0, ReadPosRankSum < -8.0). BaseRecalibrator and ApplyBQSR modules were then used to generate recalibrated BAM files for each normal sample. Finally, HaplotypeCaller module was reapplied to detect variants as follows:

1. Annotate vep version 102
2. Filter out variants from dbSNP 153
3. Filter for protein coding
4. Filter for HIGH impact
5. Filter for variants co-located with those that are rare (< 0.5%) in 1000 Genomes (African, American, Asian, European, South Asian population, 1000 Genome Project and AWS (RRID:SCR_008801)) and NHLBI-ESP (African American, European American population)

MUTYH p.Y125* (c.375T>A) mutation was the only pathogenic germline mutation among the 100 patients in our cohort. No pathogenic germline mutations were identified in HRD genes.

- **HR-related gene alterations and methylation status**

To identify genomic alterations underlying HRD GCs, we investigated somatic mutations, homozygous deletion, SV, and promoter methylation of *BRIP1, BARD1, CHEK1, CHEK2, ATM, ATR, FANCA, FANCC, FANCI, FANCL, RAD50, RAD51, RAD51B, RAD54L, CDK12, PPP2R2A, MRE11, NBN*. Promoter methylation was defined as Δβ > 0.15 and tumor TPM < 3.

– Neoantigen load (NAL) and TMB in HRD vs HRP

Neoantigen load was estimated using only NetMHC[33] option of pVAC-Seq pipeline[34]. HLA typing analysis was conducted using HISAT-genotype[35] and RNA-seq fastq files trimmed by Trimmomatic v0.39[11].
To compare the NAL between HRD (*n* = 4) and HRP (*n* = 66) groups in 70 non-EBV/non-MSI samples, RNA-seq data was trimmed using Trimmomatic, and HLA typing was performed with HISAT-genotype. The HLA typing results, combined with Mutect2 outputs, were used to calculate the neoantigen load (NAL) via the pVAC-Seq pipeline. Mean NAL and TMB were then analyzed according to HRD and HRP status (Supplementary Table 18).

Supplementary Table 18. Mean NAL and TMB according to the HRD status. NAL, neoantigen load, TMB, tumor mutation burden, HRD, homologous recombination deficiency, HRP, homologous recombination proficiency.

	**Neoantigen Load (NAL)**	**Tumor Mutation Burden (TMB)**
HRD (*n* = 4)	2.75	4.35
HRP (*n* = 66)	4.12	11.60
P for *t*-test	0.777	0.448

– RNA-seq expression profiles of HRD

There were 522 genes that were significantly more highly expressed in the four HRD GCs than in the 96 HRP GCs at $P < 0.05$ (Supplementary Table 9). The following 7 KEGG pathways were significantly (FDR < 0.05) enriched in genes that were overexpressed in HRD GCs (Supplementary Table 19).

Supplementary Table 19. KEGG pathways enriched in genes overexpressed in HRD GCs.

No.	KEGG pathway	FDR	Genes
1	hsa04062: Chemokine	<0.001	CCL22/ PTK2B/ CCL23/ CCL11/ ADCY8/ CCR6/ CCL19/ TIAM1/ ITK/ CCL5/ GNGT1/ CXCR3/ CCR7/ CX3CR1/ DOCK2/ CCR2/ ADCY5/ RASGRP2/ PLCG2/ WAS/ GNG7
2	hsa04060: Cytokine–receptor interaction	<0.001	LTA/ CD40LG/ CCL22/ CD27/ BMP10/ CCL23/ CCL11/ GDF7/ NGFR/ CCR6/ CCL19/ EBI3/ IL16/ BMP6/ CCL5/ TNFRSF13B/ CXCR3/ CCR7/ CX3CR1/ CCR2/ TNFSF12/ IL5RA/ IL10RA/ TNFRSF17/ IL12RB1/ IL12B/ TNFRSF8
3	hsa04650: NK cell cytotoxicity	<0.001	NCR2/ PTK2B/ CD247/ ARAF/ CD48/ ITGAL/ PTPN6/ KLRC4-KLRK1/ KLRC3/ ZAP70/ NCR3/ SH2D1A/ PLCG2/ KLRK1
4	hsa04061: Viral–cytokine interaction	<0.001	LTA/ CCL22/ CCL23/ CCL11/ CCR6/ CCL19/ CCL5/ CXCR3/ CCR7/ CX3CR1/ CCR2/ IL10RA
5	hsa05340: Primary immunodeficiency	<0.001	CD79A/ CD40LG/ CD3E/ ZAP70/ TNFRSF13B/ CD3D/ BTK
6	hsa04662: B cell receptor	<0.001	CD79A/ BANK1/ PTPN6/ CD79B/ BLK/ INPP5D/ FCGR2B/ PLCG2/ BTK
7	hsa04659: Th17 cell differentiation	0.001	LTA/ CD40LG/ CCL22/ CD27/ BMP10/ CCL23/ CCL11/ GDF7/ NGFR/ CCR6/ CCL19/ EBI3/ IL16/ BMP6/ CCL5/ TNFRSF13B/ CXCR3/ CCR7/ CX3CR1/ CCR2/ TNFSF12/ IL5RA/ IL10RA/ TNFRSF17/ IL12RB1/ IL12B/ TNFRSF8

– 12-chemokine gene expression

RNA expression profiles of 12 chemokines reflect intratumoral immune reaction in melanoma and lung cancers[36]. To extrapolate potential clinical implication of the sum of RNA expression levels of the 12 chemokines, we downloaded the RNA expression data from a prior publication[37]. We found that the sum of RNA expression levels of the 12 chemokines was higher in clinical responders than in nonresponders (*P* for *t*-test, 0.05; Supplementary Fig. 21a). Thus, our re-analysis of a prior study revealed that the

sum of RNA expression levels of these 12 chemokines was higher in gastric cancers with objective response to pembrolizumab than nonresponders.

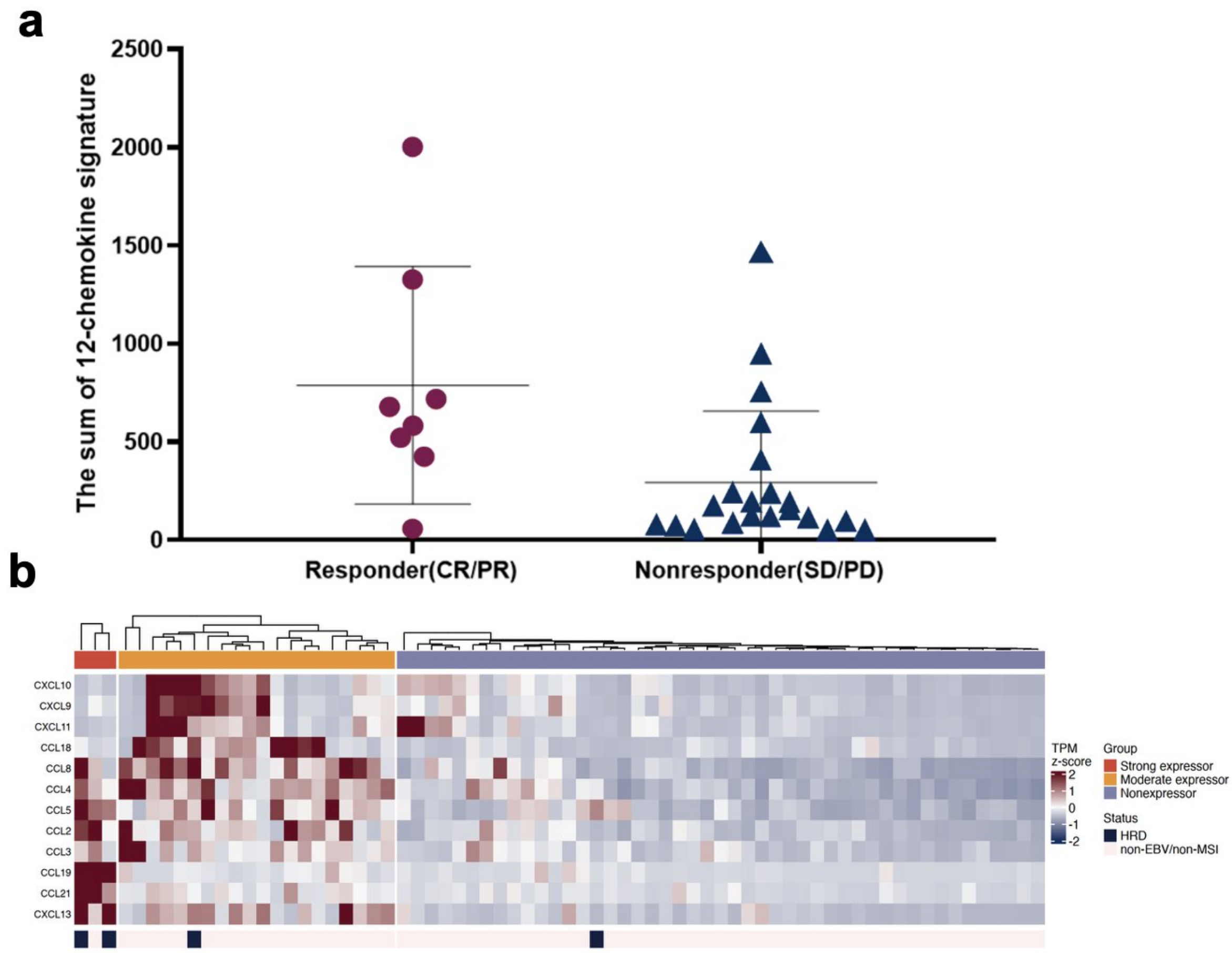

Supplementary Fig. 21. a The sum of the 12-chemokine signature according to objective response to pembrolizumab (CR/PR vs SD/PD) according to the RNA expression data from a prior publication[37]. Mean sum of the 12 chemokines was higher in 12 GCs with objective response to pembrolizumab (CR/PR) than 25 GCs without response (SD/PD) (*P* for *t*-test = 0.05). **b** Hierarchical clustering of the 12-chemokine mRNA expression profiles. Three clusters (non-, moderate-, and strong expressor of the 12 chemokines) were observed. Bottom panel, HRD cases (shown in black)

Therefore, the sum of RNA expression levels of the 12 chemokines[36] was taken as a surrogate indicator of adaptive immune response of our 100 GCs, and the sum was then compared between the HRD and HRP GCs. For this analysis, we excluded MSI and EBV tumors, which are lymphocyte-rich subgroups, from HRP GCs. As a result, the sum of 12 chemokines' RNA expression levels was significantly higher in HRD (*n* = 4) than in HRP (*n* = 66; *P* for *t*-test=0.029; Fig. 6c). The sum also significantly correlated with the P_{HRD} of 70 non-EBV/non-MSI GCs (*R* = 0.35, *P* = 0.003). Other than P_{HRD}, only the coding indel/SNV ratio significantly correlated with sum of 12 chemokines' RNA expression levels (*R* = 0.44, *P* = 0.0001). Collectively, these data suggest a possibility that HRD GC might represent a subgroup that is more responsive to immune checkpoint inhibitor.

These results provide indirect evidence for the association of HRD with clinical response to immunotherapy suggested by our study, given the sum of RNA expression levels of the 12 chemokines was significantly higher in 4 HRD tumors than in 66 non-EBV/non-MSI HRP tumors.

Chi-square test was conducted to address whether 12-chemokine mRNA expression signature was relatively enriched in 4 HRD tumors. According to hierarchical clustering of the 12-chemokine mRNA expression profiles, three clusters were observed (non-, moderate-, and strong expressor of the 12 chemokines)[36] (Supplementary Fig. 21b). HRD GCs were significantly enriched for overexpressors (strong/moderate expressors) of the 12 chemokines (*P* for Chi-square test = 0.02; Supplementary Table 20).

Supplementary Table 20. RNA expression of 12 chemokines

**Type**	**non-EBV/non-MSI (*n* = 70)**	
	**HRD (*n* = 4)**	**HRP (*n* = 66)**
Nonexpressor	1	51
Strong/moderate expressor	3	15
		P value for chi-square test = 0.02

Genomic retrotransposition events

Somatic retrotransposition was evaluated using TraFiC-mem v1.0.0[39] with default settings (Supplementary Table 11a). To account for differences in sequencing depth across samples, retrotransposition counts were normalized by sequencing coverage using the cohort median coverage as a reference.

The number of somatic retrotransposition events was not associated with prognosis of our 100 patients with GC (HR = 1.002; 95% CI: 0.999–1.004; *P* = 0.124).

We next investigated whether retrotransposon integration contributed to the inactivation of protein-coding genes. None of the 13 recurrent SNVs were localized to retrotransposition regions. In addition, 14 LINE-1 (L1) integration events were identified within coding regions across 13 GCs; however, none involved known tumor suppressor genes or DNA repair genes (Supplementary Table 21).

Supplementary Table 21. Retrotransposon integration events in the coding region.

No.	Chromosome	Start	End	Gene name
1	5	122,881,451	122,881,452	*SNX24*
2	12	112,235,899	112,235,900	*HECTD4*
3	7	121,674,424	121,674,425	*PTPRZ1*
4	3	151,475,243	151,475,244	*IGSF10*
5	5	140,208,263	140,208,264	*CYSTM1*
6	7	96,747,090	96,747,091	*ACN9*
7	3	192,988,474	192,988,475	*HRASLS*
8	5	67,590,374	67,590,375	*PIK3R1*
9	9	5,069,106	5,069,107	*JAK2*
10	9	5,069,135	5,069,136	*JAK2*
11	1	242,271,014	242,271,015	*PLD5*
12	11	123,777,954	123,777,955	*OR8D4*
13	7	33,059,268	33,059,269	*NT5C3A*
14	9	27,565,550	27,565,551	*C9orf72*

Because retrotransposition frequently accompanies structural rearrangements, we further characterized retrotransposition-related SVs, which accounted for 83.6% of all retrotransposition events. These consisted predominantly of translocations (61.5%), followed by inversions (9.9%), deletions (6.2%), duplications (5.8%), and insertions (0.1%). Consistent with previous observations that retrotransposition can induce non-allelic homologous recombination, leading to genomic rearrangements including deletions, duplications, and inversions, we examined whether retrotransposition regions overlapped recurrent copy number alterations.

In total, 7 retrotransposition events overlapped five focal amplification regions, including chr8:125,976,787–130,368,582 (containing *MYC*; n = 2), chr19:27,733,473–31,299,292 (containing *CCNE1*; n = 2), and chr7:53,928,203–56,560,299 (containing *EGFR*; n = 1) (Fisher's exact test for overlap between significant amplification regions and retrotransposition regions, *P* = 0.0005;

Supplementary Table 23). In contrast, significant deletion regions did not significantly overlap with retrotransposition regions (*P* for Fisher's exact test = 0.75; Supplementary Table 22).

Collectively, these findings suggest that retrotransposition may contribute to gastric carcinogenesis primarily through oncogene amplification rather than through the inactivation of tumor suppressor genes. Consistent with this interpretation, neither the burden of somatic retrotransposition events nor the RNA expression levels of retrotransposons or HERVs showed significant associations with clinical outcome or intratumoral immune activity, as assessed by the sum of expression levels of 12 chemokines among 70 non-EBV/non-MSI GCs.

Supplementary Table 22. Overlap between significant deletion region and retrotransposition

	Deleted	Not deleted
Retrotransposition	5	5
No retrotransposition	807	1,157

Supplementary Table 23. Overlap between significant amplification region and somatic retrotransposition event.

No.	Type	Chr	Start	End	Overlapping amplification
1	L1	7	54,254,478	54,254,479	chr7:53,928,203–56,560,299 (*EGFR*)
2	L1	8	127,525,688	127,525,689	chr8:125,976,787–130,368,582 (*MYC*)
3	L1	8	127,670,787	127,670,788	chr8:125,976,787–130,368,582 (*MYC*)
4	L1	11	55,321,381	55,321,382	chr11:50,619,294–55,513,987
5	L1	13	112,302,066	112,302,067	chr13:109,925,254–112,850,123
6	L1	19	28,488,501	28,488,502	chr19:27,733,473–31,299,292 (*CCNE1*)
7	SVA	19	29,855,780	29,855,781	chr19:27,733,473–31,299,292 (*CCNE1*)

- **Univariate linear regression analysis predicting genomic retrotransposition events**

Univariate linear regression analysis was performed to evaluate the association between somatic retrotransposition activity (TraFiC) and clinical or genomic variables. Continuous variables were dichotomized at the median unless otherwise specified, including age (62 years), scarLOH (8.5), and aneuploidy score by core (17.5). L1 log2FC was defined as the paired tumor-to-normal log2 fold change in the summed L1 expression for each sample pair. TMB-high tumors were defined using cutoffs of > 40 mutations/Mb. Categorical variables were analyzed using predefined groups. For each variable, the β coefficient and 95% confidence interval (CI) were estimated relative to the reference group (Supplementary Table 24).

Supplementary Table 24. Univariate linear regression analysis of clinical and genomic variables associated with somatic retrotransposition events (TraFiC). β coefficients represent the effect size relative to the reference group. Continuous variables were dichotomized at the median unless otherwise indicated, and n values are shown for each group.

Variable	n	β (95% CI)	*P* value
Gender	Female (n=32) vs Male (n=68)	16.27 (−16.54 to 49.08)	0.328
Age (> 62 years)	High (n=48) vs Low (n=52)	26.56 (−3.76 to 56.89)	0.085
TP53 mutation	Mutant (n=48) vs WT (n=52)	26.68 (−3.65 to 57.00)	0.084
Chromothripsis	Yes (n=22) vs No (n=78)	40.68 (4.45 to 76.91)	**0.028**
EBV	Positive (n=11) vs Negative (n=89)	−32.01 (−80.75 to 16.73)	0.196
ecDNA	Yes (n=31) vs No (n=69)	17.67 (−15.40 to 50.74)	0.292
HRD	Yes (n=4) vs No (n=96)	−30.04 (−108.30 to 48.23)	0.448
CIMP	Yes (n=50) vs No (n=50)	−0.88 (−31.64 to 29.89)	0.955
CN9 subtype	non-CN9 (n=39) vs CN9 (n=61)	23.49 (−7.69 to 54.68)	0.138
scarLOH (> 8.5)	High (n=50) vs Low (n=50)	18.64 (−11.89 to 49.18)	0.229
Hypomethylation (> 20%),	Yes (n=21) vs No (n=79)	61.03 (25.30 to 96.76)	**0.001**
Stage	III–IV (n=70) vs I–II (n=30)	−23.29 (−56.53 to 9.96)	0.168
TMB (> 40/Mb)	High (n=21) vs Low (n=79)	6.28 (−31.47 to 44.02)	0.742
Aneuploidy score (> 17.5)	High (n=50) vs Low (n=50)	15.92 (−14.68 to 46.52)	0.304

- **Multivariate regression analysis predicting genomic retrotransposition events**

Elastic Net regression analysis identified hypomethylation, chromothripsis, and age as key variables associated with somatic retrotransposition (TraFiC) at λ_min, all showing positive effects. Among these, hypomethylation exhibited the strongest effect size, followed by chromothripsis and age, suggesting that these features represent major contributors to increased retrotransposition activity. In contrast, other genomic features, including ecDNA (circular amplification), HRD, and tumor mutational burden, were not retained in the model, indicating limited independent contribution after regularization. These variables were subsequently evaluated using a linear regression model to estimate effect sizes and statistical significance (Fig. 7d).

CRISPR-Cas9 knockout and MTT assay

Methods

CRISPR-Cas9 knockout

We inserted the sgRNA in CRISPR-Cas9 plasmid. First, the small guide RNA (gRNA) was designed using Custom Alt-R™ CRISPR-Cas9 guide RNA service (IDT Inc., IA). After sgRNA oligomer annealing, we inserted the sgRNA oligomer into the CRISPR-Cas9 plasmid using TOPspeed™ DNA Ligation Kit (Enzynomics, Korea). The plasmid was transfected into 293FT (RRID:CVCL_6911) cells using the Lipofectamine® 3000 transfection kit (Invitrogen, MA, USA), along with the lentivirus packaging plasmid psPAX2 (RRID:Addgene_12260) and the envelope plasmid pMD2G. After 72 hours, the lentiviral supernatant was obtained and used to transduce target cells, MKN-45 and SNU-638 for a duration of 72 hours. Subsequently, cells with knockout of the target oncogene were selected through antibiotic selection and utilized for further experiments.

MTT assay

Stable transfectant cells were seeded in a 96-well plate at densities of 5×10^3 cells per well. After seeding, the cells were cultured for 72 hours, and cell proliferation was assessed using the MTT Formazan assay following the manufacturer's protocol (Sigma, MO, USA).

In vivo tumorigenicity

A total of 10^7 stable transfectant cells resuspended in 100 μL of RPMI were injected subcutaneously into the flank of 6-week-old female Balb/c nude mice. Tumor volume was measured by caliper every two days (Supplementary Table 25).

Supplementary Table 25. Number of Balb/c mice that developed tumor after 30 days of injection.

	**Control gRNA**	***BYSL* knockout**	***P* for chi-square**
MKN-45	4/5 (80%)	0/5	0.01
SNU-638	2/5 (40%)	0/5	0.11

Co-occurrence and mutual exclusivity of *BYSL* amplification

To compare 6p21.1 (*BYSL*) amplification status with other recurrent genomic alterations, we analyzed focal high-level amplifications (GISTIC score = 2) across the cohort. Pairwise associations between the 6p21.1 peak and other significant oncogenic or tumor suppressor gene-containing peaks were evaluated using Fisher's exact test. The results, displayed in a sorted heatmap, showed no significant co-occurrence or mutual exclusivity with other major driver events (Supplementary Fig. 22).

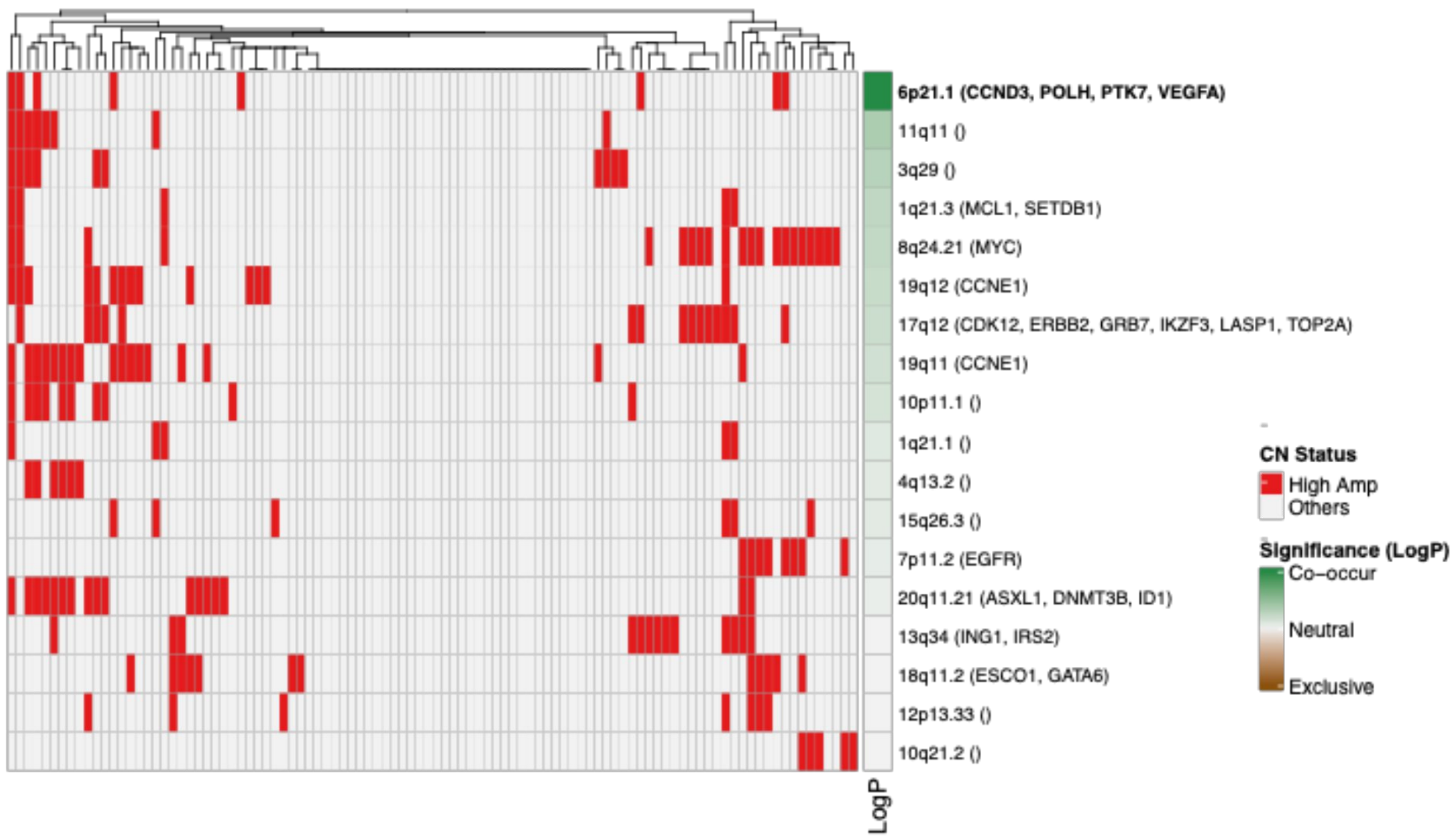

Supplementary Fig. 22. Co-occurrence and mutual exclusivity of 6p21.1 amplification. Pairwise associations between focal high-level amplifications (GISTIC score = 2) are shown as a heatmap. *P* value was assessed using Fisher's exact test. No significant associations were observed for the 6p21.1 (*BYSL*) locus.

Global proteomic profiling analyses of 23 non-EBV/non-MSI GCs

– Methods

Sample preparation
A subset of non-EBV/non-MSI GCs (*n* = 23) were subjected to global proteomic profiling analyses (Supplementary Table 26).

Supplementary Table 26. Clinicopathological variables of 23 non-EBV/non-MSI GCs.

No.	Histology (Lauren)	AJCC Stage (8th Ed)	ORF1p protein	LINE-1 event count	*TP53* mutation (class)	*TP53* mutation (Amino acid Change)
1	Intestinal type	4	1.151	67	Missense	p.K132E
2	Diffuse type	3b	0.706	51	Missense	p.R249W
3	Intestinal type	3a	0.659	22	Frameshift_indel	p.D228*
4	Diffuse type	3c	0.387	55	Missense	p.R248Q
5	Intestinal type	2b	0.322	27	Missense	p.R248W
6	Intestinal type	3a	0.209	15	Nonsense	p.R342*
7	Mixed type	3b	0.091	24	Missense	p.C176Y
8	Intestinal type	3a	-0.029	196	Splice_Site	p.X126_splice
9	Intestinal type	2b	-0.120	5		
10	Mixed type	3a	-0.212	1	Missense	p.R273H
11	Diffuse type (neuroendocrine differentiation)	2b	-0.580	0		
12	Diffuse type	3a	-0.632	15		
13	Intestinal type	3a	-0.930	40		
14	Diffuse type	3a	-0.953	4	Nonsense	p.R213*
15	Diffuse type	3c	-0.984	47	Frameshift_indel	p.T125Sfs*45
16	Intestinal type	3a	-1.116	5		
17	Intestinal type	3c	-1.130	9		
18	Diffuse type	3a	-1.154	20	Nonsense	p.R196*
19	Intestinal type	2b	-1.270	56		
20	Intestinal type	2b	-1.323	53	Missense	p.T155N
21	Intestinal type	3c	-1.372	2	Nonsense	p.S94*
22	Intestinal type	2b	-1.809	84		
23	Diffuse type	3a	-1.836	0		

*Sorted according to decreasing order of ORF1p protein expression level

Protein lysate was isolated from the same tissue powder that was used to isolate genomic DNA and RNA. Macrodissected frozen tumor and adjacent normal tissue were cryopulverized in liquid nitrogen and cryopulverized tissue powder was lysed in 100 mM Tris-HCl buffer (pH 7.6) with 4% SDS and the protease inhibitor cocktail tablet (Roche, Basel, Switzerland). For probe sonication, the Sonicator 3000 (Misonix,

Farmingdale, NY) was used with power level 3 (pulse on for 15 sec and pulse off for 10 sec for 12 times). Lysate was then centrifuged at 16,000 g at 20 °C for 15 min.

Filter-aided sample preparation (FASP)

Microcon-30kDa centrifugal filter unit with ultracel-30 membrane (Merck Millipore, Burlington, MA) was used for the filter-aided sample preparation (FASP). Using 10-plex TMT (Thermo Fisher Scientific, Waltham, MA), we labeled peptides from the tumors and adjacent normal tissue samples (i.e., four patients' samples in each 10-plex set), together with a universal reference comprising tissue of various origin. TMT-labeled peptides were pooled and concentrated using vacuum centrifugation.

Peptide fractionation

Basic pH RPLC fractionation was then conducted to fractionate pooled TMT-labeled peptide samples into 24 peptide fractions using the 1260 Infinity HPLC system (Agilent, Palo Alto, CA). We used the Xbridge C18 analytical column (4.6 mm × 250 mm, 130 Å, 5 μm) and a guard column (4.6 mm × 20 mm, 130 Å, 5 μm) for the peptide separation. We used 10 mM ammonium bicarbonate (AB) in water (pH 10.0) and 10 mM AB in 90 % acetonitrile (pH 10.0), respectively, for solvents A and B. We fractionated peptides using a 115 min-gradient at a flow rate of 500 μL/min [0 % solvent B (10 min), 0→5% solvent B (10 min), 5→35% solvent B (60 min), 35→70% solvent B (15 min), 70% solvent B (10 min), and 70→0% solvent B (10 min)]. Ninety-six 1-min fraction segments (15–110 minutes) were pooled into 24 non-contiguously concatenated peptide fractions, dried, and stored at -80 °C. In collaboration with Dr. Sang Won Lee (Korea University, Seoul, Republic of Korea), each of the 24 peptide fractions (approximately 5 μg) was individually separated by a modified nanoACQUITY UPLC (Waters, Milford, MA) online-coupled to a Q Exactive Orbitrap mass spectrometer (Thermo Fisher Scientific, Waltham, MA).

Mass spectrometry

The nanoACQUITY UPLC system was equipped with six-ports, two-channels, two-position switching valves on which a capillary analytical column (75 μm x 100 cm) and a solid phase extraction (SPE) column (150 μm x 3 cm) were installed.[40] These columns were manufactured by slurry packing of C18 resin (Jupiter, 3 μm, 300 Å; Phenomenex, Torrance, CA). The capillary analytical column temperature was kept at 60 °C. We used 0.1% formic acid in water and 0.1% formic acid in acetonitrile, respectively, for solvents A and B, and a 180 min-gradient (1→40% solvent B over 160 min, 40→80% solvent B over 5 min, 80% solvent B for 10 min and 1% solvent B for 5 min) for profiling. We set the flow rate and the desolvation capillary temperature at 300 nL/min and 250°C, respectively. We acquired full MS scans for the mass range of 400–2000 Th at a resolution of 70,000. We fragmented 10 most abundant ions by data-dependent MS/MS experiments with an isolation window of ± 0.8 Th, an exclusion duration of 30 s, and at a normalized collision energy of 30 for higher-energy collisional dissociation. We acquired the MS/MS scans at a resolution of 17,500 with a fixed first m/z of 100 Th. The maximum ion injection time was 20 ms and 60 ms for full MS and MS/MS scan, respectively. The automatic gain control target value was set at 1.0 × 106 for both MS and MS/MS scans.

Data processing
All LC-MS/MS data were processed by PE-MMR[41] for precursor mass correction and refinement, followed by the MS-GF+[38] search against the human UniProt (RRID:SCR_002380) database. We allowed semi-tryptic cleavage and set the precursor mass tolerance at 10 ppm. Carbamidomethylation of cysteine and TMT modifications at N-terminus of the peptide and lysine were set as static modifications. Oxidation to methionine was set as a variable modification. All MS-GF+ spectral identification files were converted to IDPicker 3[42] index files for the protein assembly. A minimum of 2 unique peptides were required to identify a given protein. TMT reporter ion intensity of each tumor was normalized to that of universal reference, which was a mixture of various tissue origin, that was included in the corresponding 10-plex set of experiment. Missing values were imputed using Perseus.[43]

Pathway analysis
Pathway analysis was conducted using online KEGG (RRID:SCR_012773) pathway analysis tools (https://david.ncifcrf.gov/). KEGG pathway analyses were performed using protein whose abundance positively correlated with that of ORF1p protein (Q9UN81).

– Results

A subset of non-EBV/non-MSI GCs (n = 23) were subjected to global proteomic profiling analyses. Shown below are QC parameters of proteomic profiling data of these 23 patients (Supplementary Fig. 23).

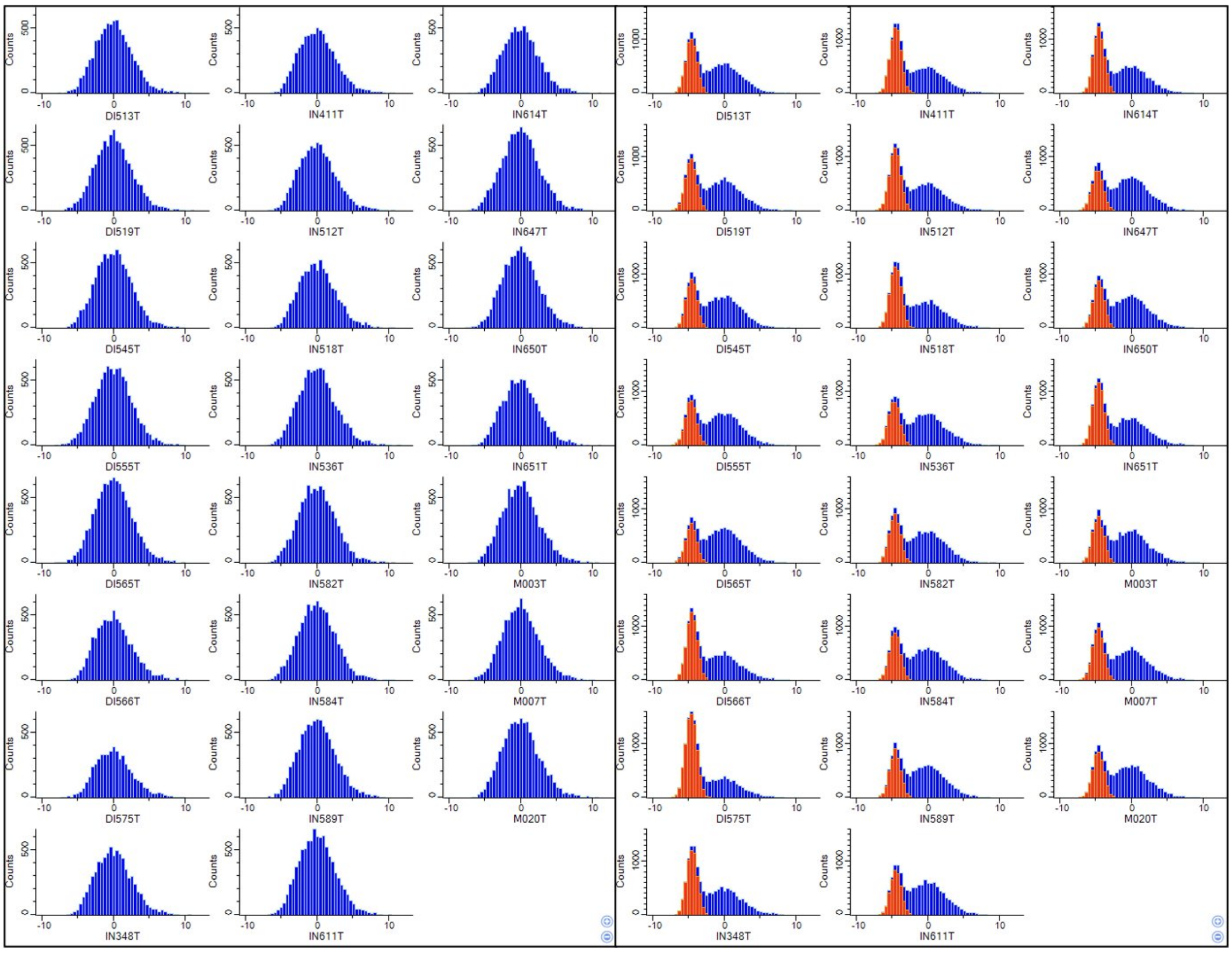

Supplementary Fig. 23. Histogram visualization of data before and after imputation using Perseus. Left Panel (Blue): Data distribution before imputation, Right Panel (Blue and Red): Data distribution after imputation. The red histogram highlights the imputed values using a Gaussian distribution using the Perseus parameters of width = 0.3 and down shift = 1.8.

In this subset of GCs, protein expression level of ORF1p positively correlated with those of 701 proteins ($R > 0.413$; $P < 0.05$) (Supplementary Table 10a). According to DAVID pathway analyses, 24 KEGG pathways were significantly enriched in these 701 ORF1p-correlated proteins (Supplementary Table 27).

Supplementary Table 27. KEGG pathways significantly enriched in ORF1p-correlated proteins.

No.	Term	Benjamini P	FDR
1	hsa03040:Spliceosome	<0.001	<0.001
2	hsa03013:Nucleocytoplasmic transport	<0.001	<0.001
3	hsa05014:Amyotrophic lateral sclerosis	<0.001	<0.001
4	hsa04540:Gap junction	<0.001	<0.001
5	hsa05012:Parkinson disease	<0.001	<0.001
6	hsa05020:Prion disease	<0.001	<0.001
7	hsa03030:DNA replication	<0.001	<0.001
8	hsa05016:Huntington disease	<0.001	<0.001
9	hsa04145:Phagosome	<0.001	<0.001
10	hsa03410:Base excision repair	<0.001	<0.001
11	hsa04814:Motor proteins	<0.001	<0.001
12	hsa03420:Nucleotide excision repair	<0.001	<0.001
13	hsa03050:Proteasome	<0.001	<0.001
14	hsa05010:Alzheimer disease	<0.001	<0.001
15	hsa05132:Salmonella infection	<0.001	<0.001
16	hsa03015:mRNA surveillance pathway	<0.001	<0.001
17	hsa03430:Mismatch repair	0.001	<0.001
18	hsa05130:Pathogenic Escherichia coli infection	0.001	0.001
19	hsa03082:ATP-dependent chromatin remodeling	0.002	0.002
20	hsa04110:Cell cycle	0.002	0.002
21	hsa04612:Antigen processing and presentation	0.002	0.002
22	hsa05022:Pathways of neurodegeneration - multiple diseases	0.004	0.004
23	hsa05169:Epstein-Barr virus infection	0.012	0.011
24	hsa03450:Non-homologous end-joining	0.020	0.018

Given that LINE-1 retrotransposition is sensed as a DNA damaging event through the creation of DNA breaks involving LINE-1-encoded endonuclease,[44] overexpression of *DNA repair, mRNA surveillance, and nucleocytoplasmic transport* protein pathways is not unexpected for ORF1p overexpressing tumors.

To validate the KEGG-based findings using alternative pathway resources, we additionally analyzed immune-related gene sets from the MSigDB[45] database, including Hallmark, GO biological process[46], and Reactome[47] collections (Supplementary Fig. 24). Comparable enrichment patterns were observed across these databases, with consistent associations between LINE-1/ORP1p and immune-related pathways. In particular, Hallmark *INTERFERON GAMMA RESPONSE*, GO terms related to immune activation, and Reactome pathways involved in adaptive immune signaling were enriched in a concordant manner. Overall, these results indicate that the association between LINE-1 retrotransposition and anticancer immunity is reproducible across different pathway databases.

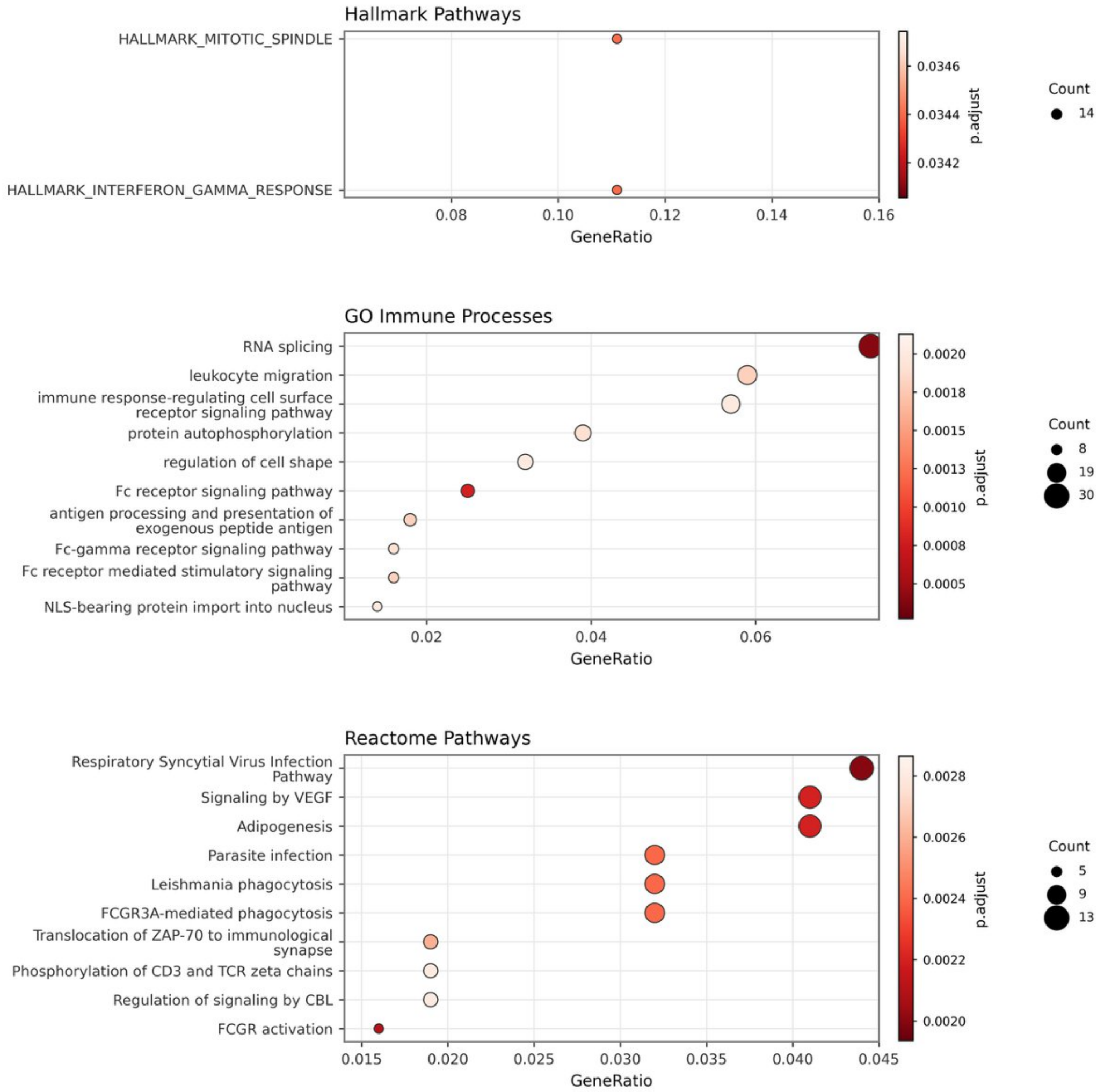

Supplementary Fig. 24. Gene sets were obtained from MSigDB, including Hallmark, Gene Ontology (GO), and Reactome collections.

Global proteomic profiling analyses of an independent dataset of 70 non-EBV/non-MSI early-onset GCs[48]

To validate the aforementioned finding in independent dataset, we reanalyzed our own prior proteomic data from 70 non-EBV/non-MSI early-onset GCs.[48] Since no universal reference was included in this dataset, iTRAQ reporter ion intensity ratio between tumor and adjacent normal tissue of the same patient was taken as the protein abundance. In case the reporter ion intensity was missing, the ratio was substituted as 1. Shown below are clinicopathological variables of 70 early-onset GCs that were negative for EBV and MSI[48] (Supplementary Table 28).

Supplementary Table 28. Clinicopathological variables of 70 non-EBV/non-MSI early-onset GCs (sorted according to decreasing order of ORF1p protein abundance)[48]

No.	Histology (Lauren)	Location	Stage	TP53 mutation	AA change
1	Diffuse	Middle 1/3	IIIB	Frameshift	p.P72fs
2	Diffuse	Distal 1/3	IV	Frameshift	p.T211fs
3	Diffuse	Distal 1/3	IV		
4	Diffuse	Proximal 1/3	IIIB		
5	Diffuse	Distal 1/3	IIB	Nonsense	p.R213*
6	Diffuse	Distal 1/3	IIIA		
7	Diffuse	Proximal 1/3	IIIB		
8	Diffuse	Distal 1/3	IIIB		
9	Diffuse	Distal 1/3	IIIC	Missense	p.G245S
10	Diffuse	Distal 1/3	IIIB	Missense	p.R248Q
11	Diffuse	Distal 1/3	IIIC	Missense	p.R273C
12	Diffuse	Distal 1/3	IIIA		
13	Diffuse	Proximal 1/3	IV		
14	Diffuse	Middle 1/3	IV	Missense	p.L145P
15	Diffuse	Distal 1/3	IIIB	Missense	p.E285K
16	Diffuse	Middle 1/3	IIB	Missense	p.K132M
17	Diffuse	Middle 1/3	IIIB	Missense	p.T125M
18	Diffuse	Proximal 1/3	IV		
19	Diffuse	Proximal 1/3	IIIB		
20	Diffuse	Middle 1/3	IIB		
21	Diffuse	Entire	IIIC		
22	Diffuse	Middle 1/3	IIB		
23	Diffuse	Middle 1/3	IV	InFrameDel	p.P191del
24	Diffuse	Distal 1/3	IIIA		
25	Intestinal	Distal 1/3	IIB		
26	Diffuse	Proximal 1/3	IIIA		
27	Diffuse	Proximal 1/3	IV		
28	Diffuse	Proximal 1/3	IIIA		
29	Intestinal	Middle 1/3	IIB	Missense	p.V272M
30	Diffuse	Distal 1/3	IIB		
31	Diffuse	Middle 1/3	IIB		
32	Diffuse	Middle 1/3	IIIC	Nonsense	p.R213*
33	Diffuse	Proximal 1/3	IV	Missense	p.R282W
34	Diffuse	Distal 1/3	IV		
35	Diffuse	Proximal 1/3	IIIB	Missense	p.C176F
36	Diffuse	Distal 1/3	IA		
37	Diffuse	Distal 1/3	IIB		

38	Diffuse	Middle 1/3	IB	Missense	p.R181H
39	Diffuse	Proximal 1/3	IIB		
40	Diffuse	Middle 1/3	IIB		
41	Diffuse	Middle 1/3	IIIA		
42	Diffuse	Middle 1/3	IIA		
43	Diffuse	Middle 1/3	IIB		
44	Diffuse	Distal 1/3	IIA		
45	Diffuse	Middle 1/3	IIIB		
46	Diffuse	Middle 1/3	IIA		
47	Diffuse	Middle 1/3	IIB		
48	Diffuse	Middle 1/3	IV		
49	Diffuse	Middle 1/3	IIIB	Missense	p.E286K
50	Diffuse	Proximal 1/3	IIIA	missense	p.R248Q
51	Diffuse	Distal 1/3	IV	Missense	p.R282W
52	Diffuse	Middle 1/3	IIIB	Missense	p.V274A
53	Diffuse	Proximal 1/3	IIIC	Missense	p.G245S
54	Diffuse	Distal 1/3	IIIB		
55	Diffuse	Middle 1/3	IIIB		
56	Diffuse	Middle 1/3	IIIB		
57	Diffuse	Proximal 1/3	IV	Missense	p.V274A
58	Diffuse	Middle 1/3	IIIC		
59	Diffuse	Middle 1/3	IV	Missense	p.R248Q
60	Diffuse	Entire	IV	Nonsense	p.R306*
61	Diffuse	Middle 1/3	IV		
62	Diffuse	Proximal 1/3	IIA		
63	Diffuse	Middle 1/3	IV		
64	Diffuse	Middle 1/3	IIA	Missense	p.L350P
65	Diffuse	Middle 1/3	IA		
66	Diffuse	Middle 1/3	IIIB		
67	Diffuse	Distal 1/3	IIIC	Nonsense	p.R213*
68	Diffuse	Distal 1/3	IIB		
69	Diffuse	Distal 1/3	IIA		
70	Diffuse	Proximal 1/3	IIIB		

In the 70 early-onset GCs that were negative for EBV and MSI[48], 3,140 proteins correlated with ORF1p in protein expression ratio ($R > 0.2355$; $P < 0.05$). DAVID pathway analyses were again performed using protein whose abundance was positively correlated with that of ORF1p protein (Q9UN81). We used the same *P* cutoff as was used for the aforementioned subset of 23 non-EBV/non-MSI GCs (Supplementary Table 10b). FDR < 0.05 was used as the cutoff to define significant enrichment (Supplementary Table 29).

Supplementary Table 29. ORF1p-correlated proteins among 70 non-EBV/non-MSI early-onset GCs [48].

No.	KEGG pathway	Benjamini P	FDR
1	hsa05132:Salmonella infection	<0.001	<0.001
2	hsa05131:Shigellosis	<0.001	<0.001
3	hsa05135:Yersinia infection	<0.001	<0.001
4	hsa03013:Nucleocytoplasmic transport	<0.001	<0.001
5	hsa04144:Endocytosis	<0.001	<0.001
6	hsa04613:Neutrophil extracellular trap formation	<0.001	<0.001
7	hsa05169:Epstein-Barr virus infection	<0.001	<0.001
8	hsa05203:Viral carcinogenesis	<0.001	<0.001
9	hsa04140:Autophagy - animal	<0.001	<0.001
10	hsa05164:Influenza A	<0.001	<0.001
11	hsa04666:Fc gamma R-mediated phagocytosis	<0.001	<0.001
12	hsa05130:Pathogenic Escherichia coli infection	<0.001	<0.001
13	hsa05220:Chronic myeloid leukemia	<0.001	<0.001
14	hsa04145:Phagosome	<0.001	<0.001
15	hsa05034:Alcoholism	<0.001	<0.001
16	hsa03015:mRNA surveillance pathway	<0.001	<0.001
17	hsa05417:Lipid and atherosclerosis	<0.001	<0.001
18	hsa05322:Systemic lupus erythematosus	<0.001	<0.001
19	hsa04120:Ubiquitin mediated proteolysis	<0.001	<0.001
20	hsa04621:NOD-like receptor signaling pathway	<0.001	<0.001
21	hsa04722:Neurotrophin signaling pathway	<0.001	<0.001
22	hsa05170:Human immunodeficiency virus 1 infection	<0.001	<0.001
23	hsa05145:Toxoplasmosis	<0.001	<0.001
24	hsa05211:Renal cell carcinoma	<0.001	<0.001
25	hsa03082:ATP-dependent chromatin remodeling	<0.001	<0.001
26	hsa04380:Osteoclast differentiation	<0.001	<0.001
27	hsa05152:Tuberculosis	<0.001	<0.001
28	hsa03410:Base excision repair	<0.001	<0.001
29	hsa04210:Apoptosis	<0.001	<0.001
30	hsa04664:Fc epsilon RI signaling pathway	<0.001	<0.001
31	hsa05212:Pancreatic cancer	<0.001	<0.001
32	hsa05161:Hepatitis B	<0.001	<0.001
33	hsa04370:VEGF signaling pathway	<0.001	<0.001
34	hsa05140:Leishmaniasis	<0.001	<0.001
35	hsa00511:Other glycan degradation	<0.001	<0.001
36	hsa05160:Hepatitis C	<0.001	<0.001
37	hsa05223:Non-small cell lung cancer	<0.001	<0.001
38	hsa05100:Bacterial invasion of epithelial cells	0.001	<0.001

39	hsa03420:Nucleotide excision repair	0.001	<0.001
40	hsa03250:Viral life cycle - HIV-1	0.001	0.001
41	hsa05221:Acute myeloid leukemia	0.001	0.001
42	hsa04910:Insulin signaling pathway	0.001	0.001
43	hsa00562:Inositol phosphate metabolism	0.001	0.001
44	hsa04062:Chemokine signaling pathway	0.001	0.001
45	hsa04150:mTOR signaling pathway	0.001	0.001
46	hsa04510:Focal adhesion	0.001	0.001
47	hsa03018:RNA degradation	0.001	0.001
48	hsa03040:Spliceosome	0.001	0.001
49	hsa04662:B cell receptor signaling pathway	0.002	0.001
50	hsa05166:Human T-cell leukemia virus 1 infection	0.002	0.001
51	hsa05162:Measles	0.002	0.001
52	hsa04625:C-type lectin receptor signaling pathway	0.002	0.001
53	hsa05163:Human cytomegalovirus infection	0.002	0.001
54	hsa04520:Adherens junction	0.002	0.002
55	hsa04070:Phosphatidylinositol signaling system	0.003	0.002
56	hsa05010:Alzheimer disease	0.003	0.002
57	hsa04810:Regulation of actin cytoskeleton	0.003	0.002
58	hsa03030:DNA replication	0.003	0.002
59	hsa04064:NF-kappa B signaling pathway	0.004	0.003
60	hsa05214:Glioma	0.004	0.003
61	hsa04142:Lysosome	0.004	0.003
62	hsa05215:Prostate cancer	0.005	0.004
63	hsa04148:Efferocytosis	0.006	0.004
64	hsa04611:Platelet activation	0.007	0.005
65	hsa04931:Insulin resistance	0.007	0.005
66	hsa04217:Necroptosis	0.008	0.005
67	hsa04015:Rap1 signaling pathway	0.009	0.006
68	hsa05202:Transcriptional misregulation in cancer	0.009	0.006
69	hsa05235:PD-L1 expression and PD-1 checkpoint pathway in cancer	0.011	0.008
70	hsa05218:Melanoma	0.011	0.008
71	hsa04110:Cell cycle	0.012	0.008
72	hsa04072:Phospholipase D signaling pathway	0.012	0.008
73	hsa05167:Kaposi sarcoma-associated herpesvirus infection	0.012	0.008
74	hsa03430:Mismatch repair	0.015	0.010
75	hsa04014:Ras signaling pathway	0.017	0.012
76	hsa04720:Long-term potentiation	0.017	0.012
77	hsa04218:Cellular senescence	0.017	0.012
78	hsa05231:Choline metabolism in cancer	0.021	0.015

79	hsa04919:Thyroid hormone signaling pathway	0.022	0.015
80	hsa04660:T cell receptor signaling pathway	0.022	0.015
81	hsa04136:Autophagy - other	0.023	0.016
82	hsa04620:Toll-like receptor signaling pathway	0.023	0.016
83	hsa03022:Basal transcription factors	0.024	0.016
84	hsa05205:Proteoglycans in cancer	0.025	0.017
85	hsa04933:AGE-RAGE signaling pathway in diabetic complications	0.026	0.018
86	hsa04540:Gap junction	0.026	0.018
87	hsa04130:SNARE interactions in vesicular transport	0.029	0.020
88	hsa01524:Platinum drug resistance	0.029	0.020
89	hsa04330:Notch signaling pathway	0.029	0.020
90	hsa05222:Small cell lung cancer	0.029	0.020
91	hsa05230:Central carbon metabolism in cancer	0.030	0.021
92	hsa01521:EGFR tyrosine kinase inhibitor resistance	0.031	0.021
93	hsa03050:Proteasome	0.034	0.023
94	hsa04622:RIG-I-like receptor signaling pathway	0.034	0.024
95	hsa03020:RNA polymerase	0.034	0.024
96	hsa05213:Endometrial cancer	0.034	0.024
97	hsa05022:Pathways of neurodegeneration - multiple diseases	0.035	0.024
98	hsa04012:ErbB signaling pathway	0.036	0.025
99	hsa05014:Amyotrophic lateral sclerosis	0.037	0.026
100	hsa04010:MAPK signaling pathway	0.037	0.026
101	hsa05210:Colorectal cancer	0.041	0.028
102	hsa05017:Spinocerebellar ataxia	0.044	0.031
103	hsa04650:Natural killer cell mediated cytotoxicity	0.051	0.035
104	hsa05200:Pathways in cancer	0.052	0.035
105	hsa04530:Tight junction	0.052	0.036
106	hsa04935:Growth hormone synthesis, secretion and action	0.052	0.036
107	hsa04071:Sphingolipid signaling pathway	0.052	0.036
108	hsa04917:Prolactin signaling pathway	0.053	0.036
109	hsa05219:Bladder cancer	0.059	0.040
110	hsa04068:FoxO signaling pathway	0.060	0.041
111	hsa04668:TNF signaling pathway	0.062	0.042
112	hsa01240:Biosynthesis of cofactors	0.062	0.043
113	hsa04216:Ferroptosis	0.070	0.048

Supplementary references

1 Li, H. & Durbin, R. Fast and accurate short read alignment with Burrows-Wheeler transform. *Bioinformatics* **25**, 1754-1760 (2009). https://doi.org/10.1093/bioinformatics/btp324

2 Li, H. *et al.* The Sequence Alignment/Map format and SAMtools. *Bioinformatics* **25**, 2078-2079 (2009). https://doi.org/10.1093/bioinformatics/btp352

3 McKenna, A. *et al.* The Genome Analysis Toolkit: a MapReduce framework for analyzing next-generation DNA sequencing data. *Genome Res* **20**, 1297-1303 (2010). https://doi.org/10.1101/gr.107524.110

4 Lawrence, M. S. *et al.* Discovery and saturation analysis of cancer genes across 21 tumour types. *Nature* **505**, 495-501 (2014). https://doi.org/10.1038/nature12912

5 McLaren, W. *et al.* The Ensembl Variant Effect Predictor. *Genome Biol* **17**, 122 (2016). https://doi.org/10.1186/s13059-016-0974-4

6 Favero, F. *et al.* Sequenza: allele-specific copy number and mutation profiles from tumor sequencing data. *Ann Oncol* **26**, 64-70 (2015). https://doi.org/10.1093/annonc/mdu479

7 Mermel, C. H. *et al.* GISTIC2.0 facilitates sensitive and confident localization of the targets of focal somatic copy-number alteration in human cancers. *Genome Biol* **12**, R41 (2011). https://doi.org/10.1186/gb-2011-12-4-r41

8 Rausch, T. *et al.* DELLY: structural variant discovery by integrated paired-end and split-read analysis. *Bioinformatics* **28**, i333-i339 (2012). https://doi.org/10.1093/bioinformatics/bts378

9 Wala, J. A. *et al.* SvABA: genome-wide detection of structural variants and indels by local assembly. *Genome Res* **28**, 581-591 (2018). https://doi.org/10.1101/gr.221028.117

10 Chen, X. *et al.* Manta: rapid detection of structural variants and indels for germline and cancer sequencing applications. *Bioinformatics* **32**, 1220-1222 (2016). https://doi.org/10.1093/bioinformatics/btv710

11 Bolger, A. M., Lohse, M. & Usadel, B. Trimmomatic: a flexible trimmer for Illumina sequence data. *Bioinformatics* **30**, 2114-2120 (2014). https://doi.org/10.1093/bioinformatics/btu170

12 Dobin, A. *et al.* STAR: ultrafast universal RNA-seq aligner. *Bioinformatics* **29**, 15-21 (2013). https://doi.org/10.1093/bioinformatics/bts635

13 Li, B. & Dewey, C. N. RSEM: accurate transcript quantification from RNA-Seq data with or without a reference genome. *BMC Bioinformatics* **12**, 323 (2011). https://doi.org/10.1186/1471-2105-12-323

14 Flicek, P. *et al.* Ensembl 2013. *Nucleic Acids Res* **41**, D48-55 (2013). https://doi.org/10.1093/nar/gks1236

15 Ooi, W. F. *et al.* Integrated paired-end enhancer profiling and whole-genome sequencing reveals recurrent CCNE1 and IGF2 enhancer hijacking in primary gastric adenocarcinoma. *Gut* **69**, 1039-1052 (2020). https://doi.org/10.1136/gutjnl-2018-317612

16 McPherson, A. *et al.* deFuse: an algorithm for gene fusion discovery in tumor RNA-Seq data. *PLoS Comput Biol* **7**, e1001138 (2011). https://doi.org/10.1371/journal.pcbi.1001138

17 Krueger, F. & Andrews, S. R. Bismark: a flexible aligner and methylation caller for Bisulfite-Seq applications. *Bioinformatics* **27**, 1571-1572 (2011). https://doi.org/10.1093/bioinformatics/btr167

18 Sasiain, I., Nacer, D. F., Aine, M., Veerla, S. & Staaf, J. Tumor purity estimated from bulk DNA methylation can be used for adjusting beta values of individual samples to better reflect tumor biology. *NAR Genom Bioinform* **6**, lqae146 (2024). https://doi.org/10.1093/nargab/lqae146

19 Weisenberger, D. J. *et al.* CpG island methylator phenotype underlies sporadic microsatellite instability and is tightly associated with BRAF mutation in colorectal cancer. *Nat Genet* **38**, 787-793 (2006). https://doi.org/10.1038/ng1834
20 Alexandrov, L. B. *et al.* Signatures of mutational processes in human cancer. *Nature* **500**, 415-421 (2013). https://doi.org/10.1038/nature12477
21 Diaz-Gay, M. *et al.* Assigning mutational signatures to individual samples and individual somatic mutations with SigProfilerAssignment. *Bioinformatics* **39** (2023). https://doi.org/10.1093/bioinformatics/btad756
22 Yoshihara, K. *et al.* Inferring tumour purity and stromal and immune cell admixture from expression data. *Nat Commun* **4**, 2612 (2013). https://doi.org/10.1038/ncomms3612
23 Newman, A. M. *et al.* Robust enumeration of cell subsets from tissue expression profiles. *Nat Methods* **12**, 453-457 (2015). https://doi.org/10.1038/nmeth.3337
24 Boland, C. R. *et al.* A National Cancer Institute Workshop on Microsatellite Instability for cancer detection and familial predisposition: development of international criteria for the determination of microsatellite instability in colorectal cancer. *Cancer Res* **58**, 5248-5257 (1998).
25 Cancer Genome Atlas Research, N. Comprehensive molecular characterization of gastric adenocarcinoma. *Nature* **513**, 202-209 (2014). https://doi.org/10.1038/nature13480
26 Cortes-Ciriano, I. *et al.* Comprehensive analysis of chromothripsis in 2,658 human cancers using whole-genome sequencing. *Nat Genet* **52**, 331-341 (2020). https://doi.org/10.1038/s41588-019-0576-7
27 Forment, J. V., Kaidi, A. & Jackson, S. P. Chromothripsis and cancer: causes and consequences of chromosome shattering. *Nat Rev Cancer* **12**, 663-670 (2012). https://doi.org/10.1038/nrc3352
28 Talevich, E., Shain, A. H., Botton, T. & Bastian, B. C. CNVkit: Genome-Wide Copy Number Detection and Visualization from Targeted DNA Sequencing. *PLoS Comput Biol* **12**, e1004873 (2016). https://doi.org/10.1371/journal.pcbi.1004873
29 Deshpande, V. *et al.* Exploring the landscape of focal amplifications in cancer using AmpliconArchitect. *Nat Commun* **10**, 392 (2019). https://doi.org/10.1038/s41467-018-08200-y
30 Luebeck, J. *et al.* Extrachromosomal DNA in the cancerous transformation of Barrett's oesophagus. *Nature* **616**, 798-805 (2023). https://doi.org/10.1038/s41586-023-05937-5
31 Nguyen, L., J, W. M. M., Van Hoeck, A. & Cuppen, E. Pan-cancer landscape of homologous recombination deficiency. *Nat Commun* **11**, 5584 (2020). https://doi.org/10.1038/s41467-020-19406-4
32 Davies, H. *et al.* HRDetect is a predictor of BRCA1 and BRCA2 deficiency based on mutational signatures. *Nat Med* **23**, 517-525 (2017). https://doi.org/10.1038/nm.4292
33 Andreatta, M. & Nielsen, M. Gapped sequence alignment using artificial neural networks: application to the MHC class I system. *Bioinformatics* **32**, 511-517 (2016). https://doi.org/10.1093/bioinformatics/btv639
34 Hundal, J. *et al.* pVAC-Seq: A genome-guided in silico approach to identifying tumor neoantigens. *Genome Med* **8**, 11 (2016). https://doi.org/10.1186/s13073-016-0264-5
35 Kim, D., Paggi, J. M., Park, C., Bennett, C. & Salzberg, S. L. Graph-based genome alignment and genotyping with HISAT2 and HISAT-genotype. *Nat Biotechnol* **37**, 907-915 (2019). https://doi.org/10.1038/s41587-019-0201-4
36 Messina, J. L. *et al.* 12-Chemokine gene signature identifies lymph node-like structures in melanoma: potential for patient selection for immunotherapy? *Sci Rep* **2**, 765 (2012). https://doi.org/10.1038/srep00765
37 Kim, S. T. *et al.* Comprehensive molecular characterization of clinical responses to PD-1 inhibition in metastatic gastric cancer. *Nat Med* **24**, 1449-1458 (2018).

https://doi.org/10.1038/s41591-018-0101-z
38 Kim, S. & Pevzner, P. A. MS-GF+ makes progress towards a universal database search tool for proteomics. *Nat Commun* **5**, 5277 (2014). https://doi.org/10.1038/ncomms6277
39 Rodriguez-Martin, B. *et al.* Pan-cancer analysis of whole genomes identifies driver rearrangements promoted by LINE-1 retrotransposition. *Nat Genet* **52**, 306-319 (2020). https://doi.org/10.1038/s41588-019-0562-0
40 Lee, H. *et al.* A fully automated dual-online multifunctional ultrahigh pressure liquid chromatography system for high-throughput proteomics analysis. *J Chromatogr A* **1329**, 83-89 (2014). https://doi.org/10.1016/j.chroma.2013.12.084
41 Shin, B. *et al.* Postexperiment monoisotopic mass filtering and refinement (PE-MMR) of tandem mass spectrometric data increases accuracy of peptide identification in LC/MS/MS. *Mol Cell Proteomics* **7**, 1124-1134 (2008). https://doi.org/10.1074/mcp.M700419-MCP200
42 Zhang, B., Chambers, M. C. & Tabb, D. L. Proteomic parsimony through bipartite graph analysis improves accuracy and transparency. *J Proteome Res* **6**, 3549-3557 (2007). https://doi.org/10.1021/pr070230d
43 Tyanova, S., Temu, T. & Cox, J. The MaxQuant computational platform for mass spectrometry-based shotgun proteomics. *Nat Protoc* **11**, 2301-2319 (2016). https://doi.org/10.1038/nprot.2016.136
44 Belgnaoui, S. M., Gosden, R. G., Semmes, O. J. & Haoudi, A. Human LINE-1 retrotransposon induces DNA damage and apoptosis in cancer cells. *Cancer Cell Int* **6**, 13 (2006). https://doi.org/10.1186/1475-2867-6-13
45 Subramanian, A. *et al.* Gene set enrichment analysis: a knowledge-based approach for interpreting genome-wide expression profiles. *Proc Natl Acad Sci U S A* **102**, 15545-15550 (2005). https://doi.org/10.1073/pnas.0506580102
46 Ashburner, M. *et al.* Gene ontology: tool for the unification of biology. The Gene Ontology Consortium. *Nat Genet* **25**, 25-29 (2000). https://doi.org/10.1038/75556
47 Croft, D. *et al.* The Reactome pathway knowledgebase. *Nucleic Acids Res* **42**, D472-477 (2014). https://doi.org/10.1093/nar/gkt1102
48 Mun, D. G. *et al.* Proteogenomic Characterization of Human Early-Onset Gastric Cancer. *Cancer Cell* **35**, 111-124 e110 (2019). https://doi.org/10.1016/j.ccell.2018.12.003
